# Optimal Design of Low-Density SNP Arrays for Genomic Prediction: Algorithm and Applications

**DOI:** 10.1371/journal.pone.0161719

**Published:** 2016-09-01

**Authors:** Xiao-Lin Wu, Jiaqi Xu, Guofei Feng, George R. Wiggans, Jeremy F. Taylor, Jun He, Changsong Qian, Jiansheng Qiu, Barry Simpson, Jeremy Walker, Stewart Bauck

**Affiliations:** 1 Bioinformatics and Biostatistics, GeneSeek (a Neogen Company), Lincoln, Nebraska, United States of America; 2 Department of Statistics, University of Nebraska, Lincoln, Nebraska, United States of America; 3 Animal Genomics and Improvement Laboratory, Agricultural Research Service, United States Department of Agriculture, Beltsville, Maryland, United States of America; 4 Division of Animal Sciences, University of Missouri, Columbia, Missouri, United States of America; 5 College of Animal Sciences and Technology, Hunan Agricultural University, Changsha, China; 6 Marketing and Business Development, Neogen Bio-Scientific Technology (Shanghai) Company Ltd., Shanghai, China; Jaypee University of Information Technology, INDIA

## Abstract

Low-density (LD) single nucleotide polymorphism (SNP) arrays provide a cost-effective solution for genomic prediction and selection, but algorithms and computational tools are needed for the optimal design of LD SNP chips. A multiple-objective, local optimization (MOLO) algorithm was developed for design of optimal LD SNP chips that can be imputed accurately to medium-density (MD) or high-density (HD) SNP genotypes for genomic prediction. The objective function facilitates maximization of non-gap map length and system information for the SNP chip, and the latter is computed either as locus-averaged (LASE) or haplotype-averaged Shannon entropy (HASE) and adjusted for uniformity of the SNP distribution. HASE performed better than LASE with ≤1,000 SNPs, but required considerably more computing time. Nevertheless, the differences diminished when >5,000 SNPs were selected. Optimization was accomplished conditionally on the presence of SNPs that were obligated to each chromosome. The frame location of SNPs on a chip can be either uniform (evenly spaced) or non-uniform. For the latter design, a tunable empirical Beta distribution was used to guide location distribution of frame SNPs such that both ends of each chromosome were enriched with SNPs. The SNP distribution on each chromosome was finalized through the objective function that was locally and empirically maximized. This MOLO algorithm was capable of selecting a set of approximately evenly-spaced and highly-informative SNPs, which in turn led to increased imputation accuracy compared with selection solely of evenly-spaced SNPs. Imputation accuracy increased with LD chip size, and imputation error rate was extremely low for chips with ≥3,000 SNPs. Assuming that genotyping or imputation error occurs at random, imputation error rate can be viewed as the upper limit for genomic prediction error. Our results show that about 25% of imputation error rate was propagated to genomic prediction in an Angus population. The utility of this MOLO algorithm was also demonstrated in a real application, in which a 6K SNP panel was optimized conditional on 5,260 obligatory SNP selected based on SNP-trait association in U.S. Holstein animals. With this MOLO algorithm, both imputation error rate and genomic prediction error rate were minimal.

## Introduction

Arrays of SNPs (or SNP chips) are a type of DNA microarray for detecting SNP genotypes, which are the most frequent class of variation in genomes. As of January 2016, the National Center for Biotechnology Information’s dbSNP database [1] included approximately 161 million human, 21 million chicken, 60 million porcine, and 104 million bovine SNPs. Because of the utility of SNPs as genetic markers, they have been widely used in biological research, drug therapy design, cancer research, parentage testing, mapping of quantitative trait loci, and genomic prediction and selection. Over the years, SNP arrays have evolved to be the common thread in an extremely productive synergistic relationship between advances in biological understanding, computational methodology, and technological development of the arrays themselves, each helping to drive advances in the others [2].

Owing to advances in high-throughput sequencing and SNP genotyping, HD SNP chips are now available for many species (e.g., [3–6]). The performance of HD SNP chips is typically evaluated in terms of their coverage, efficiencies, and cost-benefit ratio [7]. A LD SNP chip can often be considerably cheaper than a MD- or HD-SNP chip, and hence the reduced cost in genotyping can be appealingly substantial. The term “coverage” describes the fraction of all SNPs in a genomic region that can be captured by the chip. SNP chip efficiency is typically measured by two criteria: linkage disequilibrium efficiency and non-taggable SNP efficiency. A taggable SNP refers to a SNP for which genotypes in a reference population reach a predefined simple pairwise-correlation threshold with genotypes for at least one other SNP in the same population. Hence, linkage disequilibrium efficiency evaluates the fraction of all taggable SNPs that are tagged, whereas non-taggable SNP efficiency measures how well a chip covers non-taggable SNPs at a predefined linkage disequilibrium threshold. The third criterion, cost-benefit ratio, facilitates the direct comparison of chips in terms of their costs versus gains. From a practical viewpoint, the chip with the greatest utility is one that enables genotyping the greatest number of individuals at the necessary coverage. Although HD SNP chips provide the best coverage of genomes, they may not be cost-effective in view of the balance between increased prediction accuracy (and hence increased genetic gain) and chip price. Currently, HD SNP chips are still too expensive for most agricultural genomic applications.

Consequently, interest has increased in the use of LD SNP chips as a cost-effective solution to genomic prediction and selection (e.g., [8–12]). This trend is evident from a battery of bovine SNP chips which have developed for genomic prediction and selection over the past 10 years. In 2008, Illumina launched its BovineSNP50 Genotyping BeadChip [13] with 54,001SNPs and an initial cost of about 250 US dollars per sample as the first HD array for use in genomic evaluation by the US dairy and beef industries; a subset of approximately 45,000 variable SNPs was actually used in genomic evaluation of each breed. Two year later, the Illumina BovineHD Genotyping BeadChip [14] with more than 777,000 SNPs was released at a much higher price but did not result in significant improvement in the accuracy of genetic evaluation [15]. In 2011, the lower cost Illumina BovineLD Genotyping BeadChip [16] began to be used. Genotypes from its approximately 7,000 SNPs were imputed to 45,000 SNPs for genomic evaluation by the US dairy industry. In 2012, GeneSeek, a Neogen company, began selling the GeneSeek Genomic Profiler (GGP) LD bovine SNP chip; the GGP LD chip has the same SNPs as the BovineLD chip, plus additional 1,800 SNPs for increased imputation accuracy, and also include single-gene tests for causal variants [17]. Version 3 of the GGP LD bovine SNP chip was released in 2014 at the same price as previous versions but included 1,900 additional SNPs. In 2015, GeneSeek announced the release of version 4 of the GGP LD bovine SNP chip, which now assays over 30,000 publically available SNPs [18].

Two basic strategies have been proposed to make use of LD SNP chips in genomic prediction and selection. The most straightforward strategy is to select a subset of SNPs by some variable selection approach for genomic prediction [9]. With this approach, different SNP chips must be designed for different traits because selected SNPs vary with traits. A major disadvantage of this approach is that the loss of prediction accuracy arising from the use of the limited number of SNPs for genomic prediction can be substantial large [9,19]. As a matter of fact, the efficiency of a trait-specific LD SNP chip depends critically on the linkage disequilibrium between the SNPs with large estimated effects and the true causative loci that affect the trait of interest. In addition, because various LD chips need to be manufactured for different traits, the overhead cost for designing the LD chips and for maintaining the manufacturer’s minimum number of chips that must be ordered tends to offset or even exceed reduction in the cost of genotyping the remaining SNPs. The second strategy involves the selection of SNPs with approximately equal spacing, either with or without optimization (or use of a threshold) for minor allele frequencies (MAFs) as the SNPs are selected for the LD chip [8,9,20], which is followed by imputation of LD genotypes to MD or HD SNP genotypes for genomic prediction. This approach eliminates the need for trait-specific LD SNP chips, and loss of prediction accuracy is often negligible when imputation accuracy is sufficiently high [21]. Imputation accuracy generally depends on the number of reference animals, the number of SNPs, and the realized linkage disequilibrium among those SNPs; the extent of genetic relationship between members of the target and reference populations also is important.

Although LD chips provide a cost-effective solution to the implementation of genomic prediction and selection, their optimal design has not been adequately addressed. No commonly accepted algorithm has been proposed for the optimized design of LD chips, and no standard protocol has been presented to guide that procedure. In reality, the *ad-hoc* procedure for designing LD chips has been almost entirely subject to the processes of each user and the design specificities of each application. For example, chip optimizations to date have often been oriented towards the use of evenly spaced markers [8] with some emphasis on the maximization of MAFs [16]. In the design of LD chips, however, objectives to be optimized are not limited to location and allele frequencies but they also include, for examples, SNP location distributions (uniform vs. non-uniform), inclusion of pre-selected (obligatory) SNPs, and the need to determine sex, parentage, Y-chromosome haplotypes, subspecies, and maternal lineages. SNP quality and fidelity criteria for robust reproducibility, SNP coverage and intervals, and numbers and length of gaps are also important considerations. Optimal design of LD SNP chips for agricultural genomic applications is a problem that requires joint optimization of multiple objectives. More often than not, a list of pre-identified SNPs must be included, and the optimization of the chip design is conditional on those obligatory SNPs. This is particularly important for the design of LD SNP chips for customers with applications that are specific to a population rather than a trait [22].

In this paper, we present an MOLO algorithm for optimizing the design of LD SNP chips. A heuristic, local-search algorithm was used to find the local optima, which approximate the global optimum, rather than maximizing the objective function analytically. The algorithm was implemented in an R package and was called selectSNP. A trial version of selectSNP (S1 File and S2 File) is available upon request to the corresponding author (nwu@neogen.com) and subject to signing an agreement for non-commercial use. Features of the MOLO algorithm were demonstrated through the design of ultra-LD (ULD) SNP chips under various constraints. For practical applications, the algorithm was used to design a ULD 5K SNP chip for dairy cattle and a common, multi-breed bovine 24K SNP chip. Imputation accuracy and prediction accuracy were assessed wherever applicable. Propagation of imputation errors into genomic prediction and utilization of LD SNP chips for genomic prediction and selection were also considered. Finally, the performance of imputation-mediated genomic prediction was described, in which three sets of 6K SNPs were impute to 80K genotypes in a U. S. Holstein population.

## Materials and Methods

### Objective Function

The MOLO algorithm centers on an objective function, *f(x)*, which maximizes the adjusted system information (Shannon entropy) and non-gap map length for a set of selected SNPs under multiple constraints (e.g., on MAFs, location distribution of SNPs, inclusion of obligatory SNPs, and number and size of gaps). That is,

max{f(x)|g(x),h(x),i(x|o),r}, (1)

where *g*(**x**) collectively includes all equality constraints, *h*(**x**) includes all inequality constraints, *i*(**x**|**o**) represents constraints given the set of obligatory SNPs, and 0 ≤ *r* ≤ 1 is a tunable parameter for the bin width that is used in the heuristic search for local optima.

The location distribution of SNPs can be initialized either uniformly or non-uniformly. In the latter case, an empirical Beta distribution is used to select SNPs according to their chromosomal locations, which leads to varied enrichment of terminal SNPs. Gaps are minimized given the number of SNPs on each chromosome. The SNP quality and fidelity criteria, such as call and Mendelian inconsistency rates, are resolved prior to optimization and hence are not included in the MOLO algorithm. Information for a chip can be computed based on the frequencies of either haplotypes or alleles.

The objective function in Eq (1) is highly non-linear if it can be mathematically expressed, and analytical solutions to this optimization problem may not always be available. Hence, a heuristic search algorithm is used to find local optima in an attempt to approximate the global optimum. Computing time is another issue that must be considered. With a large number of available SNPs, obtaining the global optimum is often not computationally possible.

### Distribution of SNPs across Multiple Chromosomes in the Genome

Consider a genome with *k* = 1, …, *K* chromosomes, each of length *L*_*k*_. Assume an abundant number of SNPs on each chromosome from which a set of *n*_*k*_ SNPs can be selected. Then the distribution of selected SNPs on those chromosomes follows a multinomial distribution in which the probability of having a specific number of SNPs on each chromosome is proportional to its map length:

$$\begin{array}{l} f\left(n_{1},\ldots ,n_{K};N,p_{1},\ldots ,p_{K}\right)=Pr\left(X_{1}=n_{1},\ldots ,X_{K}=n_{K}\right)\\ \;=\{\begin{array}{c} \frac{N!}{n_{1}!\ldots n_{k}!}p_{1}^{n_{1}}\ldots p_{K}^{n_{K}},\;when\;\sum_{k=1}^{K}n_{k}=N\\ 0\;Otherwise \end{array} \end{array}$$ (2)

Where

$$p_{k}=\frac{L_{k}}{\sum_{i=1}^{K}L_{k}}\times 100\%$$ (3)

Marginally, the distribution of SNPs on one specific chromosome, say *k*, is binomial:

$$\begin{array}{l} Pr\left(X_{1}=n_{1};p_{k},N\right)\\ \;=\{\begin{array}{l} \frac{N!}{n_{k}!\left(N-n_{k}\right)!}p_{k}^{n_{k}}{\left(1-p_{k}\right)}^{\left(N-n_{k}\right)},\;when\;0\leq n_{k}\leq N\\ \;0\;Otherwise \end{array} \end{array}$$ (4)

Hence, the number of SNPs on each chromosome, say *k*, can be empirically taken to be the expected value (mean):

$$E\left(X_{k}|p_{k},N\right)=N\times p_{k}=N\times \frac{L_{k}}{\sum_{i=1}^{K}L_{k}}$$ (5)

In reality, however, SNPs are not abundantly available on each chromosome, and gaps may exist in the physical or genetic maps, which adds complexity to the task of assigning SNPs to the chromosomes. Let there be *T*_*k*_ SNPs on chromosome *k*, from which *n*_*k*_ SNPs are to be selected. The mean distance between two neighboring selected SNPs is computed as

$${\bar{\Delta }}_{k}=\frac{L_{k}}{n_{k-1}}.$$ (6)

In our approach, a distance between two neighboring SNPs is identified as a gap on each of chromosome if it is more than twice as large as the average spacing distance. Hence, a gap is defined in a relative sense in this proposed method. When gaps are present, the map length of each chromosome needs to be adjusted because the gaps do not harbor any SNP. Now assume that the gaps on chromosome *k* account for *g*_*k*_ of its map length. The adjusted map length of this chromosome is (1−*g*_*k*_)*L*_*k*_. With this adjustment, the number of SNPs on each chromosome with gaps considered is a multinomial distribution that takes the same form as in Eq (1) but with the following adjusted probability:

$$p_{k}=\frac{(1-g_{k})L_{k}}{\sum_{i=1}^{K}(1-g_{k})L_{k}}$$ (7)

### Location Distribution of SNPs on Each Chromosome

**Fig 1:**
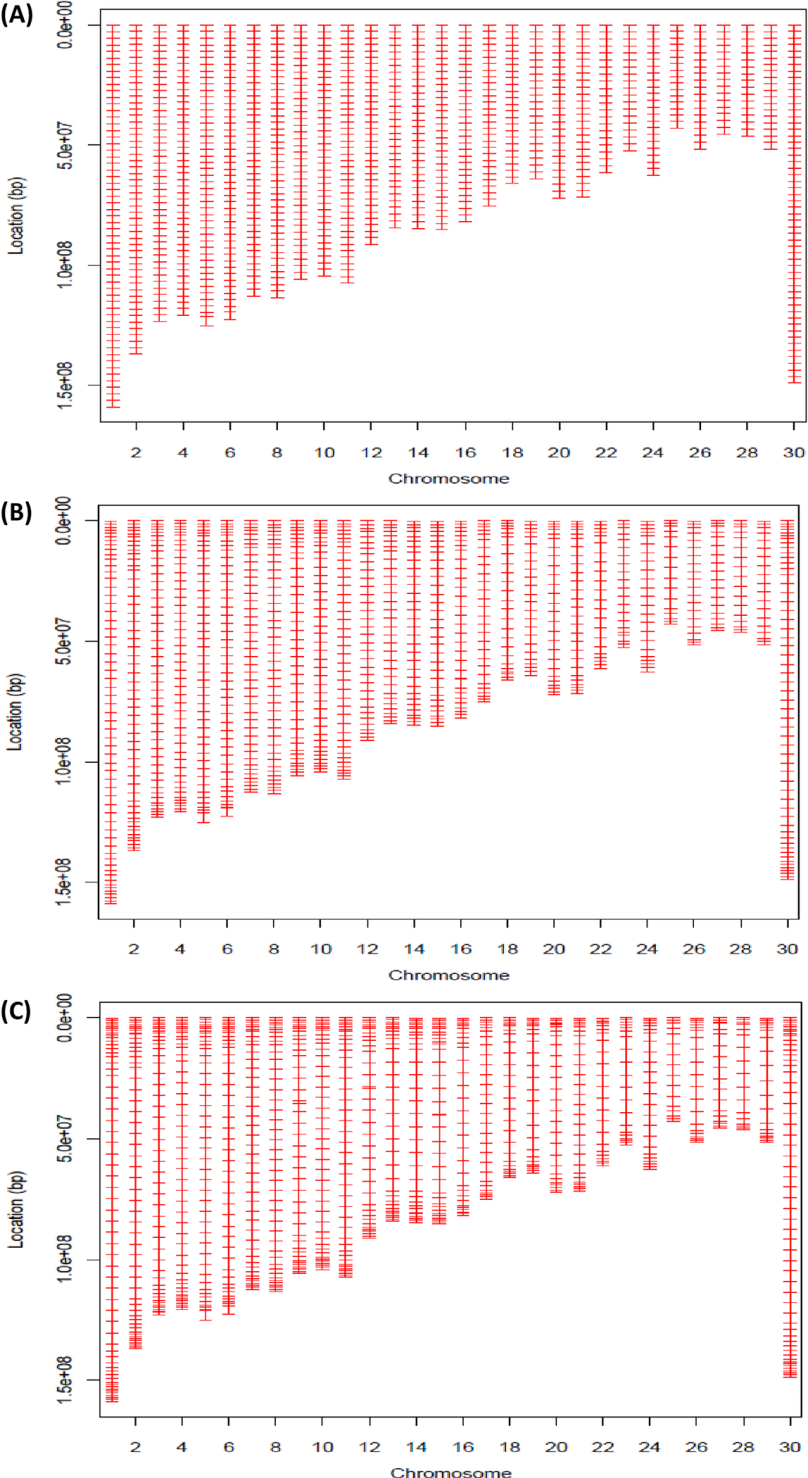
Illustration of three designs of ultra-low-density (ULD) SNP arrays. (A) In a uniform distribution, SNPs are selected on each chromosome with approximately even spacing. (B) In a non-uniform Beta-distribution design with “less” terminal enrichment, both ends of each chromosome have a slightly higher density of SNPs than in a uniform design. (C) In a non-uniform Beta-distribution design with “more” terminal enrichment, both ends of each chromosome have a greater density of VF SNPs than (B). A tuning parameter of γ = 0.05 was used for the radius of the neighborhood for the search with B and C. Of the three ULD SNP array, each had 1,000 selected SNPs.

After the number of SNPs (which includes obligatory SNPs) is determined for each chromosome, virtual-frame (VF) SNPs of the same number are placed on each chromosome regardless of whether or not a SNP is physically present at that location. The location distribution of the VF SNPs on each chromosome can be either uniform or non-uniform (Fig 1). The uniform design is simple and results in SNP maps that are approximately evenly spaced (Fig 1A). In practice, however, we found evidence of decreased imputation efficiency because of lack of flanking SNP information at both ends of each chromosome [16]. Therefore, non-uniform distributions of SNPs were also considered. The non-uniform design uses an empirically tunable Beta distribution to guide the location distribution of the VF SNPs on each chromosome, which allows for the enrichment of SNPs at both ends of each chromosome to varying extents (Fig 1B and 1C).

### Uniform Distribution of SNPs

Real maps can have gaps. Assume that there are *δ*_*k*_ gaps on chromosome *k* so that the chromosome length is divided into *s*_*k*_ = *δ*_*k*_ + 1 segments, each bounded by two gaps or located on either end of the chromosome. Then the distribution of SNPs assigned to these segments is governed similarly by a multinomial distribution which takes the same form as in Eq (1), and the probability of having a specific number of SNPs in each segment is proportional to the map length of the chromosomal segment without any gaps. In our method, the number of SNPs is initialized to be the lower boundary of the rounded mean for each segment, and unassigned SNPs may still remain. These unassigned SNPs are assigned to a set of chromosomes with a probability equal to the relative length of each chromosome.

Obviously, when no gaps exist, this method selects (approximately) evenly spaced SNPs on each chromosome segment. However, in the presence of gaps, the number of selected SNPs can differ dramatically for different chromosome segments, depending on the length of gaps on each segment. Hence, the number of selected SNPs may not necessarily be equal for each segment yet the selected SNPs are uniformly localized (or approximately so) in each segment.

### Non-Uniform SNP Distribution

**Fig 2:**
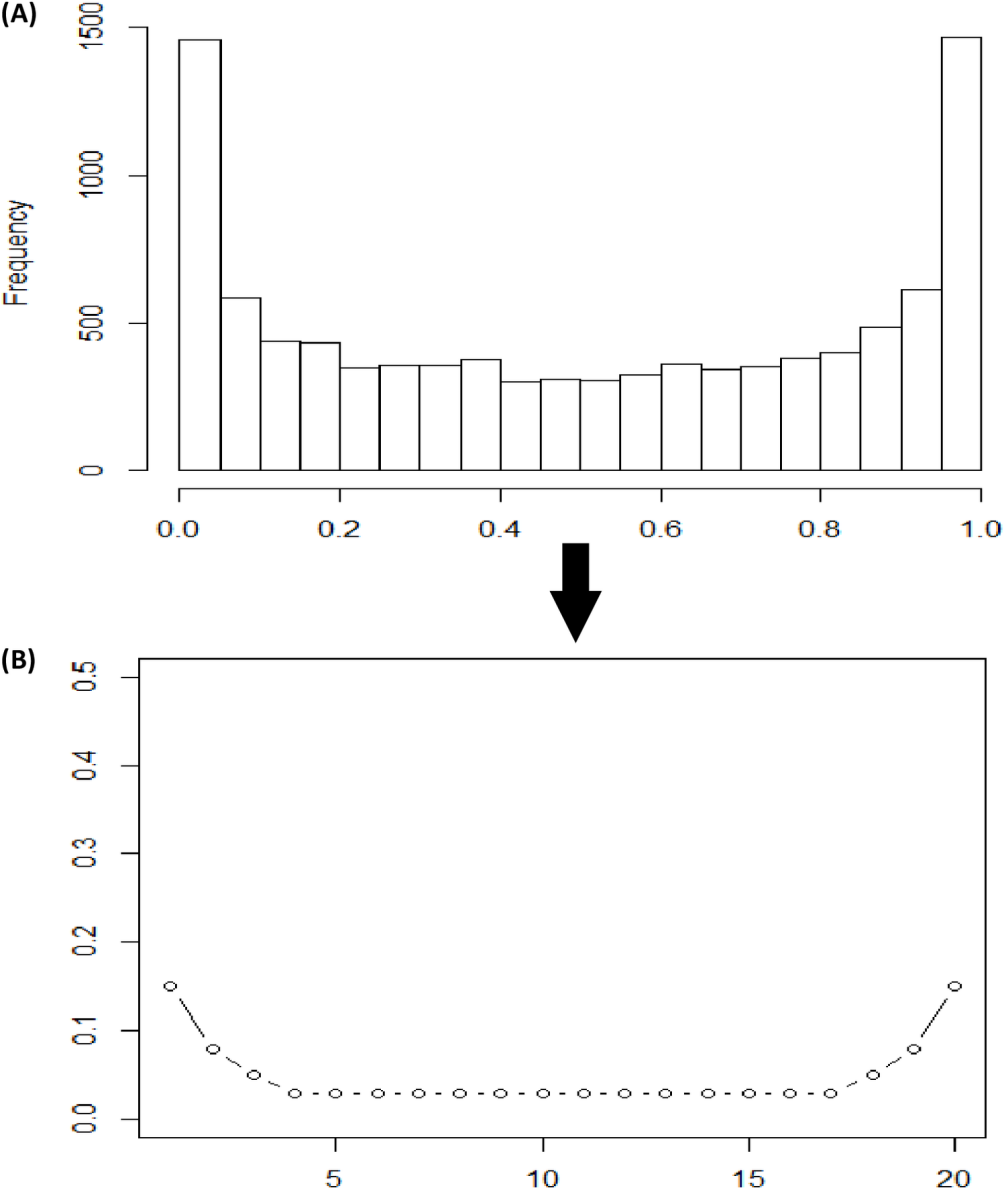
Empirically smoothed Beta distribution for guiding distribution of SNP locations on one chromosome. (A) The values of *X* (0 to 1) represent the proportional length of each chromosome. (B) The chromosome length is divided into twenty bins of approximately equal size, separated by twenty one frame SNPs, and SNPs are enriched in the outermost right bins and the outermost left bins.

For non-uniform chip designs, an empirical distribution obtained from smoothing the Beta(0.5, 0.5) distribution was used to guide the location distribution of the SNPs (Fig 2). This empirical distribution can be tuned to have varied extents of terminal bend-up, leading to different enrichments of SNPs in the terminal segments of each chromosome (Fig 1B and 1C).

For convenience, the selectSNP package implements two typical situations labeled as “more” versus “less” for SNP enrichment at both ends of each chromosome. Briefly, a chromosome is divided into 20 segments. Let the first two segments be the left-end group and the last two segments be the right-end group. Then, the middle group consists of the 16 interstitial segments. This leads to three meta-segments. When the “more” option is chosen, 29% of SNPs are allocated to the left- and right-end groups each; 42% are allocated to the middle group. When the “less” option is chosen, 22% of SNPs are allocated to the left- and right-end groups, with 56% to the middle group. Within each segment, the map length is further divided into sub-segments bounded by possible flanking gaps. Then the distribution of SNPs is governed by a multinomial distribution, which takes the same form as in Eq (1), with a probability of SNP allocation that is proportional to the block length of each segment.

### Map-Oriented vs. Information-Oriented Optimization

The map-oriented approach is to select evenly spaced SNPs based only on their map positions. Let the non-gap length of a chromosome be divided into equal bins (except that the first and last bins are both of half-bin length), and each non-terminal bin hosts only one SNP. The map-oriented approach always attempts to select SNPs that are exactly at or closest to the center of each non-terminal bin. Let **w** = {*w*_*j*_ | *j* = 1,…,*n*_*s*_} represent a set of *n*_*s*_ central locations for a chromosome segment of *n*_*s*_ bins, with each location representing a VF SNP, and **x** = {*x*_*s*_ | *i* = 1,…,*N*_*s*_} be a set of map positions for the *N*_*s*_ candidate SNPs located on that chromosome segment, from which a set of SNPs denoted by **z** = {*z*_*k*_ | *k* = 1,…,*n*_*s*_} is to be selected. Then the map-oriented approach always selects *z*_*k*_ = *x*_*j*_ as a match if

$$f(z_{j}|x,w)=\left\{x|\text{which.min}(|x-w_{j}|)\right\}\;\text{for}\;k=1,\ldots ,m_{s}.$$ (8)

where *which*.*min(A)* returns a minimum value in set A. In contrast, the proposed information-oriented optimization works on each chromosome segment and attempts to maximize the information (i.e., Shannon entropy) adjusted for the uniformness of the SNP distribution.

In information theory, entropy is the average amount of information contained in each message received. In the selection of SNPs, a message refers to an allele. For a bi-allelic locus, Shannon entropy *H* is computed as

$$H=\sum_{i=1}^{2}p_{i}\left[\log_{2}(p_{i})\right],$$ (9)

where *p*_*i*_ is the probability (frequency) of allele *i* at the observed locus. When *p*_*i*_ = (1−*p*_*i*_) = 0.50, *H* is maximized and equals 1. Therefore, the entropy for the selection of a single SNP with two alleles each at an equal frequency is 1 (which is also referred to as 1 bit). Now consider two SNPs for which there are four possible allele combinations (or haplotypes). If each of the four haplotypes is equally probable, $H=-\sum_{i=1}^{4}0.25\left[\log_{2}(0.25)\right]=2$, and the entropy for the selection of two SNPs each with equal allele frequencies is 2 bits. Without loss of generality, the selection of *m* SNPs (each with two equally probable alleles) is *m* bits. For two or more SNPs, *H* ranges from 0 to the number of SNPs.

For multiple SNPs, *H* can be computed for each SNP and then summed and averaged across all SNPs. The average is referred to as locus-average Shannon entropy (${\bar{H}}_{\text{L}}$), which ranges from 0 to 1:

$${\bar{H}}_{\text{L}}=-\frac{\sum_{j=1}^{m}\sum_{i=1}^{2}p_{i}\left[\log_{2}(p_{i})\right]}{m}.$$ (10)

Alternatively, a haplotype-average Shannon entropy (${\bar{H}}_{\text{H}}$) can be computed for *m* SNPs jointly and then divided by the total number of all possible haplotypes (observed and not observed)

$${\bar{H}}_{\text{L}}=-\frac{\sum_{i=1}^{I}p_{i}\left[\log_{2}(p_{i})\right]}{l},$$ (11)

where *l* = 2^*m*^; ${\bar{H}}_{\text{H}}$ also ranges from 0 to 1. Note that ${\bar{H}}_{\text{H}}$ is comparable only for the same number of SNPs, as is the case for ${\bar{H}}_{\text{L}}$.

In the MOLO algorithm, average *H* is adjusted for the uniformity of the SNP distribution. Consider *m* SNPs located on a chromosome segment, and the *m* SNPs divide this chromosome segment into *m*−1 intervals, each flanked by two neighboring SNPs. Denote interval *j*, with *j* = 1,…,*m*−1, as *δ*_*j*_. The adjusted average *H*, denoted by ${\bar{H}}_{\text{adj}}$, is

$${\bar{H}}_{adj}=\bar{H}\times \left(1-\frac{\frac{1}{m-1}\sum_{j=1}^{m-1}\left|\delta_{j}-\bar{\delta }\right|}{c\times \bar{\delta }}\right)$$ (12)

where $\bar{\delta }=\left(\frac{1}{m-1}\right)\sum_{j=1}^{m-1}\delta_{j}$ is the average SNP spacing distance, and *c* ≥ 1 is a constant that empirically tunes the weights. The larger *c*, the smaller weights and the less adjustment that will be made. Obviously, ${\bar{H}}_{\text{adj}}=\bar{H}$ when $\delta_{1}=\ldots =\delta_{m-1}=\bar{\delta }$. On a single-SNP basis, maximizing *H* is equivalent to maximizing MAF, whereas maximizing *H*_adj_ involves weighting based on the uniformity of the SNP distribution.

**Fig 3:**
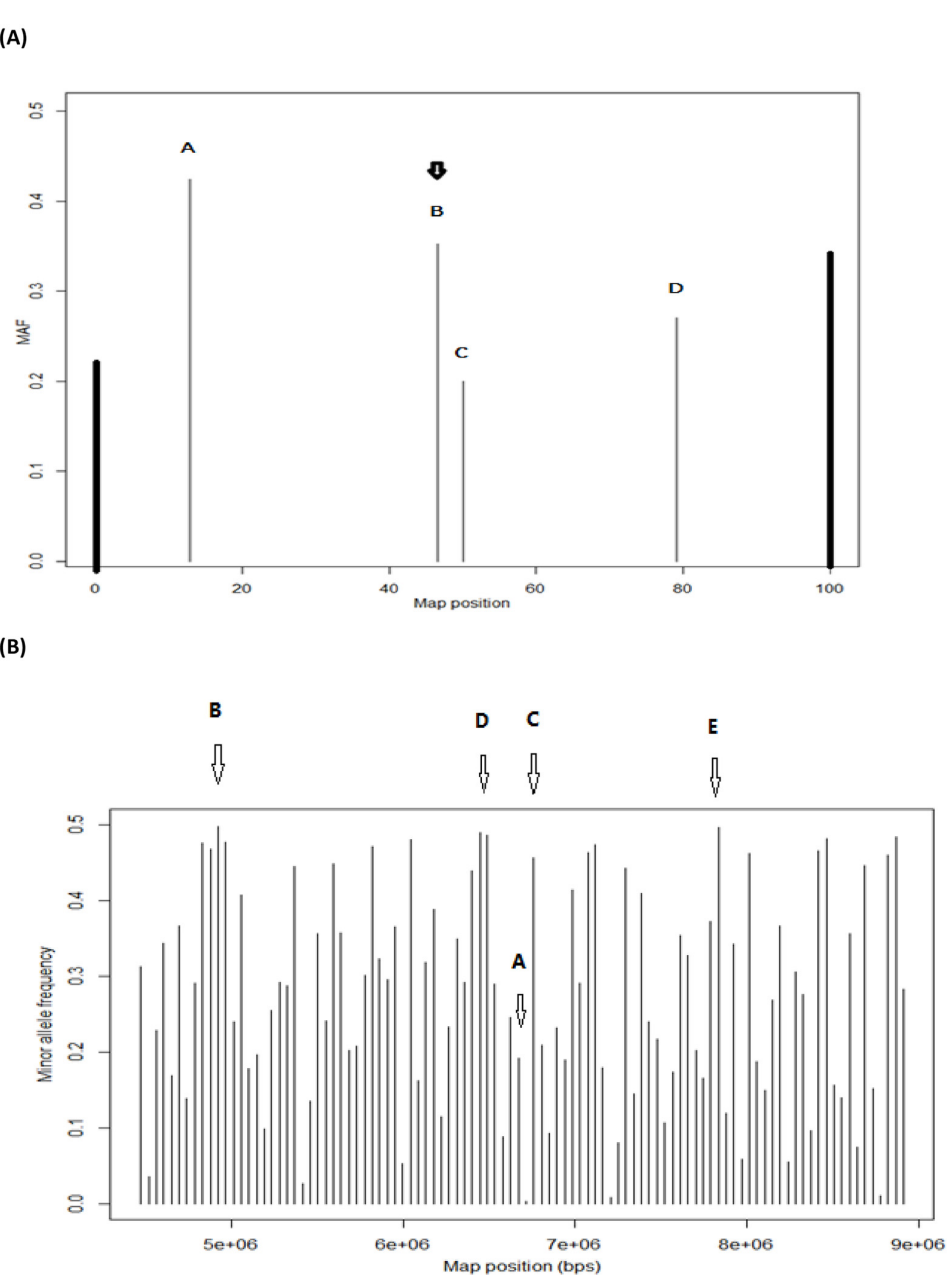
Four SNPs on an arbitrary chromosome segment that are candidates for a SNP array. (A) Four candidate SNPs are denoted as A, B, C, and D; two boundary SNPs are represented by two bold vertical lines at positions 0 and 100. The virtual-frame SNP at the center of the chromosome segment is not shown; (B) Selecting one SNPs out of 100 SNPs located between.

To illustrate how the MOLO algorithm works, consider selecting one SNP from four candidate SNPs (A, B, C, and D) on an arbitrary chromosome segment of length 100 (Fig 3A). A VF SNP is located at the center of this chromosome segment (not shown). The MAFs of the four candidate SNPs are 0.4239 (A), 0.3524 (B), 0.2000 (C), and 0.1708 (D), respectively, and their *H* values are 0.9832, 0.9373, 0.7219, and 0.6596. The selection can be made in a number of different ways. The MAF-based approach selected SNP A because it had the highest MAF. This, however, led to a very uneven location distribution of SNPs. For obtaining evenly-spaced SNPs, SNP C was selected, but this SNP happened to be the least informative. The MOLO algorithm based on ${\bar{H}}_{\text{adj}}$ selected SNP B because it was the locally optimal candidate if considering both information and location. In this example, each of the four candidate SNPs divided the chromosome into two segments flanked by the two terminal SNPs, and their ${\bar{H}}_{\text{adj}}$ are 0.6181 (A), 0.9038 (B), 0.7219 (C), and 0.5991 (D), respectively. Although SNP B deviated slightly from the central position, it had a low decrease in information (<5% decrease in ${\bar{H}}_{\text{L}}$, as compared with choosing the SNP with the highest MAF). A more realistic example is shown in Fig 3B, in which 100 SNPs are located from 4476100 bps to 8907439 bps on one chromosome. MAF for these SNPs varies from 0.003828 (51th SNP) to 0.497512 (11th SNP). When the optimization was made on map position solely, it selected SNP A (50th SNP) because it was the most centrally located but it had a very low Shannon entropy. The 11th SNP (B) had the greatest Shannon entropy and SNP selection optimizing LASE (c = 1) picked this SNP. Next, the adjust Shannon entropy by the positional distribution of SNPs with various weighting for c = 1, 100, 100,000, and 1,000,000, respectively. With c = 1, SNP C (52nd SNP) was selected, which was moderately highly informative and also approximately centrally located. Increasing the value of c to 100 and 100,000, respectively, led the algorithm to choose more informative SNPs (D and E, which are the 48th and 76th SNP, respectively) yet with a large deviation from the central location. With a very high value for c (i.e., c = 1,000,000), the algorithm has little effect on weighting for location-wise SNP distribution and it selected the same SNP (B) as the optimization on LASE.

Hence, selecting SNPs based on adjusted information compromised between the system information content and SNP distribution, yet it does not necessarily decrease system information in reality. To the contrary, when a set of obligatory SNPs is pre-included, selecting SNPs based on MAF alone can be very inefficient and can lead to very unevenly spaced SNP panels which is often sub-optimal for imputation. Nevertheless, this situation can be handled well by the MOLO algorithm.

Often, selecting SNPs based on ${\bar{H}}_{\text{H}}$ provides the same result as that based on ${\bar{H}}_{\text{L}}$, but the frequencies of haplotypes for three SNPs (two fixed boundary SNPs and one candidate) need to be computed. For simplicity, let all haplotypes be equally probable. Then, a local search selects SNP B plus the two boundary SNPs if this set of three SNPs yields the greatest adjusted ${\bar{H}}_{\text{H}}$.

### Heuristic Local Optimization

One of the notable features of the proposed method is that the objective function is maximized empirically through a local optimization search rather than being optimized analytically. A heuristic local optimization algorithm is proposed, which defines local regions on the whole genome and then attempts to select a subset of SNPs with locally maximized ${\bar{H}}_{\text{adj}}$.

Two methods of searching for local optima are considered: (1) maximizing ${\bar{H}}_{\text{L}}$ within a neighborhood defined by the tuning parameter (*γ*) or (2) maximizing ${\bar{H}}_{\text{H}}$ over a number of consecutive bins, both of which can be adjusted for the uniformness of the location-wise SNP distribution. The first approach examines each SNP one at a time. Consider a chromosome segment without gaps on which *m* optimal SNPs are to be selected. Then *m* VF SNPs are marked, which divides this chromosome segment into *m*−1 bins; each bin will host only one SNP. Let $\bar{\Delta }$ be the average SNP spacing distance computed for the number of SNPs to be selected. To select SNPs one at a time, the algorithm searches locally within a radius (*γ* × Δ) from each interval center (i.e., the location of a VF SNP), where 0 ≤ *γ* ≤ 0.5. Define $isLocal(x)=(abs(x-w_{j})\leq \gamma \times \bar{\Delta_{s}})$. Then the heuristic local search always attempts to find a candidate *z* = *x*_*j*_ ∈ ***x*** such that it satisfies:

$$f(z_{j}=x_{j}|x,\gamma )=\{x|which.max(adjusted\_\bar{H}(Shannon;z_{j});isLocal==TRUE)\}$$

The second approach approach maximizes adjusted ${\bar{H}}_{\text{H}}$ by considering several SNPs in neighboring bins, with one SNP taken from each bin. For example, if three consecutive bins are considered with *m*_1_, *m*_2_, and *m*_3_ SNPs, respectively, in each bin, the three selected SNPs will have up to 2^3^ = 8 possible haplotypes. If all combinations of these three-SNP haplotypes are examined, this requires *m*_1_ × *m*_2_ × *m*_3_ rounds of local searches. Hence, the optimization based on haplotypes can be highly computationally demanding, if each bin contains a large number of SNPs. The heuristic local search attempts to find a candidate ***z*** = ***x***_***s***_ ∈ ***x*** such that it satisfies

$$\begin{array}{l} f(z|x,\gamma )=\{x|which.max(adjusted\_\bar{H}(Shannon;z);isLocal==TRUE)\} \end{array}$$

where $adjusted\_\bar{H}(Shannon;z)$ is the adjusted HASE computed for a set of SNPs denoted by **z**.

### Design of Ultra-Low-Density (ULD) Bovine SNP Chips

This part consisted of two independent researches. In the first research, the features of the MOLO algorithm were illustrated through the design of ULD bovine SNP chips (1K, 3K, and 5K). Issues of interest included the impact of the values of the tuning parameter γ, initial location distributions of SNPs (uniform versus non-uniform), and ULD chip sizes (1K, 3K, and 5K). In the second, conditional optimization was demonstrated with the presence of 1K and 3K obligatory SNPs in 5K-SNP chips.

**Fig 4:**
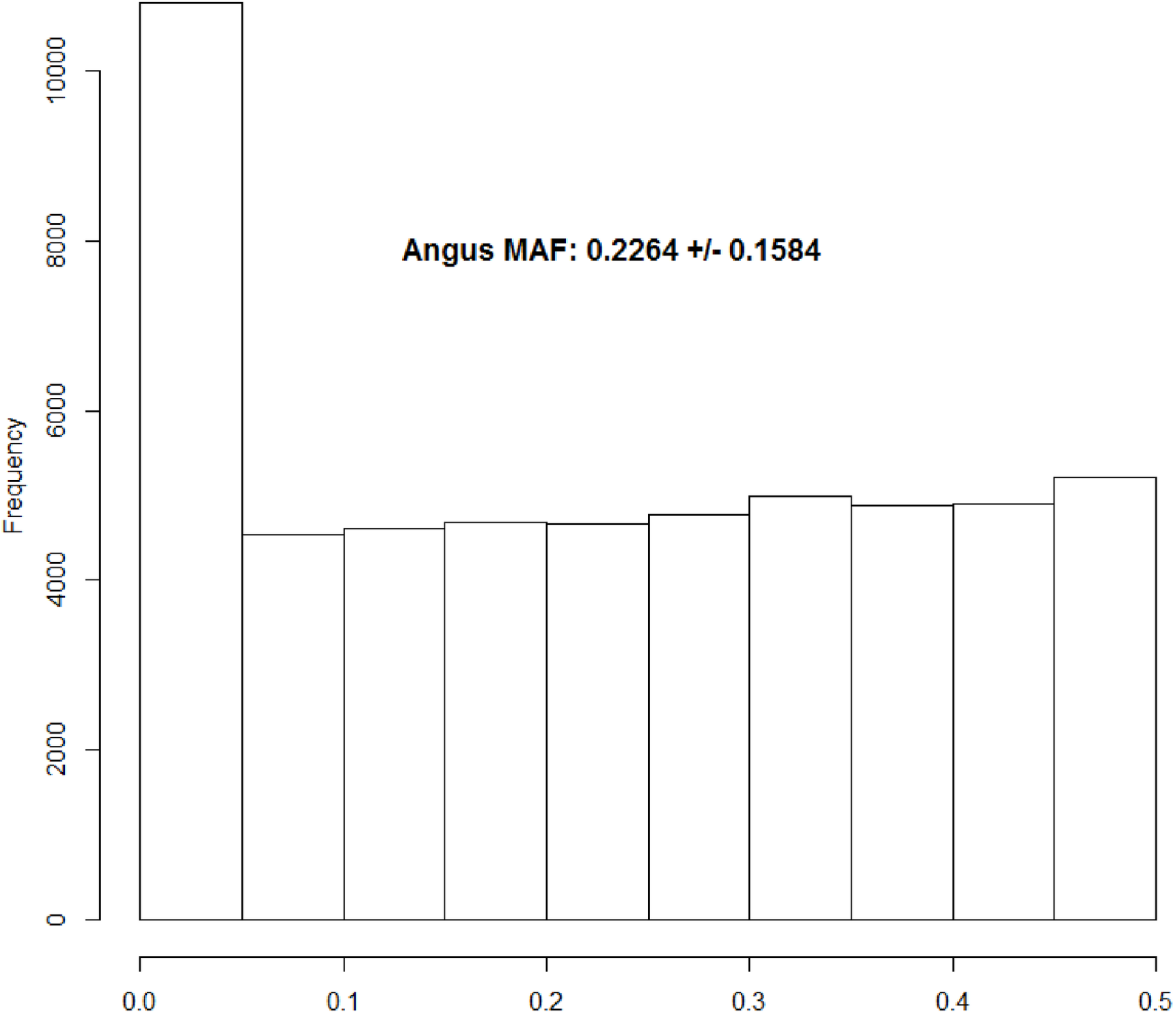
Histogram showing minor allelic frequencies (MAF) for the 50K assay in the Angus population. The mean (and standard deviation) of the MAF for the 50K SNP genotypes for this Angus population was 0.2264 (0.1584).

To investigate the impact of the tuning parameter γ on the optimal design of ULD panels, optimization was conducted with a grid of values (0, 0.1, 0.2, 0.3, 0.4, 0.5, 0.6, 0.7, 0.8, 0.9, 1) assigned to the tuning parameter, evaluated one at time, for each of the three SNP chip sizes. The accuracy of imputation to 50K genotypes was then evaluated in an Angus population genotyped by each ULD panel. The MAF distribution is shown in Fig 4. For validation, 10 data sets, each consisting of 400 animals, were randomly sampled and used to compute the imputation accuracy for each experiment.

Next, three Dairy 5K SNP chips were designed to conditional optimization in the presence of a set of obligatory SNPs. The first Dairy 5K SNP chip was optimized without the inclusion of any obligatory SNPs. The second Dairy SNP chip was optimized conditional on the pre-inclusion of 1,099 (1K) obligatory SNPs (including 121 parentage SNPs and 98 breed determining SNPs), while the third Dairy 5K SNP chip was optimized conditional on the pre-inclusion of 2,641 (3K) obligatory SNPs (the previous 1,099 obligatory SNPs plus 2,589 SNPs from the historical 3K assay). Mean MAF for the 50K SNP genotypes was 0.2802 in a Holstein population used as the reference population. Independent validation of the imputation accuracy for the Dairy 5K SNP chips was conducted by a USDA team.

### Design of a Common, Multi-Breed, 24K Bovine SNP Chip

In this application, a common, optimal 24K SNP chip for use in 7 cattle breeds (Gelbvieh, Angus, Simental, Charolais, Hereford, Limousin, and Holstein) was designed. Of these breeds, Holstein is a dairy breed and the remaining are beef breeds. Gelbviehs were originally bred to be triple purpose cattle (milk, beef, and draught), but the modern Gelbvieh is primarily used for beef production (http://www.gelbvieh.org/). The 24K bovine SNP chip design included a list of 7K (7,569) obligatory SNPs.

The 24K SNPs were selected from the BovineSNP50 SNP map, which has 54,609 SNPs spanning the bovine autosomal genome and X chromosome (http://www.illumina.com/Documents/products/datasheets/datasheet_bovine_snp5O.pdf). SNPs with incomplete or missing map or MAF data were excluded. After data editing, there were in total 52,038 SNPs remaining for Angus and 54,056 SNPs for each of the five other remaining beef breeds. The average MAF for the seven cattle breeds ranged from 0.2189 (Angus) to 0.2802 (Holstein) and the average Shannon entropy ranged from 0.6265 (Angus) to 0.7760 (Holstein). The correlations between breeds for MAF and locus-averaged Shannon entropy based on the BovineSNP50 genotypes varied considerably among the seven cattle populations: the MAF correlation was the lowest (22.16% to 39.63%) between the dairy Holstein and each of the beef cattle breeds (Table 1), but was relatively higher among the six beef breeds, from 50.46% between Hereford and Limousin to 80.46% between Gelbvieh and Simmental.

**Table 1:** Correlations between minor allelic frequency (MAF, upper triangle) and between locus-average Shannon entropy (LASE, lower triangle) for the seven cattle populations^a^.

	Gelbvieh	Angus	Simmental	Charolais	Hereford	Limousin	Holstein
**Gelbvieh**	0.2264	66.61%	80.46%	75.32%	52.57%	76.79%	39.63%
	0.6484						
**Angus**	76.50%	0.2189	69.04%	55.93%	38.89%	62.36%	28.27%
		0.6265					
**Simmental**	88.12%	79.02%	0.2223	72.76%	50.74%	75.79%	36.67%
			0.6395				
**Charolais**	83.07%	65.71%	81.23%	0.2229	53.51%	74.22%	38.79%
				0.6402			
**Hereford**	63.86%	48.50%	62.15%	64.68%	0.2233	50.46%	22.16%
					0.6346		
**Limousin**	85.31%	73.22%	84.86%	82.55%	61.99%	0.2204	37.70%
						0.6343	
**Holstein**	41.20%	30.95%	38.79%	40.73%	24.66%	39.29%	0.2802
							0.7760

^a^ Numbers in each of the diagonal cells are average minor allelic frequencies and locus-average Shannon entropy, computed for each of the seven cattle populations respectively.

Based on these MAF correlations, our strategy was to design a common chip with a shared core but also including SNP subsets that were specific to each breed. Hence, the R script running on the selectSNP package consecutively implemented the following:

- Include 7K obligatory SNPs as the base set;
- Add 6K SNPs as the backbone, optimized given the list of the obligatory SNPs and a MAF threshold (MAF > 0.10);
- Include the Dairy 5K SNPs (DULD-C), which were previously optimized using the MOLO algorithm;
- Include 4K SNPs optimized for Angus using the MOLO algorithm, conditional on the base set and the backbone SNPs;
- Include 3K SNPs optimized for each of the remaining beef cattle breeds (Gelbvieh, Simmental, Charolais, Hereford, and Limousin) using the MOLO algorithm, conditional on the base set and backbone SNPs;
- After pooling the selected SNP subsets, a final optimization step was conducted to adjust SNPs by their map locations and to fill SNPs in large gaps as was necessary.

Imputation error rate from 24K to 50K genotypes was estimated in an Angus beef cattle population of 3,894 animals. Imputation of 24K genotypes to 50K genotypes was conducted using the Beagle program [23].

Propagation of imputation errors to genomic prediction was investigated in 3,894 Angus (beef) animals and 2,639 Holstein (dairy) animals, respectively. In the Angus population, the accuracy of genomic prediction was computed for 21 traits when predicted using original 50K SNP genotypes and imputed 50K SNP genotypes from 1K, 3K and 5K LD genotypes, whereas in the Holstein population the accuracy of genomic prediction using original 80K genotypes and imputed 80K genotypes from three sets of 6K SNP genotypes was computed for three traits. Genomic prediction equations were built previously for Angus [24] and Holstein (unpublished), respectively. Genomic prediction accuracy was measured to be correlation of the estimated genetic values (e.g., EPD for Angus and PTA for Holstein) and GEBV using the original 50K genotypes or the 50K genotypes imputed from the ULD genotypes in the validation sets.

### Genomic Prediction Using Imputed 80K Genotypes in U.S. Holsteins

The last part was a real application study, in which the selectSNP package was used to improve the performance of 6K LD SNPs for imputation-mediated genomic prediction in U.S. Holstein animals. The training population consisted of 7,012 animals, each genotyped by the GGP (GeneSeek Genomic Profile) HD 80K chip (77,376 SNPs). Prior to the data analyses, SNPs on chromosome 0, MT and Y were all excluded. After data cleaning, a summary of 76,694 bovine SNPs on this GGP HD SNP chip was shown S2 Table. The “phenotypes” included predicted transmitting abilities (PTA) for daughter pregnancy rate (PDR), milk yield (Milk) and fat yield (Fat), respectively, which had been estimated for these Holstein animals using BLUP (best linear unbiased prediction). The summary statistics of PTA for the three trait are listed in S3 Table. To build genomic prediction equations, SNP effects were estimated by regression PTA on 68,748 SNPs with > 5% minor allele frequency in with < 5% minor allele frequency was removed as well, which retained 68,748 SNPs in this training set of 7,012 Holstein animals.

Three sets of 6K LD SNPs were selected using different strategies. First, 2,000 SNPs with the largest SNP variance for each of the three traits were selected. Let *p*_*i*_ and *q*_*i*_ be frequency of the two alleles of the *j*-th SNP, and *α*_*j*(*k*)_ be the corresponding (additive) association effects that this SNP had on the *k*-th trait. The SNP variance pertaining to this specific one is given by:

$$v_{j\left(k\right)}=2p_{i}q_{i}\alpha_{j\left(k\right)}^{2}$$ (13)

Next, the three 2K trait-specific SNP panels were pooled into a common LD SNP panel for the three traits, leading to a panel of 5,260 unique SNPs (denoted by 6KA). Furthermore, 740 SNPs were selected by the selectSNP package, optimized by the LOMO algorithm, and added to the 6KA panel to make an amendment of 6K LD panel (denoted by 6KB). The third LD SNP panel consisted of 6,000 with the largest average standardized SNP variances. For each SNP, the standardized SNP variance was computed as the following:

$$v_{j\left(k\right)}=\frac{1}{w_{\left(k\right)}}2p_{i}q_{i}\alpha_{j\left(k\right)}^{2}$$ (14)

where $w_{\left(k\right)}=\sum_{j=1}^{p}2p_{i}q_{i}\alpha_{j(k)}^{2}$ and *p* is the total number of SNP. Hence, the 6KC panel included 6,000 SNPs with the largest average of the standardized SNP variances, computed as follows:

$$\bar{v_{j}}=\frac{1}{3}\sum_{k=1}^{3}\left(\frac{1}{w_{\left(k\right)}}2p_{i}q_{i}\alpha_{j\left(k\right)}^{2}\right)$$ (15)

Imputation accuracy from 6K genotypes to 80K genotypes and genomic prediction accuracy using imputed 80K genotypes were evaluated in the testing dataset of 2,639 Holstein animals. Imputation accuracy was taken to be the percentage of correctly imputed SNP genotypes in non-reference SNPs (i.e., SNPs that assumedly had all missing genotypes). The genomic-estimated breeding values (GEBV) of an animal was calculated to be a sum of all SNP effects for that animal. Genomic prediction accuracy was evaluated in term of correlation between GEBV and PTA for all animals in the validation set. Note that these genomic prediction accuracies were considered to be approximate, because PTAs were not the true total (additive) genetic variance but were obtained with errors. Nevertheless, genomic prediction accuracy computed this way served well the purpose for validation of genomic prediction models in the present study.

## Results

### Factors Affecting Optimization Using the MOLO Algorithm

#### The tuning parameter

The tuning parameter *γ* defines the radius of the neighborhood for the search for each local optimum. For example, consider a chromosome or a chromosome segment of length *L*, on which *m-2* SNPs are to be selected, in addition to the two boundary SNP which are always included. Then, adding *m-2* VF SNPs uniformly on this chromosome or chromosome segment generated *m*-1 bins, with the average bin length being equal to *L/(m-1)*. The tuning parameter 0 ≤ *γ* ≤ 0.5 defines the search range in the neighborhood on both sides of each VF SNP, and the search returns either an obligatory SNP or SNPs, if existent, or an optimal SNP with the greatest information adjusted for its map location. Letting γ = 0.5 covers half of the whole search distance centered on each VF SNP. When each VF SNP is individually examined and every local search covers half the distance on both sides of each VF SNP, the chromosome will be covered approximately entirely. Hence, γ = 0.5 is an empirically critical value that yields a roughly 100% coverage in the search for local optima. In practice, though, it is possible to have γ > 0.5, it introduces over-lapping in the search and the information of the resulting system tends to decrease. On the other hand, with γ = 0, local optimization does not occur. Instead, the algorithm selects whichever SNP is located exactly at the VF SNP or the one that is closest in position to the VF SNP. This tuning parameter, however, is not relevant when the optimization is based on HASE. In the latter case, the increment of system information accelerates with the number of VF SNPs being looked at together (which defines local haplotypes).

Our results show that, during the local optimization search, the system information tended to increase considerably as the value for γ increases from 0 to 0.5, and that it plateaued thereafter (Table 2). This happened because with γ > 0.50 it introduced a partial over-lap in the local optimum search for two neighboring VF SNP and, as a consequence, the relative gain in system information tended to decrease. Consider the 3K ULD SNP chips as examples. LASE was between 0.7881 and 0.7895 with γ = 0 but increased to > 0.97 when γ = 0.50, and plateaued afterwards. Relative to the uniform design with γ = 0 (which served as a uniform design control, based only on map position), the trend of increment actually slowed down when γ = 0.30~0.50 (Table 2).

**Table 2:** Impact of the tuning parameter γ on locus-average Shannon entropy as calculated based on allele frequencies (3K SNP panel).

Model	Tuning parameter γ	Average Shannon entropy	Increment% over Unif-00 ^a^
**Uniform**	0	0.7881	0.00%
**Uniform**	0.1	0.8939	13.42%
**Uniform**	0.2	0.9415	19.46%
**Uniform**	0.3	0.9627	22.15%
**Uniform**	0.4	0.9715	23.27%
**Uniform**	0.5	0.9768	23.94%
**Uniform**	0.6	0.9787	24.18%
**Uniform**	0.7	0.9804	24.40%
**Uniform**	0.8	0.9816	24.55%
**Uniform**	0.9	0.982	24.60%
**Uniform**	1	0.9828	24.70%
**Beta**	0	0.7895	0.18%
**Beta**	0.1	0.8963	13.73%
**Beta**	0.2	0.9465	20.10%
**Beta**	0.3	0.9663	22.61%
**Beta**	0.4	0.9764	23.89%
**Beta**	0.5	0.9816	24.55%
**Beta**	0.6	0.9837	24.82%
**Beta**	0.7	0.9853	25.02%
**Beta**	0.8	0.9873	25.28%
**Beta**	0.9	0.9877	25.33%
**Beta**	1	0.9883	25.40%
**Random**	0	0.6337	-19.59%

^a^ Unif-00 = Uniform design with γ = 0.

**Fig 5:**
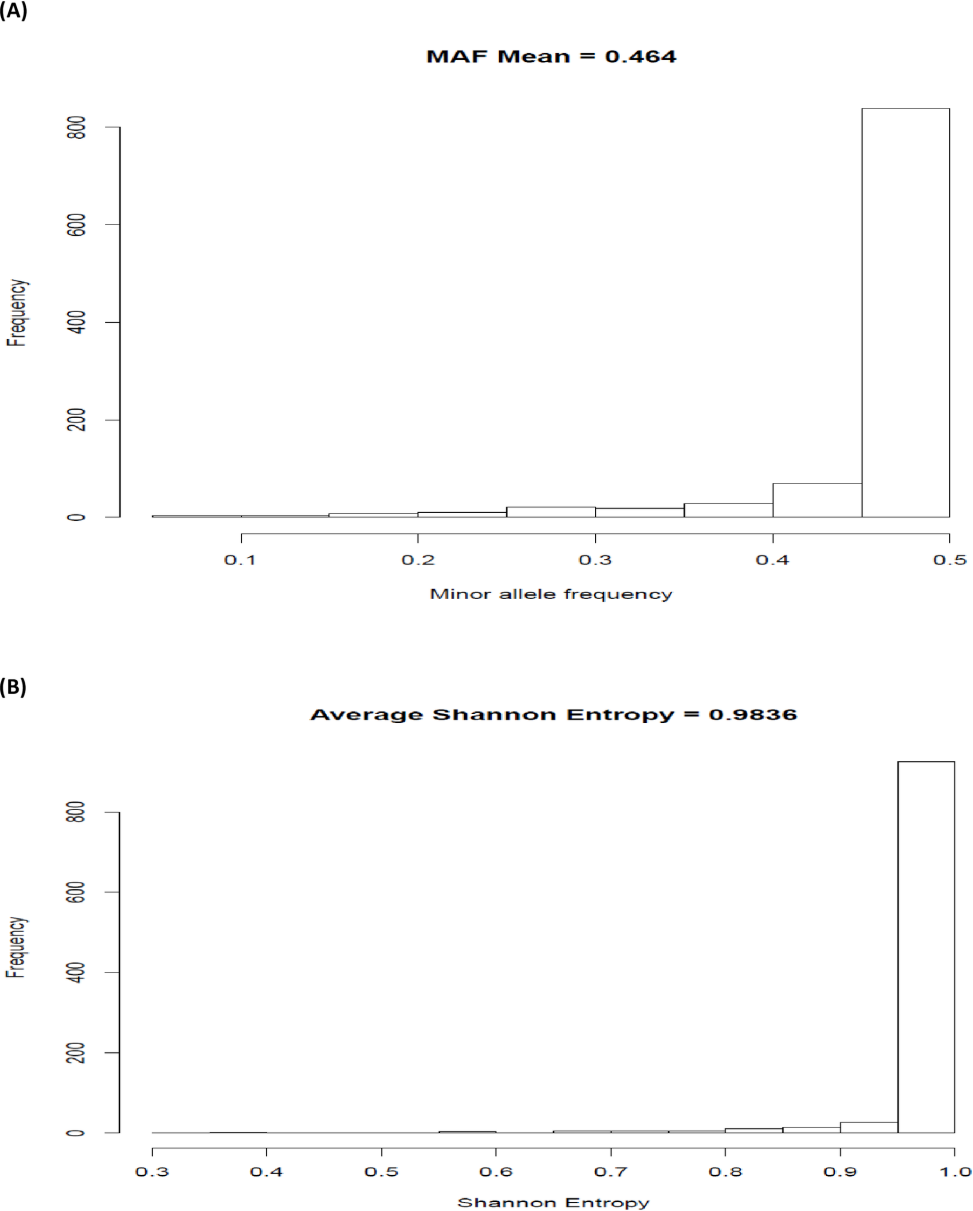
**Histograms of (A) minor allele frequencies (MAF) and (B) locus-average Shannon entropy (LASE) for an Angus population, computed for 3K SNP genotypes.**

Evidently, increased system information (LASE) was achieved through increased minor allelic frequencies. In the example discussed above, the MOLO algorithm effectively elevated the MAF of selected SNPs from 0.2264 (Fig 4) to 0.4640 (Fig 5A) and, subsequently, LASE was increased from 0.7807 to 0.9836 (Fig 5B).

**Fig 6:**
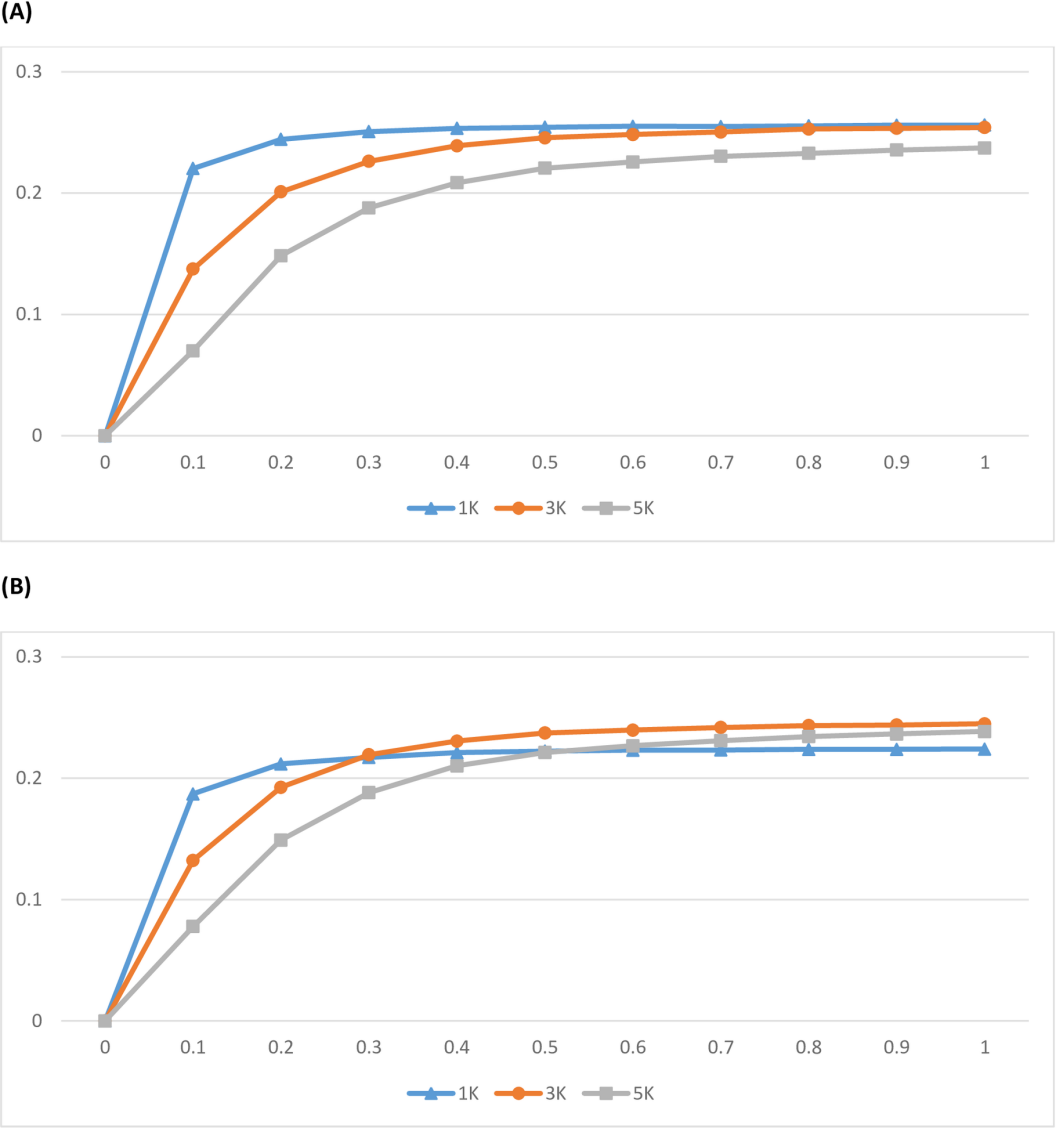
**Increment of Locus-Average Shannon Entropy (LASE) relative to a control chip designed based solely on SNP position (3K SNP chips) with a uniform design (A) versus a Beta design (B).** The X-axis represents the turning parameter *γ*, which took values between 0 and 1, and the y-axis represents increment of LASE for each ULD panel (1K, 3K, and 5K, respectively) relative to the control (*γ* = 0).

Relative to the evenly-spaced chip design of the same size as the control (Uniform with γ = 0), the percent increment was larger for the 1K SNP chip than for the 3K or 5K SNP chips, and the trends were similar regardless of SNP location distributions, either uniform or Beta (Fig 6).

As previously mentioned, both LASE and HASE are comparable only among chips with same number of SNPs. Nevertheless, these results suggest that the tuning parameter γ has a greater impact on the optimization of chips with lower SNP density: the lower SNP density, the greater impact of the tuning parameter on the optimization. Often, the LASE increment tended to plateau with γ ≥ 0.40. For the uniform ULD chips (1K, 3K, and 5K), the relative increment in LASE was between 7.78% and 18.70% when γ = 0.10, 18.80% and 21.70% when γ = 0.30, 22.11% and 23.72% when γ = 0.50, and 22.39% and 24.48% when γ = 1.

#### ULD chip size, information criteria, and types of SNP distribution

Imputation accuracy also varied with the size (i.e., the number of SNPs) present on a LD chip, and the information criteria used for the optimization of the design. Imputation accuracy was evaluated for three ULD chip sizes: 1K, 3K and 5K, when these ULD genotypes were imputed to 50K SNP genotypes. For each ULD chip size, imputation accuracy was computed for 10 data subsets, each consisting of 400 randomly selected animals. Three evenly-spaced chips of the same sizes (Uniform γ = 0) were also evaluated as the controls (Unif-00). The chips that were optimized using the MOLO algorithm produced higher imputation accuracies than did the controls, and the imputation accuracy was higher when imputation was made on more (e.g., 5K) reference SNPs (5K) than fewer (e.g., 1K) SNPs (Table 3). Optimization based on HASE led to better imputation accuracies than that based on LASE, but the difference diminished as the chip size reached 5K. Relative to the controls, the increment in imputation accuracy was 8.2 to 8.6% (LASE) and 19.5 to 25.2% (HASE) for 1K genotypes, 3.4 to 3.9% (LASE) and 7.4 to 7.8% (HASE) for 3K genotypes, and 2.6 to 3.4% (LASE) and 3.6 to 3.8% (HASE) for 5K genotypes. On the absolute scale, imputation accuracy was 81.87 to 82.17% (LASE) and 90.43 to 95.69% (HASE) when imputed from 1K genotypes, 95.25 to 95.73% (LASE) and 98.94 to 99.32% (HASE) when imputed from 3K genotypes, and 98.66% to 99.40% (LASE) and 99.62 to 99.82% (HASE) when imputed from 5K genotypes (Table 3). For the controls, the imputation accuracies resulting from the evenly spaced chip designs were 75.65, 92.13, and 96.15% when imputing from 1K, 3K, and 5K genotypes, respectively. We also observed that imputation accuracy increased as the haplotype included more SNPs (5 versus 3 SNPs), when imputing from 1K and 3K genotypes, but the difference became small when imputing from 5K genotypes. It should be noted that the more SNPs included in the haplotypes, the more computing time was required.

**Table 3:** Comparison of imputation accuracy (standard error) for 1K, 3K and 5K panels, obtained using various of design optimization strategies.

	Unif-00 ^a^	Unif-50 ^b^	Beta-50 ^c^	Halp-3B ^d^	Halp-5B ^e^
**1K**	75.65% (2.36%)	81.87% (2.35%)	82.17% (2.27%)	90.42% (1.84%)	94.69% (1.80%)
**3K**	92.13% (0.96%)	95.25% (0.86%)	95.73% (0.84%)	98.94% (0.60%)	99.32% (0.60%)
**5K**	96.15% (0.57%)	98.66% (0.56%)	99.40% (0.58)	99.62% (0.45%)	99.82% (0.45%)

^a^ Unif-00 = SNPs were selected based on (uniform) map location only

^b^ Unif-50 = SNPs were initialized uniformly and selected based on locus-average Shannon Entropy within local search ranges defined by γ = 0.5

^c^ Beta-50 = SNPs were initialized according to an empirical Beta distribution and selected based on locus-average Shannon Entropy within local search ranges defined by γ = 0.5

^d^ Halp-3B = SNPs were selected based on average Shannon Entropy, computed for 3 SNP haplotypes with each SNP from one of 3 consecutive bins.

^e^ Halp–5B = SNPs were selected based on average Shannon Entropy, computed for 5 SNP haplotypes with each SNP from one of 5 consecutive bins.

The ULD chips from the Beta-design slightly outperformed those from the Uniform-design in terms of their information (Table 2) and imputation accuracy (Table 3), but this trend was not universal, and we also observed the opposite in other populations (data not presented). In essence, whether or not a Beta-designed chip outperforms a uniform-designed chip depends on the contrast of information among the terminal SNPs versus those located in the middle chromosome segments. As was often observed, enriching the numbers of informative SNPs on both ends of the chromosome tended to increase the frequencies of observed crossovers and hence led to higher imputation accuracies.

### Conditional Optimization of Dairy 5K ULD SNP Chips

In commercial applications, a list of obligatory SNPs may be relevant and they must be included in the chip design prior to selection of the remaining SNPs. This was exactly the case in designing the Dairy 5K SNP chips, in which the chip maps were pre-occupied by either 1K (1,099) or 3K (2,641) obligatory SNPs. Hence, the optimization was conducted in the presence of these obligatory SNPs. The Dairy 5K ULD SNP chips followed either uniform- or Beta- designs. The tuning parameter was set to be *γ* = 0.25 because the preliminary results showed that the increase in information slowed significantly when larger tuning parameter values were used. The optimal 5K panel “D5K-pre1K” included 3,750 SNPs optimally selected conditional on the presence of 1,099 obligatory SNPs, plus 151 SNP slots reserved for 100 Bacterial SNPs, 9 Y chromosome SNPs, 3×12 bin C-type SNPs, 6×1 bin A-type SNPs, in order to meet the Illumina requirement for manufacturing this chip. Note that, on Illumina, markers are binned according to the number and type of beads needed for a working assay. A bin A- or bin B-type SNP uses 2 beads to deal with ambiguous bases (A/T or C/G) where the same dye is used for each base. A bin C-type SNP uses 1 bead for assays that use two different dyes. Similarly, the 5K panel “D5K_pre3K” included 2,099 SNPs optimally selected conditional on 2,641 obligatory SNPs (which included the previous 1,099 obligatory SNPs), plus 258 SNP slots reserved for 100 Bacterial SNPs, 9 Y chromosome SNPs, 3×12 bin C-type SNPs, 6×1 bin A-type SNPs, and 107 SNP with no map information.

Compared to the evenly spaced SNP chip as the control, the MOLO algorithm increased the system information (LASE) by 15.91% to 17.02% for the optimal Dairy 5K chips with 1K obligatory SNPs and by 7.92% to 8.81% for the Dairy 5K chips with 3K obligatory SNPs, the increment of LASE was 24.26% and 25.15% when no obligatory SNPs were present (Table 4). Hence, given the same number of total SNPs, including more obligatory SNPs led to a decrease in the system information. Though the 3K obligatory SNPs had a slightly higher MAF than did the 1K obligatory SNPs (data not presented), the Dairy 5K chip with 3K obligatory SNPs had a lower LASE, because pre-including 3K obligatory SNPs left fewer slots for the optimal selection of the remaining SNPs than the 5K chip with 1K or no obligatory SNPs. This explained why the system information decreased when obligatory SNPs were included. These data again showed that the Beta-distributed SNP chips were more informative than the chips designed using uniformly distributed SNPs, but the differences are very slight.

**Table 4:** Comparison of locus-average Shannon entropy (LASE) for Dairy 5K SNP chips optimized with (1K and 3K) or without obligatory SNPs.

	Uniform Model			Beta Model		
	U00 ^d^	U25 ^e^	Increment% ^h^	B00 ^f^	B25 ^g^	Increment% ^h^
**pre0K** ^a^	0.7683	0.9615	25.15%	0.7691	0.9557	24.26%
**pre1K** ^b^	0.8139	0.9524	17.02%	0.819	0.9493	15.91%
**pre3K** ^c^	0.8272	0.9001	8.81%	0.8298	0.8955	7.92%

^a^ pre0K = Dairy 5K SNP chips with no obligatory SNPs

^b^ pre1K = Dairy 5K SNP chips with 1,099 obligatory SNPs

^c^ pre3K = Dairy 5K SNP chips with 2,641 obligatory SNPs.

^d^ U00 = Uniform-distributed 5K SNP chip optimized only on map position

^e^ U25 = Uniform-distributed 5K SNP chip optimized using the MOLO algorithm (*γ* = 0.25)

^f^ B00 = Beta-distributed 5K SNP chip optimized only on map position

^g^ B25 = Beta-distributed 5K SNP chip optimized using the MOLO algorithm (*γ* = 0.25)

^h^ Increment% = percent increment of locus-average Shannon entropy (LASE) of the Dairy 5K SNP chips optimized using the MOLO algorithm (U25 or B25) over the corresponding control optimized only on map positions (U00 or B00).

**Fig 7:**
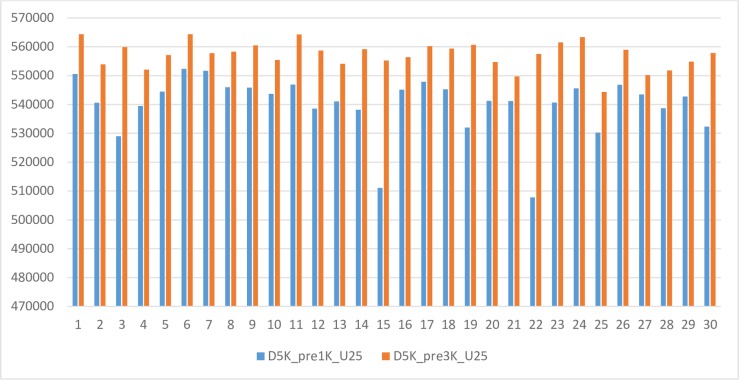
Comparison of SNP spacing between two Dairy 5K SNP chips. The X-axis represents chromosomes, where 30 = chromosome X. SNP spacing is measured as the square root of the average sum of squares of all the distances between two SNPs.

The Dairy 5K SNP chip with 3K obligatory SNPs also had a larger SNP spacing than its counterpart with 1K obligatory SNPs (Fig 7). Here, we measured SNP spacing as the square root of the average sum of squares of all distances between neighboring pairs of SNPs on each chromosome. With a large number of obligatory SNPs, it becomes extremely difficult to design a chip with “evenly-spaced” SNPs, and the efficiency of optimization can be decreased considerably. The Dairy 5K SNP chip with 1K obligatory SNPs (optimized by the MOLO algorithm with *γ* = 0.25) has a SNP spacing of 540,010 base pairs (± 10,161 bp), with a range from 507,788 to 552,342 bp. The Dairy 5K SNP chip with 3K obligatory SNPs (optimized by the MOLO algorithm with *γ* = 0.25) has an SNP spacing of 557,218 ± 4,654 bp, with a range from 544,340 to 564,357 bp. Hence, the SNP spacing for the chip with 3K obligatory SNPs was 3.19% larger than that for the chip with 1K obligatory SNPs. In general, pre-inclusion of a number of SNPs tends to decrease the system information, unless this pre-included subset itself is highly informative relative to the chip design criteria. Otherwise, the higher portion of obligatory SNPs, the lower efficiency that the optimization algorithm can achieve.

The MOLO algorithm was compared to two existing approaches: 1) uniform design (UD) characterized by evenly-spaced SNPs, and 2) optimization on maximized MAF (OMM). UD is the simplest approach and selects evenly-spaced SNPs on each chromosome (Habier et al., 2009). To maximize the map length, the algorithm started with the first SNP and ended at the last SNP, and then selected SNPs evenly on each chromosome. The OMM algorithm selected SNPs with the greatest MAF on each given segment of chromosomes. Somewhat differently, the MOLO algorithm selected SNPs by locally maximizing the information, which was also adjusted for the distribution and uniformity of SNP locations on each chromosome. In the MOLO algorithm, the local bin was defined conditionally on the presence of a set of obligatory SNPs and gaps, which was often ignored by the OMM algorithm.

**Fig 8:**
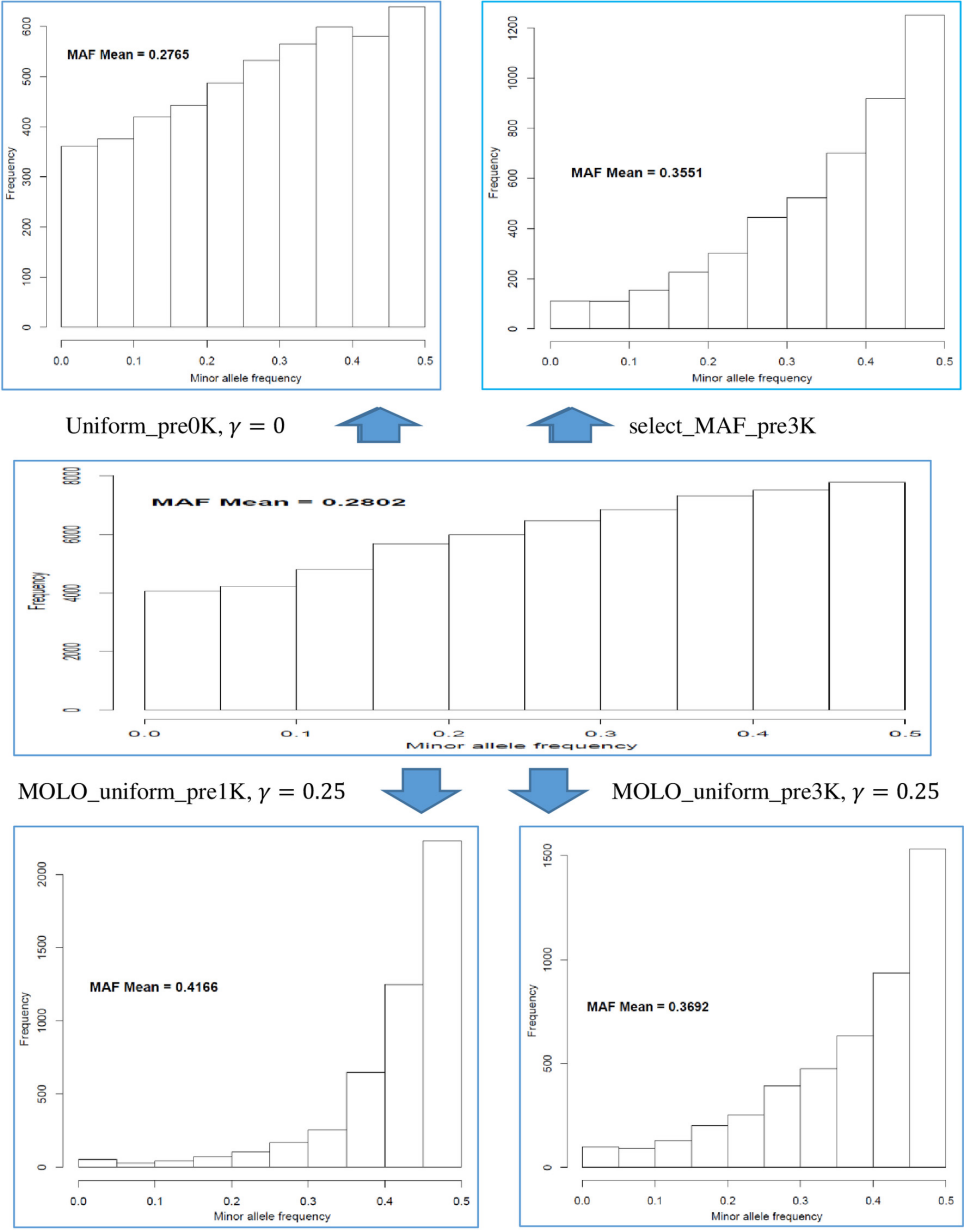
Illustration of the distributions of minor allele frequency (MAF) in response to the use of different strategies to optimize the design of Dairy 5K SNP chips. In the upper left is the histogram of MAF for the uniform chip and in the upper right is the histogram of MAF for the chip optimized solely on MAF. The MAF distribution for the reference Holstein population, prior to SNP selection, is shown in the middle graph. The MOLO algorithm considerably increased MAF for the two 5K optimal panel, as shown in the lower two graphs.

**Fig 9:**
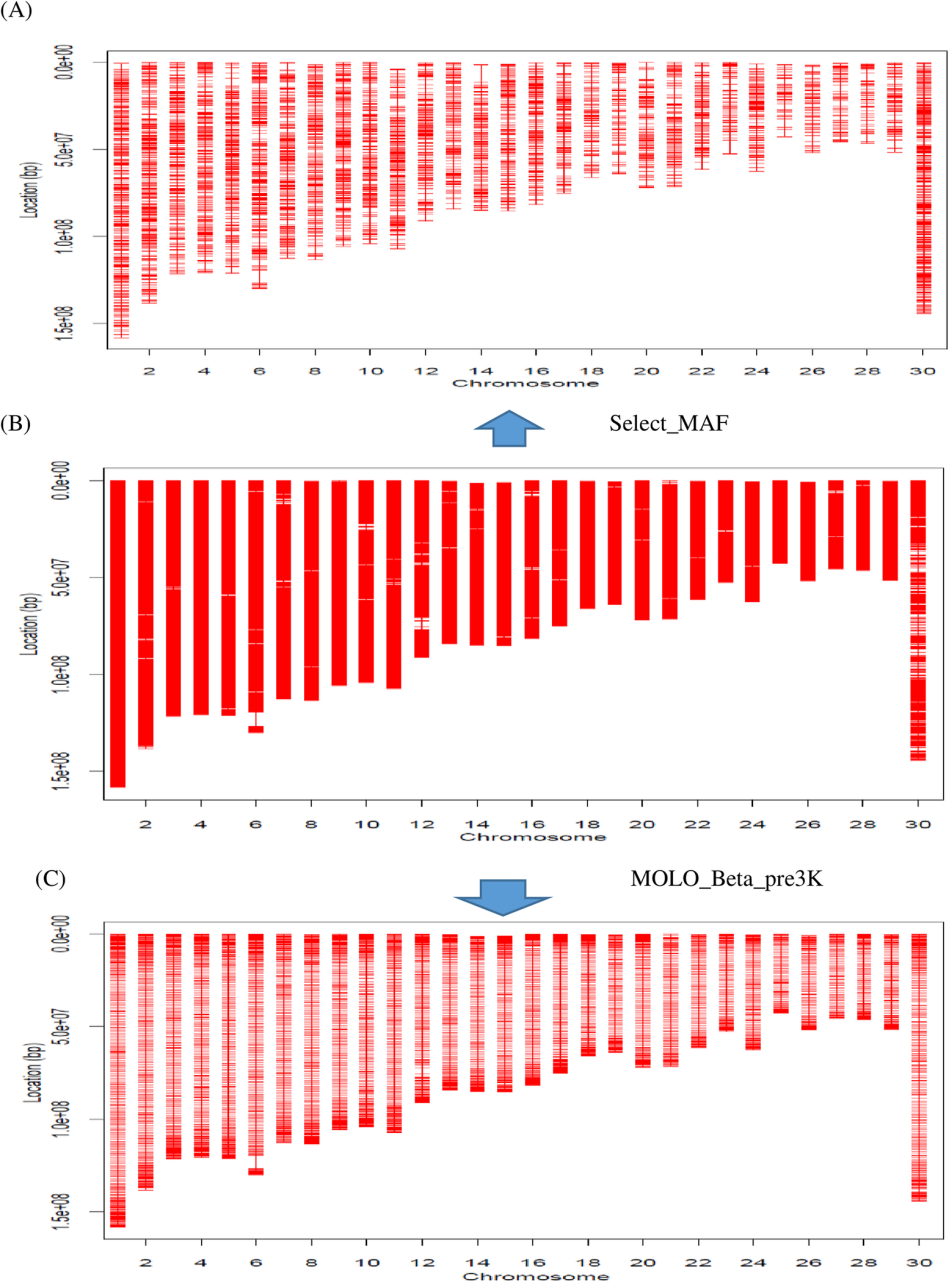
**Map view of the Dairy 60K SNP map (B) and the two 5K SNP maps (A and C).** The two 5K SNP maps were optimized on minor allele frequency (A) and using the MOLO algorithm (C), respectively. The X-axis represents chromosomes, where 30 = chromosome X.

The MOLO algorithm was also more accurate in locating a set of (approximately) evenly-spaced and high informative SNPs than both the UD algorithm. As shown in Fig 8, the UD had the smallest average Shannon entropy. This is because the UD method selected a set of evenly-spaced SNPs based on their map positions, and, as long as SNPs of high MAF did not coincided with these selected SNPs, it did alter MAF for the selected SNPs (Fig 8, upper left graph). Hence, the average MAF was comparable to that of the whole candidate set with only some slight difference in this example. The OMM method substantially increased MAF (Fig 8, upper right graph) and the system information, but the location distribution of selected SNP was very uneven (Fig 9, upper graph). In contrast, the MOLO algorithm not only maximized MAF (and hence the system information) as one of its goals (Fig 8, lower graphs), it also selected a set of approximately evenly-spaced SNPs (Fig 9, lower graph).

In reality, selecting evenly distributed SNPs with the highest MAFs can be extremely complex when about > 50% of the SNPs had already been assigned as obligatory SNPs, as is the case with D5K-pre3K, and selected SNPs can deviate considerably from being “uniformly-distributed”. To handle this situation, the MOLO algorithm adjusts the information criterion for the distribution and uniformity of SNP locations within the map, thus leading to a more even-spaced SNP chip design.

In this dairy cattle example, there were still a few gaps that were not filled, e.g., on chromosomes 6 and 12, in Fig 9 (lower graph), which were actually inherited from a customer 60K chip map that was also included in the pool of SNP available for selection. Because there were no SNPs available to choose from in these regions, these gaps remained unfilled. Besides, the system information (LASE) for the Dairy 5K SNP chips obtained using the MOLO algorithm was larger than those optimized with the MAF-based method. This confirms that the MOLO algorithm can also generate a more informative chip than the method that simply maximizes MAF alone, because the latter algorithm is very inefficient when > 50% SNPs need to be pre-included. In the design of the Dairy 5K SNP chips, the optimization operated on LASE, not HASE, because the computing time was many times longer than that with the former, yet both results were highly comparable for ULD SNP chips of 5K SNPs or more.

The mean imputation error ± SE over the 709 animals was 1.47±0.57%, with a range from 0.56% to 12.74% on an individual sample basis. This is equivalent to saying that the mean imputation accuracy was 98.53%, with a range of from 87.25% to 99.44% when the imputation accuracy was computed on an individual sample basis.

### Optimization of a Common Variant 24K Bovine SNP Chip

#### An optimal, common variant 24K bovine SNP chip

A multiple-breed, 24K bovine SNP chip comprising common variants was designed using the MOLO algorithm. The optimization using the selectSNP package output a list of 24,003 SNP, of which 259 obligatory SNPs do not have map data. The map statistics for the 24K (23,744) in comparison to those for the BovineSNP50 (54,056 SNPs) chip are summarized in S1 Table.

Briefly, this common variant, multiple-breed assay consists of 7,569 SNPs in the base set, 6,000 SNPs as the backbone, 4,847 SNPs from the previously optimized Dairy SNP chip, 4,000 SNP optimized for Angus, 3,000 SNPs for each of the remaining five beef cattle breeds, and 259 reserved slots for obligatory SNPs without map data (S1 Fig). The number of SNPs on each chromosome ranged from 389 SNPs (chromosome 25) to 1,398 SNPs (chromosome 1), with the middle 50% two quantiles being between 580 SNPs and 985 SNPs per chromosome. The total map length was 2.667 Gbp. The map length was the shortest for chromosome 25 (42.825 Mbp) and the longest for chromosome 1 (158.199 Mbp). In comparison, the BovineSNP50 chip has 54,056 SNPs on the 30 chromosomes (chromosomes 1–29 and chromosome X), covering a total map length of 2.651 Gbp.

**Fig 10:**
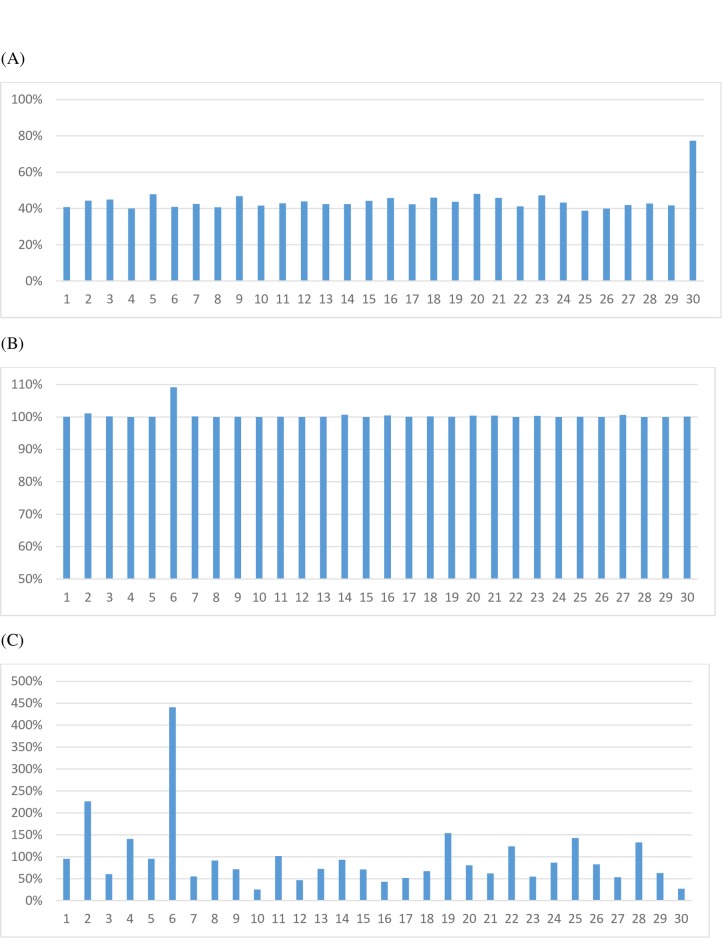
**Number of SNPs (A), map length (B), and maximum gap (C) on each chromosome for the 24K SNP chip, all expressed as a percentage of its counterpart on the 50K bovine SNP chip.** The X-axis represents chromosomes, where 30 = chromosome X.

**Fig 11:**
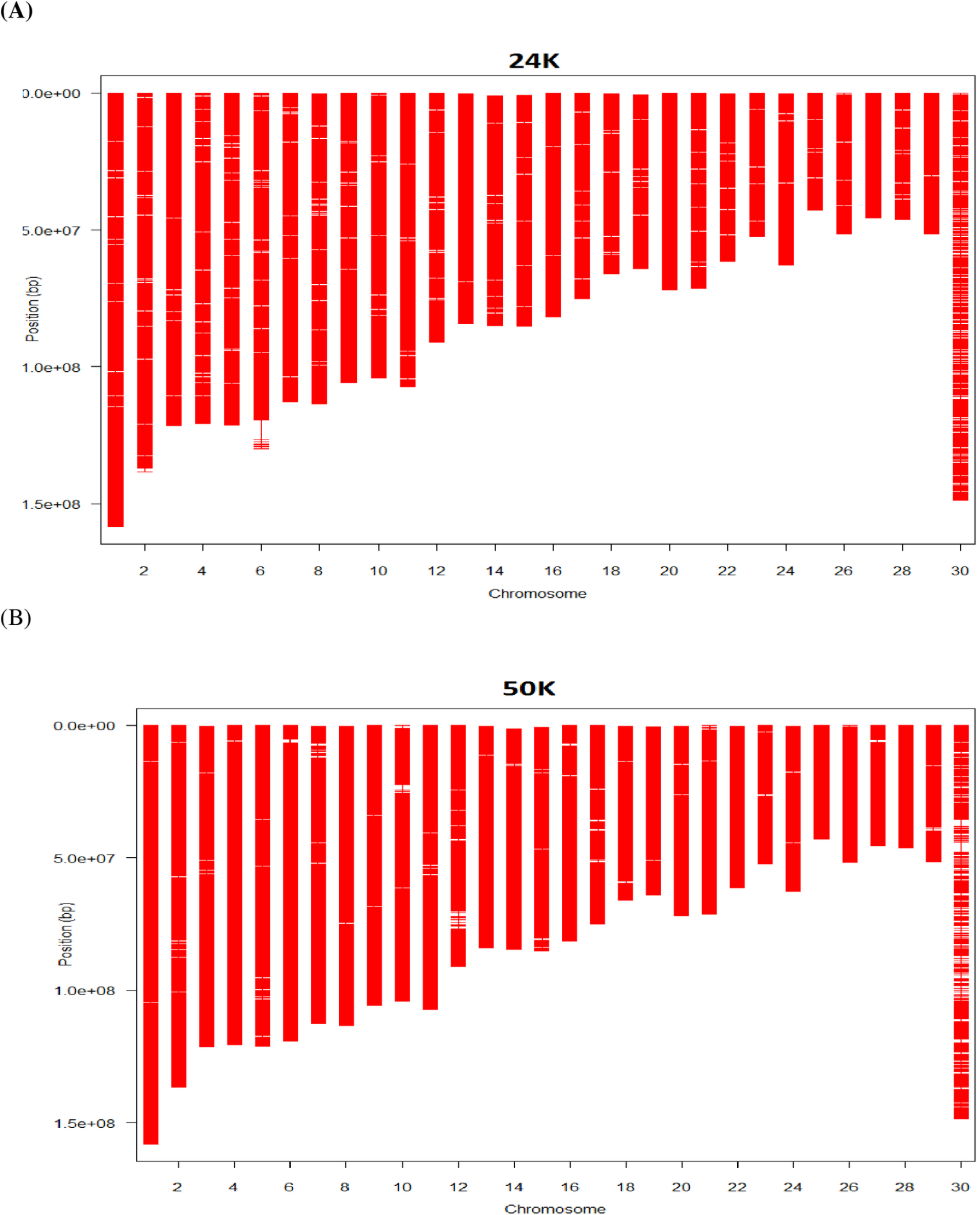
**Map view of the 24K (A) and 50K (B) bovine SNP chips.** The X-axis represents chromosomes, where 30 = X chromosome.

Chromosome-wise, the total number of SNPs on the 24K bovine SNP chip was, on average, 44.37% as many as that on the BovineSNP50, with a range from 38.75% (chromosome 25) to 77.35% (chromosome X) (Fig 10A). If ignoring chromosome X, all chromosomes on the 24K SNP chip had <50% as many SNPs as their counterparts on the BovineSNP50 chip, with a range from 38.75% (chromosome 25) to 48.02% (chromosome 20). Though the BovineSNP50 chip had more than twice the number of SNPs than the 24K bovine chip, the chip maps were mostly of the same lengths for both chips (Fig 10B). This is because the MOLO algorithm maximized the map length as one of its goals. Nevertheless, for 22 of the 30 chromosomes in the design of the 24K chip, the maximum gap on each chromosome was smaller than that found on the BovineSNP50 chip (Fig 10C). Chromosome 6 is a special case. Its map length for the 24K chip was approximately 9% (~11 Mbp) longer than that for the BovineSNP50 chip, but the number of SNPs on this extra chromosomal segment for the 24K SNP chip was few. Because the LOMO algorithm maximized both the information and the map length for each chromosome, it included this chromosome segment despite of the existence of a large gap there. Apart from this gap, the maps for the 24K bovine SNP chip were better covered by the SNPs than are the maps for the BovineSNP50 chip (Fig 11).

#### Accuracy of imputation from 24K to 50K genotypes

Imputation accuracy was evaluated in random validation sets for the seven cattle populations, with each validation set consisting of between 293 and 500 animals (**Table 5**). For each breed, the imputation accuracy was computed as the average across 10 random replicates. The average imputation accuracy for the seven breeds ranged from 99.21 (Simmental) to 99.94% (Holstein) (**Table 5**). The imputation accuracy was the highest for the Holsteins, possibly there were the most number of Holstein-specific SNPs, and because its validation size was the largest. Also possibly, the Holstein population might have the highest homogeneity as well, as compared to these beef breeds.

**Table 5:** Validation of imputation accuracy in seven cattle breeds.

Breed	N ^a^	Number of SNPs		Imputation accuracy% ^b^		Range %	
		24K ^c^	50K ^d^	Mean	Std ^e^	Min ^f^	Max ^g^
**Angus**	389/3,894	23,207	55,074	99.49	0.05	99.46	99.59
**Charolais**	408/1,028	21,344	54,056	99.32	0.08	98.83	99.71
**Gelvieh**	266/1,065	21,344	54,056	99.31	0.08	98.77	99.72
**Hereford**	323/3,233	21,344	54,056	99.63	0.06	99.38	99.81
**Limousin**	293/2,930	21,344	54,056	99.23	0.07	99.04	99.38
**Simmental**	412/4,114	21,344	54,056	99.21	0.07	98.97	99.46
**Holstein**	500/22,084	21,636	54,046	99.94	0.02	99.93	99.95

^a^ N = Number of animals randomly sampled for validation of imputation accuracy/total number of animals with 50K genotypes in each breed, from which a random validation set is sampled

^b^ Imputation accuracies was presented as averages across 10 replicates.

^c^ 24K = Number of SNPs in the 24K bovine chip (24,003 SNPs) that were present on the 50K bovine SNP chip

^d^ 50K = Number or SNPs on the 50K bovine chip as the target size for the imputation

^e^ Std = Standard deviation of imputation accuracy for each breed

^f^ Min = Minimum value of imputation accuracy for each breed

^g^ Max = Maximum value of imputation accuracy for each breed.

#### Genomic prediction using imputed 50K genotypes from ULD genotypes

A data set of 3,894 Angus animals was used to evaluate the accuracy of genomic prediction using 50K genotypes imputed from 1K, 3K, and 5K LD genotypes, respectively. The prediction accuracy using imputed genotypes was compared to that obtained using the original 50K genotypes. The average imputation error rates were 4.3, 0.18 and 0.09% for the 1K, 3K, and 5K ULD chips, respectively. The average ± SD of prediction accuracy computed for 21 traits was 81.54 ± 6.18% using the original 50K genotypes and 80.54 ± 6.63%, 81.40 ± 6.29%, 81.42 ± 6.29% using 50K genotypes imputed from 1K, 3K, 5K genotypes, respective (**Table 6**). The prediction accuracy in this Angus population was lower than we have previously obtained in another Angus population, because this Angus population is distantly related in time to the training population which had been used for developing genomic prediction equations (Okut et al., 2013).

**Table 6:** Prediction accuracy using the original 50K genotypes and the imputed 50K genotypes assessed in 3,894 Angus animals.

Trait ^a^	Prediction accuracy % ^b^				Adjusted accuracy % ^c^			Decrease % ^d^		
	50K	1K	3K	5K	1K	3K	5K	1K	3K	5K
**CED**	79.17	78.03	79.02	79.07	98.55	99.80	99.87	1.45	0.20	0.13
**BW**	77.21	75.88	77.08	77.16	98.27	99.82	99.93	1.73	0.18	0.07
**WW**	85.97	85.24	85.90	85.92	99.15	99.92	99.95	0.85	0.08	0.05
**YW**	88.09	87.41	88.01	88.03	99.23	99.91	99.93	0.77	0.09	0.07
**RADG**	74.58	72.99	74.43	74.45	97.87	99.80	99.82	2.13	0.20	0.18
**YH**	87.65	87.08	87.58	87.60	99.35	99.92	99.94	0.65	0.08	0.06
**SC**	79.93	78.69	79.70	79.75	98.46	99.72	99.79	1.54	0.28	0.21
**Doc**	80.52	79.50	80.44	80.45	98.74	99.89	99.91	1.26	0.11	0.09
**CEM**	80.33	79.19	80.14	80.17	98.59	99.77	99.80	1.41	0.23	0.20
**Milk**	83.78	82.92	83.74	83.76	98.97	99.95	99.97	1.03	0.05	0.03
**MW**	87.19	86.47	87.10	87.12	99.16	99.89	99.91	0.84	0.11	0.09
**MH**	88.46	87.79	88.38	88.39	99.25	99.91	99.92	0.75	0.09	0.08
**CW**	83.71	82.90	83.67	83.70	99.02	99.94	99.98	0.98	0.06	0.02
**Marb**	80.62	79.80	80.52	80.53	98.98	99.88	99.89	1.02	0.12	0.11
**Fat**	63.52	60.52	62.76	62.75	95.27	98.79	98.78	4.73	1.21	1.22
**$EN**	87.36	86.69	87.26	87.28	99.24	99.89	99.91	0.76	0.11	0.09
**$F**	86.74	86.10	86.68	86.70	99.27	99.94	99.95	0.73	0.06	0.05
**$G**	79.27	78.59	79.19	79.19	99.14	99.90	99.90	0.86	0.10	0.10
**$QG**	80.33	79.43	80.22	80.23	98.87	99.86	99.87	1.13	0.14	0.13
**$YG**	71.38	70.41	71.22	71.25	98.65	99.79	99.82	1.35	0.21	0.18
**$B**	87.58	86.97	87.52	87.53	99.31	99.93	99.94	0.69	0.07	0.06
**Mean**	81.59	80.60	81.45	81.48	98.73	99.82	99.85	1.27	0.18	0.15
**SD**	6.32	6.79	6.44	6.44	0.88	0.24	0.25	0.88	0.24	0.25

^a^ CED = Calving Ease Direct; BW = Birth weight; WW = Weaning Weight; YW = Yearling Weight; RADG = Residual Average Daily Gain; YH = Yearling Height; SC = Scrotal Circumference; Doc = Docility; CEM = Calving Ease Maternal; Milk = Maternal Milk Yield; MW = Mature Weight; MH = Mature Height; CW = Carcass Weight; Marb = Marbling Score; Fat = Fat Thickness; $EN = Cow energy value index; $F = feedlot value index; $G = grid value index; $QG = $ quality grade index; $YG = $ yield grade index; $B = beef value index.

^b^ Prediction accuracy = correlation between EPD and GEBV obtained from the original 50K genotypes and the 50K genotypes imputed from the 1K, 3K and 5K LD genotypes, respectively.

^c^ Adjusted accuracy = prediction accuracy using 50K genotypes imputed from 1K, 3K, and 5K chips, respectively, expressed as a percent over the accuracy obtained using the original 50K genotypes.

^d^ Decrease% = Percent decrease in prediction accuracy obtained using 50K genotypes imputed from 1K, 3K, and 5K, respectively, over the prediction accuracy obtained using the original 50K genotypes.

**Fig 12:**
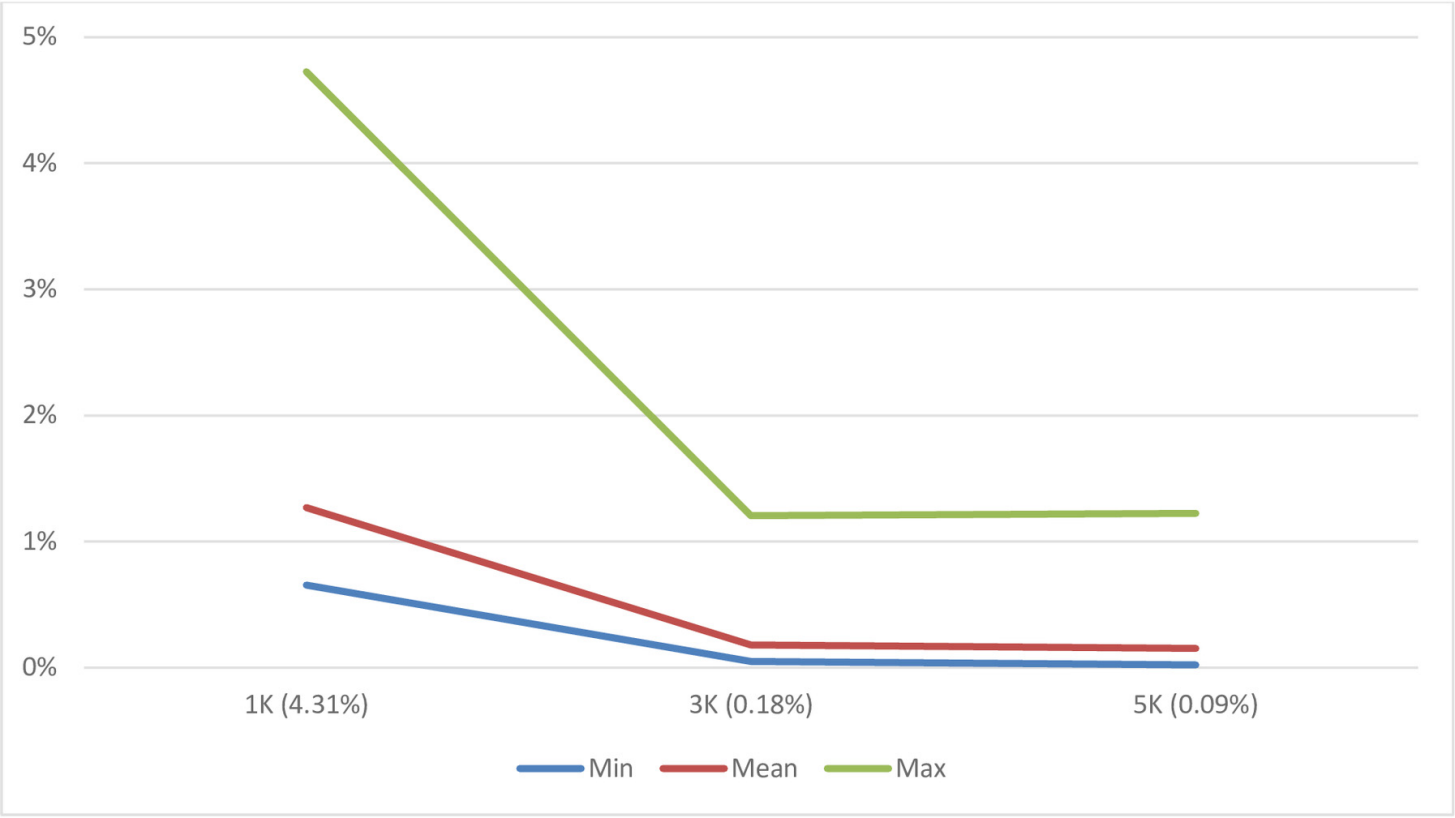
Line-curve plots of maximum (Max, upper curve), mean (Mean, middle curve) and minimum (Min, lower curve) error rate of genomic prediction using 50K genotypes imputed from 1K, 3K and 5K LD genotypes in an Angus population. Each line-curve represents a statistic value averaged for 21 quantitative traits.

Nevertheless, the relative prediction accuracy (i.e., the genomic prediction accuracy using imputed 50K genotypes expressed as a percentage of that achieved using the original 50K genotypes) was 98.73%, 99.82%, and 99.85%, respectively, for the three ULD panels. These results suggests that the difference in prediction accuracy obtained using the complete 50K and imputed 50K genotypes is very slight. The average ± SD for the prediction accuracy loss over the 21 traits was 1.27 ± 0.88%, 0.18 ± 0.24%, and 0.15% ± 0.25% when the prediction was made using imputed 50K genotypes from 1K, 3K, and 5K LD genotypes, respectively, as compared to genomic prediction using the original 50K genotypes. For the 3K and 5K ULD panels, the loss of accuracy in genomic prediction from using imputed 50K genotypes was very small (Fig 12).

#### Propagation of imputation error to genomic prediction

The genomic prediction error rate was regressed on the imputation error from the three ULD SNP chips used for imputation and genomic prediction. The regression coefficient ± SE was 0.2594 ± 0.0318, which was statistically very different from zero (P = 1.32 × 10^−11^). Based on this regression coefficient, we conclude that approximately 25% of the imputation error was propagated into the genomic prediction in this Angus population.

Analytically, the propagation of imputation error into genomic prediction can be shown as follows. Consider one individual, say *i*, which is genotyped for a total of *K* SNP loci spanning the genome. For simplicity, assume that all systematic factors (including the overall mean) are not relevant (or have already been adjusted from the data). Then, a trait value can be described by the following linear equation:

$$y_{i}=x_{i}^{'}a+e_{i}$$ (16)

where *y*_*i*_ is an adjusted phenotypic value or breeding value for the *i*^th^ individual, ***x***_*i*_ is a *K* × 1 vector of SNP genotypes (e.g., coded as 0, 1, 2), ***a*** is a *K* × 1 vector of additive genetic effects of these SNPs, and *e*_*i*_ is the residual term. Then, the prediction accuracy using this set of genotypes is measured by the following correlation:

$$r_{o}=Cor(y_{i},x_{i}^{'}\hat{a})$$ (17)

where $\hat{a}$ is a *K* × 1 vector of estimated additive effect for these SNPs, as derived previously from a training population. In the genomic prediction context, a reasonable range is: 0 ≤ *r*_*o*_ ≤ 1. In reality, negative correlations are possible, but they are rare and are considered to be unreasonable outcomes. Hence, negative accuracy (correlation) is not considered here.

Next, let ***x***_***i*,*c***_ be a vector which contains a subset of the *K* genotypes that are correctly obtained, and ***x***_***i*,*M***_ be a vector of the remaining SNPs that are not correctly imputed (i.e., mis-imputed). Likewise, the SNP effect vector ***a*** can be split into ***a***_*c*_ and ***α***_*M*_, where the subscripts *c* and *M* index the two sets of SNPs respectively. The estimated total genetic value of the *i*^th^ individual is then given as:

$$\hat{y_{i}}=x_{i,c}^{'}a_{c}+x_{i,M}^{'}a_{M}$$ (18)

Assume that ***a***_***c***_ and ***a***_***M***_ are not correlated, and neither are ***x***_*i*,*c*_ and ***x***_*i*,*r*_. Then, the prediction accuracy using imputed genotypes is computed as:

$$\begin{array}{l} r_{I}=Cor\left(y_{i},x_{i,c}^{\prime }{\hat{\alpha }}_{c}+x_{i,M}^{\prime }{\hat{a}}_{M}\right)\\ \;=Cor\left(y_{i},x_{i,c}^{\prime }{\hat{\alpha }}_{c}\right)+Cor\left(y_{i},x_{i,M}^{\prime }{\hat{a}}_{M}\right)\\ \;=r_{c}+r_{M} \end{array}$$ (19)

In the above, we assume that mis-imputed SNPs are consistent across all these SNPs, meaning that certain SNPs tend to be mis-imputed for all animals and the remaining can be reliably imputed. Note that this assumption may not hold in reality, but it simplifies our discussion. With this assumption, $r_{M}=Cor(y_{i},x_{i,M}^{'}{\hat{a}}_{r})$ is the portion of the total correlation due to the use of mis-imputed genotypes. Note that *r*_*M*_ can take negative values if the imputed genotypes are mostly opposite to the true genotypes (e.g., AA -> BB or vice versa). A reasonable upper bounder for *r*_*M*_ is *r*_*M*_ ≤ *r*_*o*_ − *r*_*C*_. This is equivalent to saying that:

$$r_{I}=r_{c}+r_{M}\leq r_{c}+(r_{o}-r_{C})=r_{o}$$

$$r_{I}=r_{c}+r_{M}\leq r_{c}+r_{r}=r_{o}$$ (20)

Therefore, *r*_*I*_ ≤ *r*_*o*_, because genomic prediction using imputed genotypes cannot be more accurate than that using original genotypes.

In reality, the loss of prediction accuracy apparently depends on which subset of SNPs is mis-imputed. When SNPs that all have large association effects are mis-imputed, the loss of prediction accuracy can be substantial. But if mis-imputed genotypes are all for SNPs which all have tiny or no effects on the trait, the loss of prediction accuracy can be ignored. In the simplest case, assume that prediction ability using mis-imputed genotypes is zero at the worst, and that negative correlation will not happen. Then, the accuracy of prediction using imputed genotypes can be reasonably bounded between *r*_*c*_ and *r*_*o*_:

$$r_{c}\leq r_{I}\leq r_{o}$$ (21)

But keep in mind that it is likely to have *r*_*I*_ < 0 in real situations.

Back to the problem at hand, when LD genotypes are imputed to moderate- and high-density genotypes, if imputation error rate is, say < 1%, the loss due to imputation errors tends to also be small, and the consequences of propagation of imputation error can be minimal.

### Imputation-Mediated Genomic Prediction in U.S. Holsteins

The scenario for imputation-mediated genomic prediction is the following: instead of genotyping all candidate animals on GGP HD SNP chips, it is cost-effective to genotypes these animals on LD chips and then impute LD genotypes to 80K genotypes for genomic prediction. In this part of the study, the MOLO algorithm was used to improve the performance of LD SNP chips in an imputation-mediated genomic prediction. The genomic prediction system was built with SNP effects estimated on 80K genotypes for three traits in 7,012 U. S. Holstein animals. Three LD 6K SNP panels were evaluated. The 6KA panel consisted of 5,260 unique SNPs pooled from each of the 2,000 SNPs with the largest SNP variances for each trait. The 6KB panel included all the SNPs in the 6KA panel, plus additional 740 SNPs which were optimally selected by the selectSNP package. The 6KC panel consisted of 6,000 SNPs with the largest average SNP variances on average for the three traits.

These 6KA panel accounted for 31.1%, 41.8% and 38.9%, respectively, of the total SNP variances for DPR, FY and MY in this U.S. Holstein population (See S2 Fig). The amended 6KB panel accounted slightly more of the total SNP variances (31.8–42.4%) with the inclusion of additional 740 SNPs panels (S2 Fig). The multiple-trait 6KB panel accounted for the greatest percentages (35.7–44.5%) of the total SNP variance in the three LD 6K SNP panels (S2 Fig). When using only 6K SNP genotypes in the prediction while ignoring the impact of the remaining SNPs, the corresponding genomic prediction accuracies (GPA) were 81.57–83.56% for DPR, 82.14–84.43% for FY, and 77.32–80.79% for MY for these three LD 6K SNP panels (S5 Table). Compared to genomic prediction using original 80K genotypes, the relative genomic prediction accuracies (RGPA) were 88.05–90.20% for DPR, 97.23–89.67% for FY, and 83.80–97.56% for MY when using 6K genotypes only (S5 Table). Of the three LD 6K panels, the 6KC panel had the greatest genomic prediction accuracies, followed by the 6KB panel, and the 6KA panel had the lowest genomic prediction accuracies in the 2,639 U. S. Holstein animals (S5 Table). For the three LD 6K SNP panels, the order of genomic prediction agreed with that of SNP variance contributions when using 6K SNP genotypes directly for genomic prediction. The panel with the largest portion of SNP variances had the greatest genomic prediction accuracies.

In addition to SNP variance contributions, imputation accuracy also played a critical role in the resulting genomic prediction using imputed 80K genotypes. SNPs selected based SNP-trait association were very unevenly-distributed (S3A Fig). Adding 740 optimally selected SNP has drastically improve SNP distribution (S3B Fig). When LD 6K genotypes were imputed to 80K genotypes, the imputation error rate were roughly comparable between the 6KA panel. On average, the imputation error rate was 5.30% for the 6KA panel, ranging from 1.85% to 11.71% for each chromosome, and it was slightly lower (4.59%) for the 6KC panel, ranging from 1.68% to 10.59% (S4 Table). Given similar imputation error rate, because the SNPs in the 6KC panel accounted for 6.50–14.79% more SNP variance than the 6KA panel, the 6KC panel consistently had better genomic prediction on the three traits than the 6KA panel. The current results has favorably supported the multiple-trait approach for selecting LD SNPs over the single-trait approach, yet the superiority of the former over the latter apparently depends on the portion of SNPs commonly important to all traits. Detailed discussions on this issue were not given here because it was not the focus of this paper.

By adding 740 optimally selected SNPs, it has obviously improve SNP distributions on the genome (S3B Fig). Consequently, the imputation accuracy with the 6KB panel was increased significantly and the imputation error rate was the lowest and also with the smallest variation (standard deviation) among chromosomes in the three LD 6K SNP panels. On average, the imputation error rate across the 30 chromosomes were between 1.34% and 7.56% with the 6KB panel. It is noted that the maximum imputation error for the 6KB panel was 71.39% as much as that for the 6KA panel and 64.56% as much as that for the 6KC panel. Though the 6KB panel accounted for less SNP variances than the 6KC panel, because of the significantly decrease in imputation error rate, the 6KB panel otherwise had the greatest genomic prediction accuracy, even better than the 6KC panel (S5 Table). These results have evidently demonstrated that the improvement of imputation-mediated genomic prediction accuracy by including optimally-selected, informative LD SNPs, and the selectSNP package (LOMO algorithm) is a good tool to achieve this goal.

## Discussion and Conclusions

The effective utilization of genomic information for genetic evaluation and selection is of primary interest in the post-genome-era of agricultural genomic applications, and, at its core, the focus has been shifted from identifying with high power the DNA variants that contribute to a disease or a trait of economic importance to predicting with desirable accuracy the total genetic merit of individuals using a set of SNPs spanning the genome. This is delivered by the “genomic prediction” techniques, and LD SNPs have emerged as a cost-effective solution toward this effort. LD SNP chips can be either trait-specific or generally applicable to all traits. Often, the former includes a subset of SNPs specifically selected for the trait of interest (i.e., based on SNP-trait associations), whereas the latter consists of evenly-spaced SNPs, or approximately so, either with or without a threshold for MAF (and phenotypes are not directly relevant to the selection of SNPs). These are the two fundamental approaches that have been applied to design LD SNP chips and neither of these two approaches is optimal. Evenly-spaced SNPs may not be optimally informative if only map positions of SNPs are considered. On the other hand, while a trait-specific LD chip can better capture SNP-trait associations, the use of trait-specific chips often requires a much higher overhead cost when multiple traits are involved, because many chips are needed. Besides, genomic prediction using LD chips can suffer from a considerable loss of information as compared to the case when moderate- and high-density SNP genotypes are used.

In this paper, we have proposed a multiple-objective, local optimization (MOLO) algorithm for the optimal design of LD SNP chips that can be used to impute LD genotypes to moderate- or high-density SNP genotypes with considerably desirable accuracy. The objectives are to maximize non-gap map length and the system information for a chip, and the latter is computed either as locus-averaged or haplotype-averaged Shannon entropy and is adjusted for the uniformity of SNP locations on the chromosomes, while taking into consideration a number of equality and/or inequality constraints. Information based on allele frequencies is not the sole decisive factor, but an important one that needs to be considered when design low-density chips. Given a list of obligatory SNPs, the optimization is conducted conditionally on the presence of the SNPs that have been assigned to the chip map prior to the design optimization. The frame design of a SNP chip can be either of uniform or non-uniform. In the former case, the algorithm selects a set of highly informative SNPs that are evenly-spaced or approximately so within local search ranges which are decided according to a tuning parameter 0 ≤ *γ* ≤ 0.5. For non-uniform designs, a tunable empirical Beta distribution is used to guide the selection of highly informative SNPs so that the SNP density can be enriched to a varying extent towards the ends of chromosomes. Our results show that this MOLO algorithm can effectively increase the system information of the resulting LD SNP chips, which in turn leads to higher imputation accuracy as compared to the previous design methods which select evenly-spaced SNPs only. The imputation accuracy increases with LD chip size, e.g., from 1K to 3K and then to 5K, and the imputation error rate becomes very low with a SNP chip of size 3K or more. Other factors affecting imputation accuracy include the tuning parameter, information criteria, and type of SNP distributions on each chromosome. The propagation of imputation error rate to genomic prediction depends on whether or not the mis-imputed SNPs are in LD with causal loci affecting the trait of interest, as well as the magnitude of the associated effects of the SNPs that are mis-imputed. When LD SNP genotypes are imputed to higher-density genotypes with high accuracy, the error rate propagated to the subsequent genomic prediction is minimal, and the loss of prediction accuracy can be ignored. Therefore, we conclude that the MOLO algorithm can serve as an efficient tool for designing cost-effective, LD SNP chips for agricultural genomics applications. The MOLO algorithm is especially effective when used to design low-density SNP chips, for example with < 5K SNPs. The utility of the MOLO algorithm is also supported by a real genomic prediction application in U.S. Holstein animals.

## Supporting Information

S1 Fig Composition (i.e., number of SNPs) of the common-variant 24K bovine SNP chip for seven cattle breeds. Holstein is a dairy cattle breed, and the remaining six are all beef breeds. The base and the backbone consisted of SNPs common to the seven cattle breeds. The number of SNPs selected for each category is listed by its category name (e.g., Limousin, 3000).
(DOCX)

S2 Fig Percentages of SNP variance on the three traits for the three 6K SNP panels. The three quantitative traits were daughter pregnancy rate (DPR), fat yield (FY) and milk yield (MY). Of the three LD 6K SNP panels, the 6KA panel consisted of 5,260 unique SNPs pooled from each of the 2,000 SNPs with the largest SNP variances for each trait. The 6KB panel included all the SNPs in the 6KA panel, plus additional 740 SNPs which were optimally selected by the selectSNP package. The 6KC panel consisted of 6,000 SNPs with the largest average SNP variances on average for the three traits.
(DOCX)

S3 Fig Map view of two LD 6K SNP panels. The 6KA panel consisted of 5,260 unique SNPs pooled from each of the 2,000 SNPs with the largest SNP variances for each trait. The 6KB panel included all the SNPs in the 6KA panel, plus additional 740 SNPs which were optimally selected by the selectSNP package. These SNPs were located on 30 chromosomes, of which Chromosome 30 is the X chromosome.
(DOCX)

S1 File selectSNP Manual V1.1 This is an users’ manual for the selectSNP package (trial version 1.1).
(PDF)

S2 File selectSNP Vignette V1.1 This is the vignette for the selectSNP package (trial version 1.1).
(PDF)

S1 Table Summary of maps for the 50K vs. 24K bovine SNP chips. The column names are nLoci (number of SNPs on each chromosome), Length (physical map length, in base pairs, of each chromosome), max.bw (maximum gap, in base pairs, on each chromosome). Chromosome 30 stands for X Chromosome.
(DOCX)

S2 Table Summary of the GGPHD 80K bovine SNP chip. The column names are N (number of SNPs per chromosome), and mean, SD (standard deviation), Min (minimum value), and Max (maximum value) of SNP spacing, where SNP spacing is defined as the map distance in base pairs between two adjacent SNPs on each chromosome.
(DOCX)

S3 Table Summary statistics of predicted transmitting ability (PTA) for daughter pregnancy rate (DPR), fat yield (FY), and milk yield (MY) in a U.S. Holstein population. The column names are N (sample size), and Min (minimum value), Q1 (25% quantile), Q3 (75% quantile), Max (maximum value), and Mean and SD (standard) deviation) of PTA.
(DOCX)

S4 Table Imputation accuracy from three sets of 6K SNP genotypes to 80K genotypes in U.S. Holstein animals. The reference population for imputation consisted of 7,012 Holstein animals, each genotyped by GGPHD 80K SNPs and the validation set had 2,639 Holstein animals also with 80K genotypes. For the purpose of evaluation imputation error, only selected 6K genotypes were kept while the genotypes of the remaining SNPs were all set to be missing. The last four rows are average, standard deviation (SD), minimum value, and maximum value of imputation accuracy across 30 chromosomes, where chromosome 30 is the X chromosome.
(DOCX)

S5 Table Genomic prediction accuracy using three subsets of 6K SNP genotypes, imputed 80K genotypes, and original 80K genotypes, respectively, in 2,639 U.S. Holstein animals. SNP effects for genomic prediction were estimated on original 80K SNP genotypes in the reference population of 7,012 Holstein animals. Genomic prediction accuracy for three quantitative traits, namely daughter pregnancy rate (PDR), fat yield (FY) and milk yield (MY), were evaluated in the validation set of 2,639 Holstein animals. Genomic-estimated breeding values were computed either based on the three sets of selected 6K SNPs, or on imputed 80K genotypes obtained from the three sets of 6K SNP genotypes.
(DOCX)
