# Supplementary figures and images for: Optimal Design of Low-Density SNP Arrays for Genomic Prediction: Algorithm and Applications
Source: PLoS One. 2016 Sep 1;11(9):e0161719. doi: 10.1371/journal.pone.0161719 (PMC5008792; doi:10.1371/journal.pone.0161719)

(A)


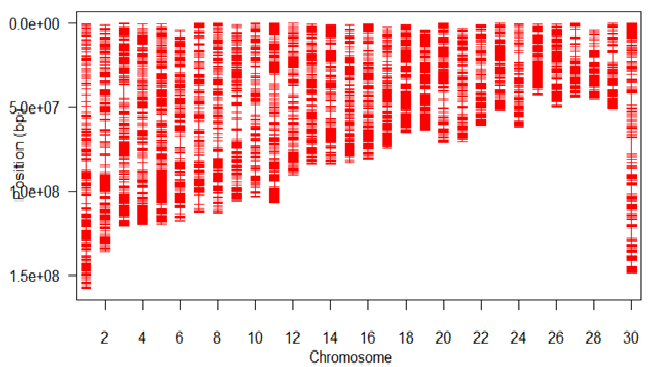


(B)


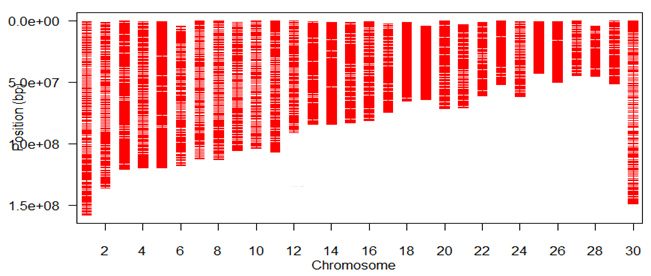

Supplement: S3 Fig — The 6KA panel consisted of 5,260 unique SNPs pooled from each of the 2,000 SNPs with the largest SNP variances for each trait. The 6KB panel included all the SNPs in the 6KA panel, plus additional 740 SNPs which were optimally selected by the selectSNP package. These SNPs were located on 30 chromosomes, of which Chromosome 30 is the X chromosome. (DOCX) [file pone.0161719.s003.docx]
