# Supplementary material for: Optimal Design of Low-Density SNP Arrays for Genomic Prediction: Algorithm and Applications
Source: PLoS One. 2016 Sep 1;11(9):e0161719. doi: 10.1371/journal.pone.0161719 (PMC5008792; doi:10.1371/journal.pone.0161719)
Supplement: S1 File — This is an users’ manual for the selectSNP package (trial version 1.1). (PDF) [file pone.0161719.s004.pdf]

Package ‘selectSNP’

March 30, 2016

Type Package

Title Select SNPs from a higher density panel

Version 1.06

Date 2014-08-25

Author Xiao-Lin (Nick) Wu

Maintainer Xiao-Lin (Nick) Wu <nickwu2003@gmail.com>; Jiaqi Xu<jiaqixu1@gmail.com>

Description Given a database of SNPs, this package selects a subset of high informative, evenly or approximately evenly distributed SNPs for genetic diagnoses or evaluation. It utilizes a heuristically local optimization function, which takes into account the distribution and location of SNPs, allelic frequencies, and type of substitutions. It also allows for pre-inclusion of a set of ``obligatory'' SNPs. In the simplest situation where only map information is used, this package selects a subset of SNPs that are evenly or approximately evenly distributed, subject to the database of the candidate SNPs and those that need to be pre-included.

Depends combinat

License The GNU general public license

RoxygenNote 5.0.1

NeedsCompilation no

R topics documented:

addUnifBounds 2
adjust.nInBin 3
adjust.nInBin2 3
cleanup.map 4
comparePanels 4
computeEntropy 5
conditional_betaBounds 6
conditional_unifBounds 7
demo60K 8
fillGaps 9
findBlocks 9
getBlocks 10
H.Shannon 10
loadLibrary 11

mapToLst 12
muH.Shannon 12
nInBins 13
nType 14
optimalCore 14
optimalPanel 16
optimizationMethods 17
percent 18
plot.map 19
randomPanel 19
readData 20
readMap 21
remove.Type 21
search.optima 22
select.Type 23
summary.map 23
unifBounds 24

Index **25**

`addUnifBounds` *The addUnifBounds function*

Description

This function add uniform boundary ticks.

Usage

```
addUnifBounds(x, from, to, n, ...)

## S4 method for signature 'missing,numeric,numeric,numeric'
addUnifBounds(from, to, n)

## S4 method for signature 'data.frame,missing,missing,missing'
addUnifBounds(x, gK = 2)
```

Arguments

`x`	a data frame as the output from the findBlocks function. It has three columns: lftB, rgtB, Length, and n, where n is the number of ticks to be inserted between lftB and rgtB.
`from`	The starting point
`to`	The end point
`n`	The number of boundary tikcs to create
`...`	parameters that apply.
`gK`	Times of bins for defining a gap

Value

A vector of boundary ticks

Author(s)

Nick X-L Wu

Examples

```
out1<-addUnifBounds(from=0,to=1,n=11)
#data(demo60K)
#out2<-addUnifBounds(demo60K,gK=4)
```

---

adjust.nInBin *adjust counts to be filled in Bins*

Description

This function adjusts the number of SNPs to be filled in bins, subject to the actual number of existing SNPs

Usage

```
adjust.nInBin(x, ni, block)
```

Arguments

x	A numeric vector of data points (e.g. map positions)
ni	An integer vector for nubmers within bins
block	A matrix that defines the blocks, with colums for left boundary, right boundary and interval length

Author(s)

Nick X-L Wu

---

adjust.nInBin2 *adjust counts to be filled in Bins*

Description

This function adjusts the number of SNPs to be filled in bins, subject to the actual number of existing Obligatory SNPs

Usage

```
adjust.nInBin2(obls, ni, block)
```

Arguments

`obls`	A nueric vector for the map position of obligatory SNPs
`ni`	An integer vector for nubmers within bins
`block`	A matrix that defines the blocks, with colums for left boundary, right boundary and interval length

Author(s)

Nick X-L Wu

`cleanup.map` *The cleanup.map function*

Description

This function cleans up a map data by removing NA data and exluding SNPs on chromosomes 0, Y and MT. The X chromosome will be re-coded to have a maximum number for all the chromosomes.

Usage

```
cleanup.map(map)
```

Arguments

`map`	A data frame, which has at least three columns for SNP names (SNP), Chromosome, and Position.

Author(s)

Nick X-L Wu

`comparePanels` *The comparePanels function*

Description

This function compares panels optimized using uniform and beta models. Additionally, a random panel is included in the comparison as well.

Usage

```
comparePanels(allsnp, oblsnp, pnlSize, method.set = c("Uniform", "Beta"),
  rbin.set = c(0, 0.1, 0.2, 0.3, 0.4, 0.5, 0.6, 0.7, 0.8, 0.9, 1), c = 1,
  m = 1, type = "singH")
```

Arguments

`allsnp`	Input data file or data frame that contains all SNPs from which the optimal panel is to selected. The data should contain the following columns: SNP, Chromosome, Position, Maf, Type, and Status.
`oblsnp`	A data frame for obligatory SNPs, which should contain the following columns: SNP, Chromosome, Position, Maf, Type, and Status. In particular, all Status should have values of 1s.
`pnlSize`	Number of SNPs in the panel
`method.set`	A set of methods, which by default inlcude "uniform" and "beta".
`rbin.set`	A set of bin intervals for r.bw, which by default are 0 to 1, step by 0.1.
`c`	The paramater that empirically tunes the weights for adjusting Shannon Entropy according to SNP distributions on the maps. The larger c, the smaller weights, and the less adjustment will be made. By default, c = 1, which exercise the adjustment.
`m`	The paramete that specify how many SNP to be selected on each bin.
`type`	Type of method to optimize the information, either on single SNPs (singH) or mutliple SNPs (multH). By default, type = singH.

Author(s)

Nick X-L Wu

`computeEntropy` *Compute Shannon entropy based on allelic frequency*

Description

This functions computes Shannon entroyp for each locus, assuming the independence of each locus.

Usage

```
computeEntropy(x, z, outFile)
```

Arguments

`x`	It can be either a vector of allelelic frequencies for all loci involved, or a data frame which contains two columns, one for SNP names (SNP), and the other for allelic frequency. The second column name can be either "Freq" or "Maf" (which is not case insensitive).
`z`	A data frame that contains alternative minor allelic frequency. It must have two columns, one for SNP names (SNP), and the other for minor allelic frequency (Maf). If z is present, Shannon entropy is computed based on z, instead of x.
`outFile`	When the output file name is provided, the histogram is saved in this output file; otherwise, it is displayed on the monitor.

Value

It returns a vecotor of computed Shannon entropy for each locus

Author(s)

Nick X-L Wu

Examples

```
x<-data.frame(SNP=paste("M",1:100,sep=""),
              Freq=runif(100))
out<-computeEntropy(x)
```

---

conditional_betaBounds

The conditional_betaBounds function

Description

This function is used to generate beta bounds condidtional on data

Usage

```
conditional_betaBounds(x, nSel, obls = numeric(), tailed = "more",
  gK = 1.5, maf = numeric(), r.bw = 0, c = 1, m = 1, type = "singH")
```

Arguments

x	A numeric vector of data points (e.g. map positions)
nSel	Number of ticks to be created
obls	A numeric vector containing map data for obligatory SNPs, which by default is empty
tailed	A logical value which indicate if "less" or "more" enrichment of SNPs on both ends of a chromosomes should be enforced.
gK	Times of bins for defining a gap
maf	A numeric vector of minor allele frequecies of all the candidate SNPs
r.bw	Times of bandwidth that defines a local region for search the optimal data point.
c	The paramater that empirically tunes the weights for adjusting Shannon Entropy according to SNP distributions on the maps. The larger c, the smaller weights, and the less adjustment will be made. By default, c = 1, which exercise the adjustment.
m	The paramete that specify how many SNP to be selected on each bin.
type	Type of method to optimize the information, either on single SNPs (singH) or mutliple SNPs (multH). By default, type = singH.

Author(s)

Nick X-L Wu

Examples

```
## Not run:
   x<-sort(runif(500))
   maf<-runif(500,0,0.5)
   out1a<-conditional_betaBounds(x=x,nSel=50,tailed="more")
   mean(computeEntropy(out1a))
   out1b<-conditional_betaBounds(x=x,nSel=50,tailed="more",
                                maf=maf,r.bw=0.25)
   mean(computeEntropy(out1b))
   obls<-sort(sample(x,10,replace=FALSE))
   out2a<-conditional_betaBounds(x=x,nSel=50,obls=obls,tailed="more")
   out2b<-conditional_betaBounds(x=x,nSel=50,obls=obls,tailed="more",
                                  maf=maf,r.bw=0.15)
   plot.map(list(x,out1a,out1b,obls,out2a,out2b))

## End(Not run)
```

---

conditional_unifBounds

The conditional_unifBounds function

Description

This function is used to conduct local search of optima in an approximately uniform design

Usage

```
conditional_unifBounds(x, maf = numeric(), nSel, obls = numeric(),
  gK = 1.5, r.bw = 0, c = 1, m = 1, type = "singH")
```

Arguments

`x`	A numeric vector of map positions of all the candidate SNPs
`maf`	A numeric vector of minor allele frequecies of all the candidate SNPs
`nSel`	Number of ticks to be placed on the map
`obls`	A numeric vector containing map data for obligatory SNPs, which by default is empty.
`gK`	Times of bins for defining a gap, which by default has a value of 2.0 (i.e., a gap is identified as 2.0 times as wide as an average bin)
`r.bw`	Times of bandwidth that defines a local region for search the optimal data point.
`c`	The paramater that empirically tunes the weights for adjusting Shannon Entropy according to SNP distributions on the maps. The larger c, the smaller weights, and the less adjustment will be made. By default, c = 1, which exercise the adjustment.
`m`	The paramete that specify how many SNP to be selected on each bin.
`type`	Type of method to optimize the information, either on single SNPs (singH) or mutliple SNPs (multH). By default, type = singH.

Author(s)

Nick X-L Wu

Examples

```
x<-sort(runif(50,0,100))
maf<-runif(50,0,0.5)
out1a<-conditional_unifBounds(x=x,nSel=10)
mean(computeEntropy(x=maf[x%in%out1a]))
out1b<-conditional_unifBounds(x=x,nSel=10,maf=maf,r.bw=0.5)
mean(computeEntropy(maf[x%in%out1b]))
obls<-sort(sample(x,5,replace=FALSE))
out2a<-conditional_unifBounds(x=x,nSel=20,obls=obls)
mean(computeEntropy(maf[x%in%out2a]))
out2b<-conditional_unifBounds(x=x,nSel=20,obls=obls,maf=maf,r.bw=0.05)
mean(computeEntropy(maf[x%in%out2b]))
plot.map(list(x,out1a,out1b,obls,out2a,out2b))
```

---

demo60K *The demostration 60K SNP map dataset*

Description

This is a demonstration 60K map data, which consists of 60,472 bovine SNPs.

- SNP. SNP names
- Chromosome. Names (indexes) of Chromosomes.
- Maf. Minor allelelic frequency, which ranges betwwen 0 and 0.5.
- Type. SNP variant types, which has a value of A, B, or C.
- Status. A single digit indicate whether each SNP is "obligatory" or not, where 1 = obligatory SNP and 0 = non-obligatory SNPs. Obligatory SNPs need to be pre-included when select SNPs.

Usage

```
data(demo60K)
```

Format

A data frame with 60,784 rows and 6 variables

`fillGaps` *The fillGaps function*

Description

This function fill gaps in the target map (x) using SNP from a reference map (ref)

Usage

```
fillGaps(x, ref, gK = 2, n)
```

Arguments

`x`	The target map in which the gaps are to be filled.
`ref`	The reference map from which SNPs will be taken.
`gK`	Times of bins for defining a gap
`n`	Number of SNPs to be inserted.

Author(s)

Nick X-L Wu

`findBlocks` *The findBlock function*

Description

This function is used to obtain a matrix for gap blocks

Usage

```
findBlocks(x, gK = 2)
```

Arguments

`x`	A numeric vector of data points (e.g. map positions)
`gK`	Times of bins for defining a gap

Author(s)

Nick X-L Wu

Examples

```
x<-sort(runif(50))
findBlocks(x)
```

getBlocks *The getBlocks function*

Description

This function is used to obtain a matrix for linkage blocks

Usage

```
getBlocks(x, obls = numeric(), nSel, gK = 1.5)
```

Arguments

x	A numeric vector of data points (e.g. map positions)
obls	A numeric vector containing map data for obligatory SNPs, which by default is empty.
nSel	Number of ticks to be created
gK	Times of bins for defining a gap

Author(s)

Nick X-L Wu

Examples

```
x<-sort(runif(50))
blocks1<-getBlocks(x=x,nSel=30)
ticks1<-sort(unique(unlist(blocks1[,1:2])))
obls<-sort(sample(x,10,replace=FALSE))
blocks2<-getBlocks(x=x,nSel=30,obls=obls)
ticks2<-sort(unique(unlist(blocks2[,1:2])))
plot.map(list(x,ticks1,obls,ticks2))
```

H.Shannon *Compute (adjusted) Shannon entropy per SNP basis*

Description

This function computes Shannon entropy for a single or a set of SNP. With >= 3 SNPs, it also adjust the value of Shannon entropy by weighting toward more uniformness of the SNP distribution.

Usage

```
H.Shannon(maf, pos = NA, c = 1)
```

Arguments

maf	A scalar or a vector of frequences for either of the two alleles, or for the minor alleles, each pertaining a SNP.
pos	A scalar or a vector of map position, each pertaining to a SNP. When pos is not NA, adjusted Shannon entropy is computed.
c	A constant (c>=1) for adjusting the weighting value. The larger c value, the small weighting.

Author(s)

Nick X-L Wu

Examples

```
pos<-sort(runif(50,0,100))
maf<-runif(50,0,0.5)
plot(maf~pos,type="h")

H.Shannon(maf)
H.Shannon(maf,pos)
H.Shannon(maf,pos,c=10)
H.Shannon(maf,pos,c=100)

idx<-which.max(H.Shannon(maf,pos))
pos.sel<-pos[c(1,idx,50)]
maf.sel<-maf[c(1,idx,50)]
plot(maf.sel~pos.sel,type="h")
```

`loadLibrary` *The loadLibrary function*

Description

This function is used to assist load a library if not present

Usage

```
loadLibrary(lib)
```

Arguments

lib	A character string for the library name

Author(s)

Nick X-L Wu

mapToLst *The mapToLst function*

Description

This function is used to convert maps from a dataframe to a list

Usage

```
mapToLst(map, outFile)
```

Arguments

map	input map as a data frame, which should have at least three columns: one column for loci name (SNP), one column for chromosome (Chromosome), and one column for map position (Position)
outFile	If outFile is provide, the output will be save to this file.

Author(s)

Nick X-L Wu

Examples

```
map1<-data.frame(SNP=paste("M",1:10,sep=""),
  Chromosome=rep(1,10),Position=sort(runif(10)))
map2<-mapToLst(map1)
```

muH.Shannon *Compute (adjusted) mean Shannon entropy for multiple-SNPs*

Description

This function computes mean Shannon entropy for mutliple SNPs. To adjust mean Shannon entropy by SNP distribution, there should be at least 3 SNPs involved.

Usage

```
muH.Shannon(maf, pos = NA, c = 1)
```

Arguments

maf	A vector of frequences for either of the two alleles, or for the minor alleles, each pertaining a SNP.
pos	A a vector of map position, each pertaining to a SNP. When pos is not NA, mean Shannon entropy is adjusted by SNP distribution.
c	A constant (c>=1) for adjusting the weighting value. The larger c value, the small weighting.

Author(s)

Nick X-L Wu

Examples

```
pos<-sort(runif(5,0,100))
maf<-runif(5,0,0.5)
plot(maf~pos,type="h")

muH.Shannon(maf)
muH.Shannon(maf,pos)
muH.Shannon(maf,pos,c=10)
muH.Shannon(maf,pos,c=100)
```

`nInBins` *The nInBins function*

Description

This function is used to get counts in each bin

Usage

```
nInBins(n, ratio)
```

Arguments

`n`	Total number of data points
`ratio`	Ratio of data points to be assigned to each bin

Author(s)

Nick X-L Wu

Examples

```
nInBins(n=50,ratio=c(0.2,0.1,rep(0.04,10),0.1,0.2))
```

`nType` *Number of type A or/and type B SNPs.*

Description

On Illumina, SNPs are binned according to the number and type of beads needed for a working assay. Bins A and B use 2 beads to deal with ambiguous bases (A/T or C/G) where the same dye is used for each base. Bin C uses 1 bead for assays that use two different dyes.

Usage

```
nType(x, type = "A")
```

Arguments

`x`	A vector of SNP types (e.g., "A", "B", "C").
`type`	Count SNPs for type A, or type B or both (AB)

Author(s)

Nick X-L Wu

`optimalCore` *The optimalCore function*

Description

This functions selects SNPs using a local optimizaiton algorithm, with initial SNP locations being either uniform or following a Beta distribution.

Usage

```
optimalCore(dataFile, preFile = NA, outpath = NA, pnlSize = 3000,
  method = "Beta", tailed = "less", maf_cutoff = 0.05, r.bw = 0.05,
  c = 1, m = 1, type = "singH")
```

Arguments

`dataFile`	A data file or data frame as the source SNP database, from which a subset of SNPs are to be selected. It must contain columns such as SNP names (SNP), chromosome index (Chromosome), map position (Position), minor allele frequency (Maf), variant type (Type) and status of usage (Status). If missing, the data set (demo60K) attached with this package is used. When this parameter value is missing, the built-in data demo60K will be used instead.
`preFile`	A data file or data frame for the "obligatory" SNPs, should they be preincluded. The data frame must contain columns for SNP names (SNP), chromosome index (Chromosome), map position (Position), minor allele frequency (Maf), variant type (Type) and status of usage (Status).
`outpath`	Path where the output files are to be saved.

pnlSize	Panel size, i.e., Number of SNPs to be chosen. For example, if the target panels will contain 1000 SNPs, then set pnlSize = 1000.
method	Type (method) for initial distributing SNPs, which can be either of the two values: Unif or Beta.
tailed	This parameter defines whether and how the SNPs are to be enriched on both ends of the chromosomes, empirically following a Beta distribution. It currently has two options: "less" or "more". Empirically, it divides the whole chromosome into 20 bins. Denote the first two bins on the left end as lftB, the last two bins on the right end as the rgtB, and the 16 bins in between as MidB. If "more" is chosen, it allocates 29 PCT of SNPs on either leftB or rgtB, and 42 PCT of the SNPs on the MidB. The "less" option allocates less SNPs on both ends, that is, 22 PCT, 56 PCT and 22 PCT on lftB, MidB, and rgtB, respectively.
maf_cutoff	A cutoff value for minor allele frequency, which by default is 0.05.
r.bw	This parameter defines the radius from the center of a "local region" to its edge. The "local region" is characterized by its bandwidth (bw), that is, a subset within a region: abs(x â€“ center_location) <= bw, where bw is calculated as: (max(x) â€“ min(x))/(length(x)-1).
c	The paramater that empirically tunes the weights for adjusting Shannon Entropy according to SNP distributions on the maps. The larger c, the smaller weights, and the less adjustment will be made. By default, c = 1, which exercise the adjustment.
m	The paramete that specify how many SNP to be selected on each bin.
type	Type of method to optimize the information, either on single SNPs (singH) or mutliple SNPs (multH). By default, type = singH.

Value

It returns a data.frame for the selected SNP panel.

Author(s)

Nick X-L Wu

Examples

```
data(demo60K)

L5000<-optimalCore(dataFile=demo60K,
                  maf_cutoff=0,
                  pnlSize=4644,
                  method="beta",
                  tailed="more",
                  r.bw=0.25)
```

optimalPanel *Wrapper of the optimalCore function*

Description

This is a wrapper of the optimalCore function, which selects a panel of SNPs based on either an uniform model or a beta model, optimized using a heuristically local optimization algorith.

Usage

```
optimalPanel(allsnp, oblsnp, cansnp, nCSet = 3, maf_cutoff = 0.05, prefix,
  pnlSize, method = "Beta", tailed = "less", r.bw = 0.25, nBacSNP = 0,
  nYSNP = 0, nA = 0, nB = 0, c = 1, m = 1, type = "singH")
```

Arguments

allsnp	A data file or data frame as the source SNP database, from which a subset of SNPs are to be selected. It must include the following columns SNP(SNP names), Chromosome(chromosome index), Positin(map position), MAF(minor allele frequency), Type(variant type) and Status(status of usage).
oblsnp	A data file or data frame for the "obligatory" SNPs, should they be preincluded. The data frame must contain columns for SNP names (SNP), chromosome index (Chromosome), map position (Position), minor allele frequency (Maf), variant type (Type) and status of usage (Status).
cansnp	A data file or data frame for candidate SNPs, should they be pre-included. This set of SNPs needs to be duplicated three times. The data frame must have columns for SNP names (SNP), chromosome index (Chromosome), map position (Position), minor allele frequency (Maf), variant type (Type) and status of usage (Status).
nCSet	Number of sets of candidate genes to be included. Its default value is 3, meaning that candidate genes are duplicated three times.
maf_cutoff	A cutoff value for minor allele frequency, which by default is 0.05.
prefix	A prefix to be added to the output panel name.
pnlSize	Number of SNPs to be chosen (i.e., panel size). For example, if the target panels will contain 1000 SNPs, then set pnlSize = 1000.
method	Type (method) for initial distributing SNPs, which can be either of the two values: Unif or Beta.
tailed	This parameter defines whether and how the SNPs are to be enriched on both ends of the chromosomes, empirically following a Beta distribution. It currently has two options: "less" or "more". Empirically, it divides the whole chromosome into 20 bins. Denote the first two bins on the left end as lftB, the last two bins on the right end as the rgtB, and the 16 bins in between as MidB. If "more" is chosen, it allocates 29 PCT of SNPs on either leftB or rgtB, and 42 PCT of the SNPs on the MidB. The "less" option allocates less SNPs on both ends, that is, 22 PCT, 56 PCT and 22 PCT on lftB, MidB, and rgtB, respectively.
r.bw	This parameter defines the radius from the center of a local region to its edge. The local region is characterized by its bandwidth (bw), that is, a subset within a region: abs(x â€“ center_location) <= bw, where bw is calculated as: (max(x) â€“ min(x))/(length(x)-1). Its default value is set up to be 0.25.

nBacSNP	Number of slots need to be reserved for bacteria SNPs. Its default value is zero.
nYSNP	Number of slots reserved for SNPs on Y chromosome. Its default value is zero.
nA	Minimum number of Bin A type SNPs to be included. On Illumina, SNPs are binned according to the number and type of beads needed for a working assay. Bins A and B use 2 beads to deal with ambiguous bases (A/T or C/G) where the same dye is used for each base. Bin C uses 1 bead for assays that use two different dyes.
nB	Miniumum number of Bin B type SNPs to be included.
c	The paramater that empirically tunes the weights for adjusting Shannon Entropy according to SNP distributions on the maps. The larger c, the smaller weights, and the less adjustment will be made. By default, c = 1, which exercise the adjustment.
m	The paramete that specify how many SNP to be selected on each bin.
type	Type of method to optimize the information, either on single SNPs (singH) or mutliple SNPs (multH). By default, type = singH.

Value

It returns a data.frame that contains the optimized SNP panel.

Author(s)

Nick X-L Wu

---

optimizationMethods *Select of SNPs using the heuristically local optimization algorithm*

Description

This functions selects SNPs using a heuristically local algorithm, in which the objective function is defined based map position and information content (Shannon entropy).

Usage

```
optimizationMethods(map, pnlSize = 3000, method = "beta", r.bw = 0.25,
  tailed = "less", c = 1, m = 1, type = "singH")
```

Arguments

map	A data frame which contains columns such as SNP names (SNP), chromosome index (Chromosome), map position (Position), minor allele frequency (Maf), variant type (Type) and status of usage (Status).
pnlSize	Panel size, i.e., Number of SNPs to be chosen
method	Optimization method, which follows a "beta" design or a "unform" design, either on SNP basis or haplotype basis. The latter approach is deactivated in this trial version. please
r.bw	This parameter defines the radius from the center of a "local region" to its edge. The "local region" is characterized by its bandwidth (bw), that is, a subset within a region: abs(x â€“ center_location) <= bw, where bw is calculated as: (max(x) â€“ min(x))/(length(x)-1).

tailed	This parameter defines whether and how the SNPs are to be enriched on both ends of the chromosomes, when using the "local" algorithm.
c	The paramater that empirically tunes the weights for adjusting Shannon Entropy according to SNP distributions on the maps. The larger c, the smaller weights, and the less adjustment will be made. By default, c = 1, which exercise the adjustment.
m	The paramete that specify how many SNP to be selected on each bin.
type	Type of method to optimize the information, either on single SNPs (singH) or mutliple SNPs (multH). By default, type = singH.

Value

It returns an optimal SNP panel.

Author(s)

Nick X-L Wu

`percent` *convert to percentage notation*

Description

This function is used to convert a numeric number into percentage notation (

Usage

```
percent(x, digits = 2, ...)
```

Arguments

x	A numeric number (either decimal or interger)
digits	Number of digits for this percentage number.
...	parameters that apply.

Value

A percentage number.

Author(s)

Nick X-L Wu

Examples

```
x<-0.8547926
percent(x,digits=3)
```

plot.map *The plot.map function*

Description

This function is used to plot map from a mapLst data

Usage

```
## S3 method for class 'map'
plot(map, outFile)
```

Arguments

map	Either a data frame or a mapLst data. In the formal case, it needs to have at least three columns: one column for loci name (name), one column for chromosome (chrom), and one column for map location (location). In the latter case, it has map locations of all loci on each chromosome as a list). For each list, its a named vector of map location data.
outFile	If outFile is provide, the output will be save to this file.

Author(s)

Nick X-L Wu

Examples

```
name<-c(paste("A",1:10,sep=""),paste("B",1:10,sep=""))
chrom<-rep(1:2,each=10)
pos1<-sort(runif(10))
pos2<-seq(0,1,by=1/9)

map<-data.frame(SNP=name,Chromosome=chrom,Position=c(pos1,pos2))
plot.map(map)
```

randomPanel *The randomPanel function*

Description

This function randomly select a subset of SNPs from the input SNP data file or data frame.

Usage

```
randomPanel(pnlSize, allsnp)
```

Arguments

`pnlSize`	Number of SNPs in the panel
`allsnp`	Input data file or data frame that contains all SNPs from which the optimal panel is to selected. The data should contain the following columns: SNP, Chromosome, Position, Maf, Type, and Status.

Author(s)

Nick X-L Wu

`readData` *The readData function*

Description

This function is used to read data from a TEXT file

Usage

```
readData(file, header = TRUE, sep, skip = 0, as.is = TRUE,
  strip.white = TRUE, na.strings = c("NA", ".", "-/-"))
```

Arguments

`file`	An data file name, which has an extention name of ".csv", ".txt", or ".dat", respectively.
`header`	a logical value indicating whether the file contains the names of the variables as its first line. If missing, the value is determined from the file format: header is set to TRUE if and only if the first row contains one fewer field than the number of columns.
`sep`	the field separator character. Values on each line of the file are separated by this character. If sep = "" (the default for read.table) the separator is a "white space" which is one or more spaces, tabs, newlines or carriage returns.
`skip`	An integer specifying the number of lines of the data file to skip before beginning to read data.
`as.is`	the default behavior of read.table is to convert character variables (which are not converted to logical, numeric or complex) to factors. The variable as.is controls the conversion of columns not otherwise specified by colClasses. Its value is either a vector of logicals (values are recycled if necessary), or a vector of numeric or character indices which specify which columns should not be converted to factors.
`strip.white`	A logical value which is used only when sep has been specified, and allows the stripping of leading and trailing white space from unquoted character fields (numeric fields are always stripped).
`na.strings`	A character vector of strings which are to be interpreted as NA values. Blank fields are also considered to be missing values in logical, integer, numeric and complex fields.

Author(s)

Nick X-L Wu

Examples

```
## Not run:
    out<-readData("file001.txt")
    out<-readData("file002.txt")
    out<-readData("file003.csv")

## End(Not run)
```

---

readMap — *Read map data from a CSV TEXT file*

Description

This function inputs map data from a text file. It should have three required column: SNP (SNP names), Chromosome (chromosome names or index), and Position (map position).

Usage

```
readMap(file)
```

Arguments

`file`	An map data file name, which needs to be a text file.

Author(s)

Nick X-L Wu

Examples

```
## Not run:
    map<-readMap("myMapFile.csv")

## End(Not run)
```

---

remove.Type — *Remove Type A and B SNPs.*

Description

On Illumina, SNPs are binned according to the number and type of beads needed for a working assay. Bins A and B use 2 beads to deal with ambiguous bases (A/T or C/G) where the same dye is used for each base. Bin C uses 1 bead for assays that use two different dyes.

Usage

```
remove.Type(x, type)
```

Arguments

x	An data frame containing map data for SNPs.
type	Type of SNPs to be removed

Author(s)

Nick X-L Wu

`search.optima` *Search optima using various criteria*

Description

This function search optimal SNP or SNPs that maximize Shannon entropy, or adjusted Shannon entroyp, or centrally located. When multiple SNPs are involved, Shannon entropy is computed as the average of these SNPs, either with or without adjusted by location.

Usage

```
search.optima(maf, pos = NA, c = 1, m = 1, type = "singH")
```

Arguments

maf	A vector of frequences for either of the two alleles, or for the minor alleles, each pertaining a SNP.
pos	A a vector of map position, each pertaining to a SNP. When pos is not NA, mean Shannon entropy is adjusted by SNP distribution.
c	A constant (c>=1) for adjusting the weighting value. The larger c value, the small weighting.
m	Number of SNPs to be selected when type = "multH.
type	Type of search: singH = search based on Shannon entropy based on each of SNPs; multH = search based on mean Shannon entropy based on multiple SNPs; cent = search for the most centrally located SNP.

Author(s)

Nick X-L Wu

Examples

```
maf<-runif(10,0,0.5)
pos<-sort(runif(10,0,100))
plot(maf~pos,type="h",ylim=c(0,0.5))

idx<-search.optima(maf=maf,pos=pos,type="singH")
js<-c(1,idx,10)
plot(maf[js]~pos[js],type="h",ylim=c(0,0.5))

idx<-search.optima(maf=maf,pos=pos,type="cent")
js<-c(1,idx,10)
plot(maf[js]~pos[js],type="h",ylim=c(0,0.5))
```

```
idx<-search.optima(maf=maf,pos=pos,c=1,type="multH")
plot(maf[js]~pos[js],type="h",ylim=c(0,0.5))

idx<-search.optima(maf=maf,pos=pos,c=100,type="multH")
plot(maf[js]~pos[js],type="h",ylim=c(0,0.5))
```

---

select.Type *Select Bin type (e.g., Bin A, B, or C) of SNPs.*

Description

On Illumina, SNPs are binned according to the number and type of beads needed for a working assay. Bins A and B use 2 beads to deal with ambiguous bases (A/T or C/G) where the same dye is used for each base. Bin C uses 1 bead for assays that use two different dyes.

Usage

```
select.Type(x, oblLst, type = "A", nsel)
```

Arguments

`x`	An data frame which has map data for SNPs.
`oblLst`	A list of obligatory SNPs
`type`	Type of SNPs, which can be either "A", "B" or "C".
`nsel`	Number of SNPs to be selected.

Author(s)

Nick X-L Wu

---

summary.map *Generating summary statistics for a map object (data frame)*

Description

This functions generates summary statisics for a map object. It accepts a data frame as the input.

Usage

```
## S3 method for class 'map'
summary(x, outFile)
```

Arguments

`x`	A data frame which contains at least two columns, one for chromosomes (Chromosome) and the other for for allelic frequency. The column name can be either "Freq" or "Maf" (which is not case insensitive).
`outFile`	When the output file name is provided, the summary statistics are saved in this output file.

Value

It returns a table of summary statistics.

Author(s)

Nick X-L Wu

`unifBounds` *The unifBounds function*

Description

Thi function is used to get uniform boundary ticks

Usage

```
unifBounds(from, to, n)
```

Arguments

`from`	The starting point
`to`	The end point
`n`	The number of boundary tikcs to create

Author(s)

Nick X-L Wu

Examples

```
unifBounds(from=0,to=1,n=11)
```

Index

∗Topic **datasets**
demo60K, 8

addUnifBounds, 2
addUnifBounds,data.frame (addUnifBounds), 2
addUnifBounds,data.frame,missing,missing,missing-method (addUnifBounds), 2
addUnifBounds,integer-integer (addUnifBounds), 2
addUnifBounds,missing,numeric,numeric,numeric-method (addUnifBounds), 2
adjust.nInBin, 3
adjust.nInBin2, 3

cleanup.map, 4
comparePanels, 4
computeEntropy, 5
conditional_betaBounds, 6
conditional_unifBounds, 7

demo60K, 8

fillGaps, 9
findBlocks, 9

getBlocks, 10

H.Shannon, 10

loadLibrary, 11

mapToLst, 12
muH.Shannon, 12

nInBins, 13
nType, 14

optimalCore, 14
optimalPanel, 16
optimizationMethods, 17

percent, 18
plot.map, 19

randomPanel, 19
readData, 20
readMap, 21
remove.Type, 21

search.optima, 22
select.Type, 23
summary.map, 23

unifBounds, 24
