# Supplementary material for: Optimal Design of Low-Density SNP Arrays for Genomic Prediction: Algorithm and Applications
Source: PLoS One. 2016 Sep 1;11(9):e0161719. doi: 10.1371/journal.pone.0161719 (PMC5008792; doi:10.1371/journal.pone.0161719)
Supplement: S2 File — This is the vignette for the selectSNP package (trial version 1.1). (PDF) [file pone.0161719.s005.pdf]

selectSNP (V1.06): An R package for optimal design of low-density SNP chips using a multiple-objective, local optimization (MOLO) algorithm (Users' Manual)

Nick X-L Wu (nwu@neogen.com) and Jiaqi Xu (jiaqi.xu@huskers.unl.edu)

03/31/2016

1 Introduction

Low-density (LD) single nucleotide polymorphism (SNP) arrays provide a cost-effective solution for genomic prediction and selection, and optimal algorithms and computational tools are in need. This trial version of the selecctSNP R package implements a multiple-objective, local optimization (MOLO) algorithm for optimal design of LD SNP chips. A heuristic, local search algorithm is used to find the local optima, which approximate the global optimum.

The following example data frame is included in this package, which include which consists of 60,472 SNPs for demonstration. This data frames have columns for SNP names (SNP), chromosome IDs (Chromosome), map positions (Position), minor allele frequencies (Maf), type of each SNP (Type), and status of each SNP (Status; whether or not it is an obligatory SNP that must be included in the optimal panel).

- *demo60K*

2 Installation

There are two ways to install the package *selectSNP_1.06.tar.gz* or package *selectSNP_1.06_R_x86_64-redhat-linux-gnu.tar.gz*:

- RStudio > install.packages("package_path/selectSNP_1.00.tar.gz",repos=NULL,type="source")

Note: “package_path” is a location where the program package file “selectSNP_1.00.tar.gz” is stored.

The processes of installation are shown as follows:

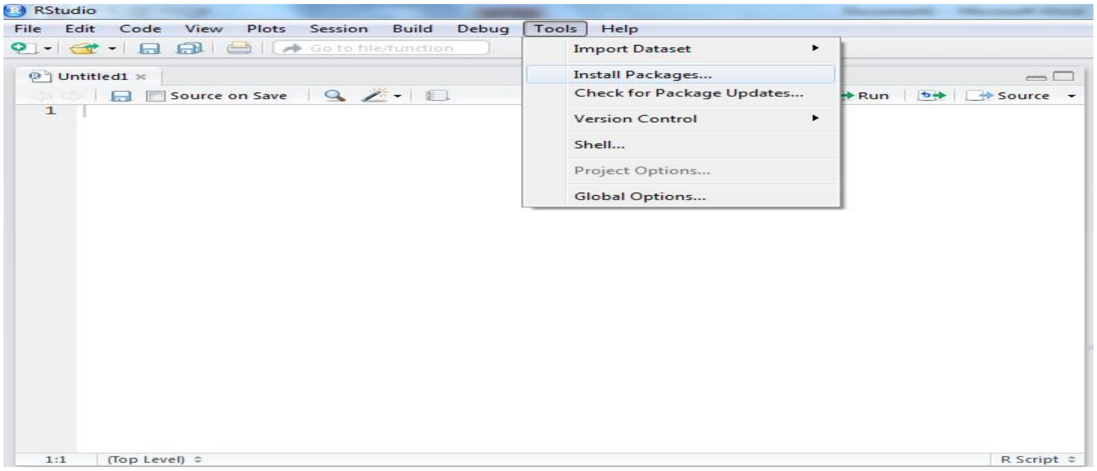
RStudio
File Edit Code View Plots Session Build Debug Tools Help
Untitled1
Source on Save
Import Dataset
Install Packages...
Check for Package Updates...
Version Control
Shell...
Project Options...
Global Options...
Run
Source
1:1 (Top Level)
R Script

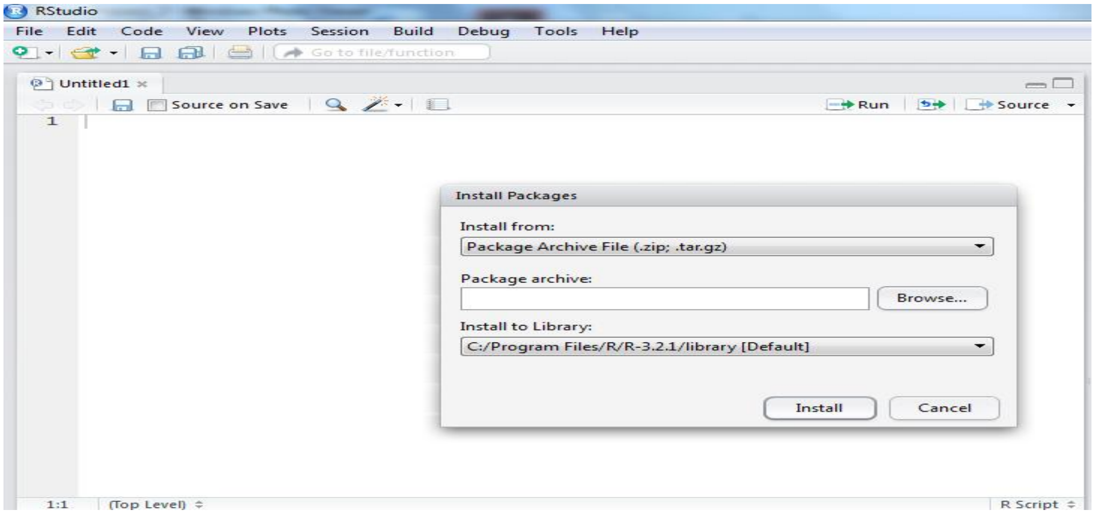
RStudio
File Edit Code View Plots Session Build Debug Tools Help
Untitled1
Source on Save
Run
Source
Install Packages
Install from:
Package Archive File (.zip; .tar.gz)
Package archive:
Browse...
Install to Library:
C:/Program Files/R/R-3.2.1/library [Default]
Install
Cancel
1:1 (Top Level)
R Script

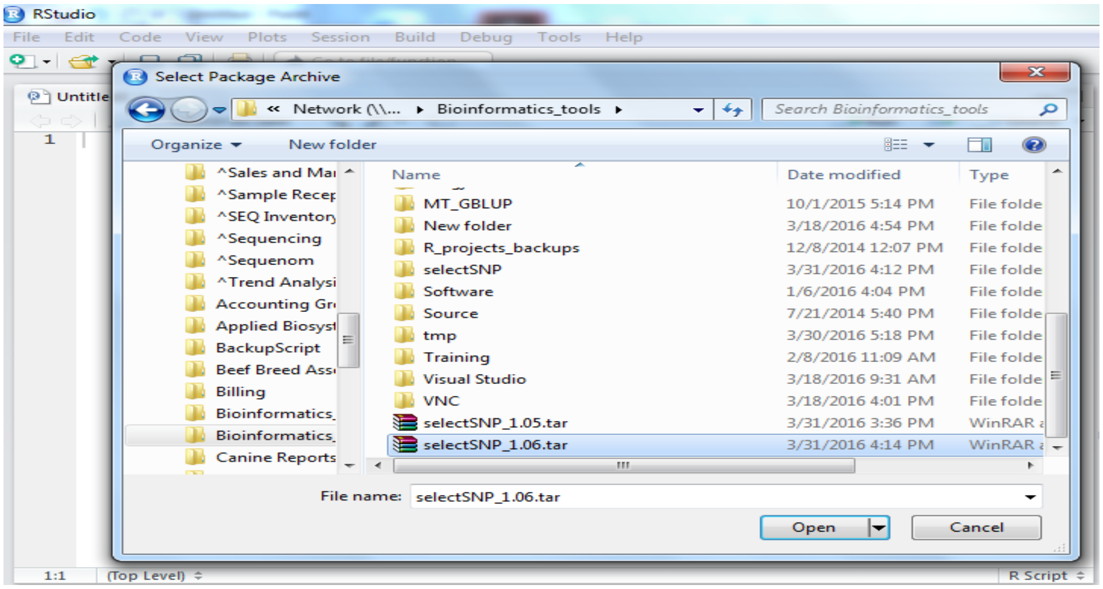
RStudio
Select Package Archive
« Network (\\... ▸ Bioinformatics_tools ▸
Search Bioinformatics_tools
Organize
New folder
Name
Date modified
Type
MT_GBLUP 10/1/2015 5:14 PM File folde
New folder 3/18/2016 4:54 PM File folde
R_projects_backups 12/8/2014 12:07 PM File folde
selectSNP 3/31/2016 4:12 PM File folde
Software 1/6/2016 4:04 PM File folde
Source 7/21/2014 5:40 PM File folde
tmp 3/30/2016 5:18 PM File folde
Training 2/8/2016 11:09 AM File folde
Visual Studio 3/18/2016 9:31 AM File folde
VNC 3/18/2016 4:01 PM File folde
selectSNP_1.05.tar 3/31/2016 3:36 PM WinRAR a
selectSNP_1.06.tar 3/31/2016 4:14 PM WinRAR a
File name: selectSNP_1.06.tar
Open
Cancel

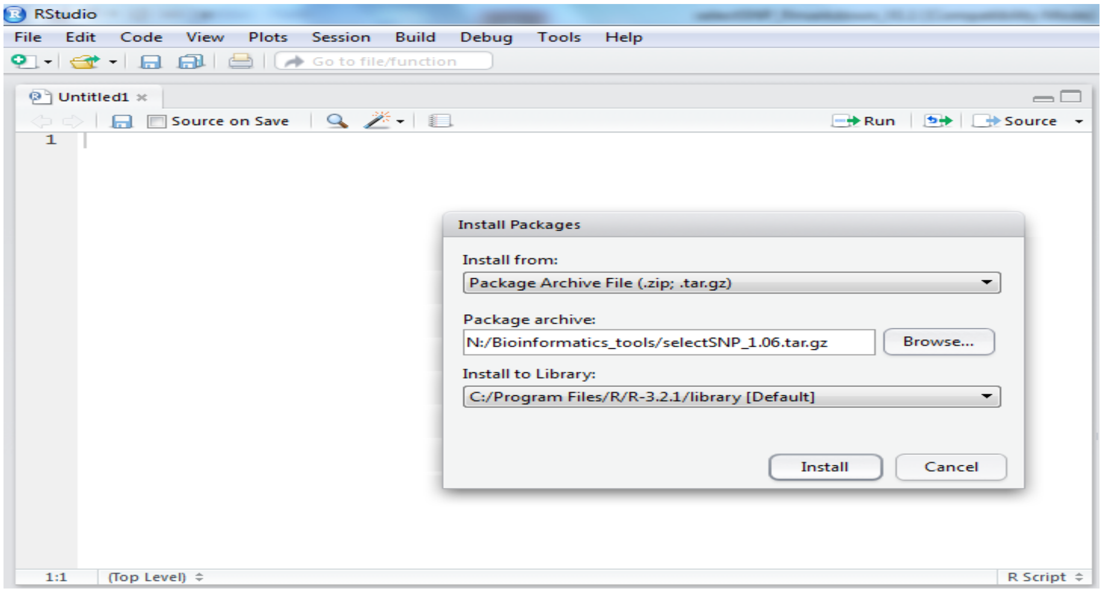

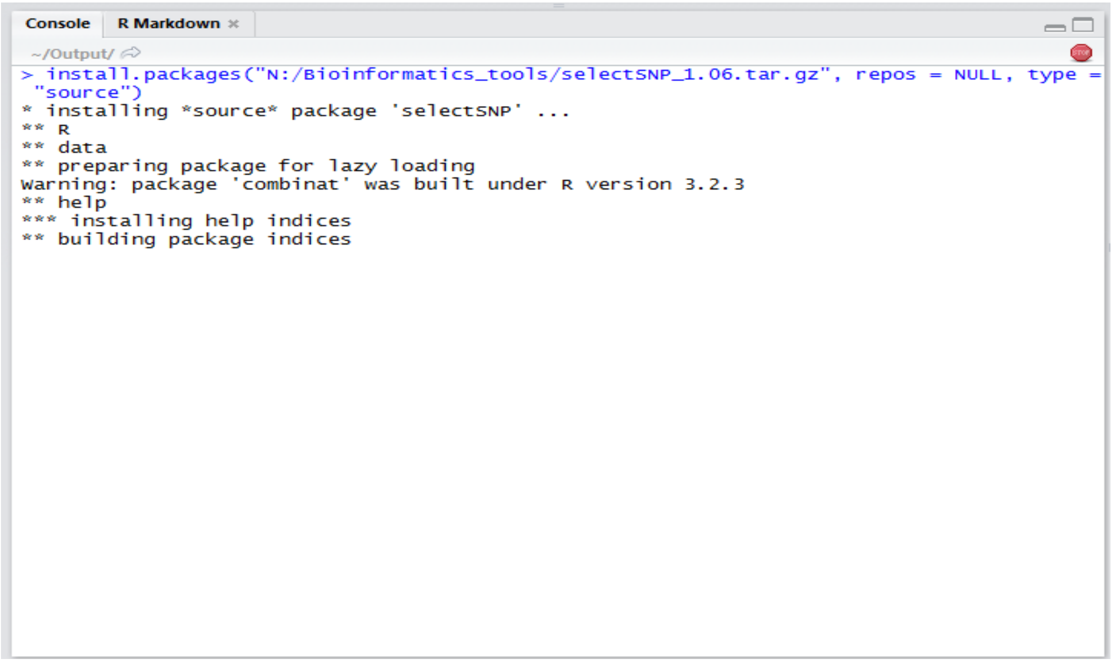

- Linux > R CMD INSTALL selectSNP_1.06_R_x86_64-redhat-linux-gnu.tar.gz [enter]

User may install one of them to the default library.

To load this package in R, simply type:

```
library("selectSNP")
```

Attaching package: ‘selectSNP’

```
data(demo60K)

plot.map(demo60K)
```

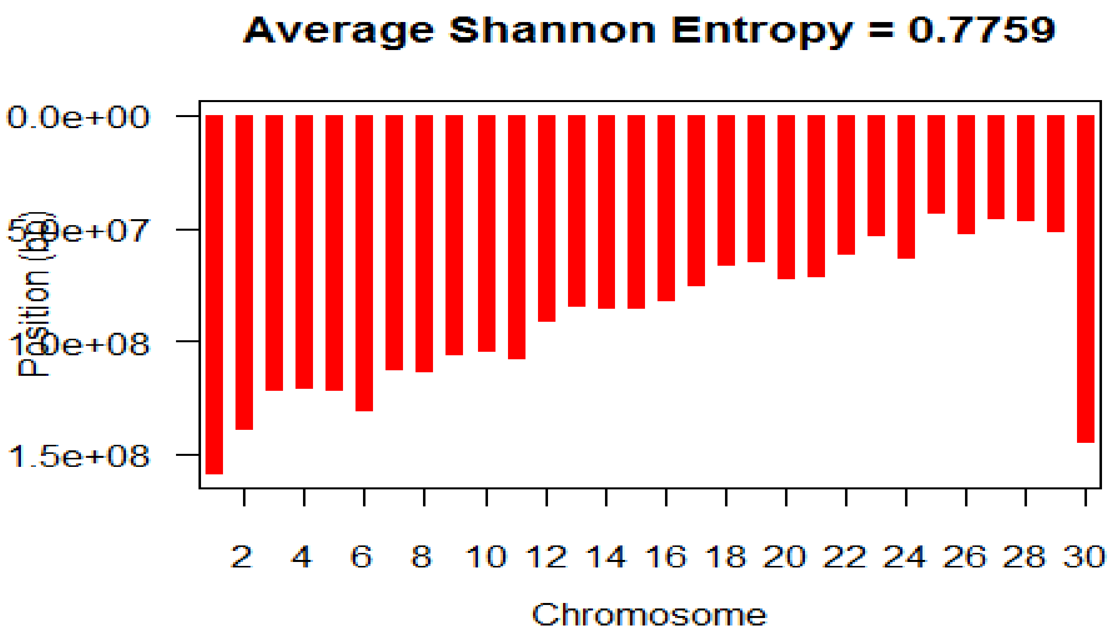

4 Main functions

4.1 OptimalPanel

This function selects a panel of SNPs based on either a uniform model or a beta model, optimized using a heuristically local optimization algorithm. The main parameters are as follows:

- *allsnp* = A data file or data frame as the source SNP database, from which a subset of SNPs are to be selected. It must include the following columns: SNP (SNP names), Chromosome (chromosome index), Position (map position), Maf (minor allele frequency), Type (SNP type) and Status (status of usage; is an obligatory SNP?).
- *oblsnp* = A data file or a data frame for "obligatory" SNPs, if provided. The data frame must contain columns for SNP names (SNP), chromosome index (Chromosome), map position (Position), minor allele frequency (Maf), SNP type (Type) and status of usage (Status).
- *cansnp* = A data file or a data frame for candidate SNPs, if provided. This set of SNPs needs to be duplicated three times, as required by the manufacture. The data frame must have columns for SNP names (SNP), chromosome index (Chromosome), map position (Position), minor allele frequency (Maf), SNP type (Type) and status of usage (Status).
- *nCSet* = How many sets of candidate genes are to be included? Its default value is 3, meaning that candidate genes are duplicated three times.
- *maf_cutoff* = cutoff value for minor allele frequency, which by default is 0.05.
- *prefix* = A prefix to be added to the output panel name.

- *pnlSize* = Panel size, that is, the number of SNPs to be selected. For example, if the target panels will contain 1000 SNPs, then set pnlSize = 1000.
- *method* = Statistical model for initial distribution of virtual framework (VF) SNPs, which can be either uniform (Unif) or Beta.
- *tailed* = This parameter defines whether and how the SNPs are to be enriched on both ends of the chromosomes, empirically following a Beta distribution. It has three options: "more", "less" or "formula". Mathematically, it divides the whole chromosome into 20 bins. Denote the first two bins on the left end as lftB, the last two bins on the right end as the rgtB, and the 16 bins in between as MidB. If "more" is chosen, it allocates 29% of SNPs on either leftB or rgtB, and 42% of the SNPs on the MidB. The "less" option allocates less SNPs on both ends, that is, 22%, 56% and 22% on lftB, MidB, and rgtB, respectively. When "formula" is chosen, it allocates SNPs to each of the 20 bins based on a Beta(a,b) distribution. Typical values for a and b are: a = b = 0.5.
- *r.bw* = This parameter defines the local search radius centered at VF SNP. The "local region" is characterized by its bandwidth (bw), that is, a subset within a region: abs(x - center_location) <= bw, where bw is calculated as: (max(x) - min(x))/(length(x)-1). Its default value is set up to be 0.50, which gives a full coverage of the whole genome.
- *nBacSNP* = Number of slots need to be reserved for bacteria SNPs. Its default value is zero.
- *nYSNP* = Number of slots reserved for SNPs on Y chromosome. Its default value is zero.
- *nA* = Minimum number of Bin-A type SNPs to be included. For the manufacture, SNPs are binned according to the number and type of beads needed for a working assay. Bins A and B use 2 beads to deal with ambiguous bases (A/T or C/G) where the same dye is used for each base. Bin C uses 1 bead for assays that use two different dyes.
- *nB* = Miniumum number of Bin-B type SNPs to be included.
- *c* = The paramater that empirically tunes the weights for adjusting Shannon Entropy according to SNP distributions on the maps. The larger c, the smaller weights, and the less adjustment will be made. By default, c = 1, which exercises the adjustment.
- *m* = The paramete that specifies how many SNP to be selected on each bin.
- *type* = Type of method used to optimize the information, either on single-SNP basis (singH) or multiple-SNP basis (multH). By default, type = singH.

Example 1

This example shows how to generates a panel of `1000` SNPs (pnlSize=1000) from the example 60K SNP panel (allsnp=demo60K) based on a `uniform` model (method="uniform") without optimization (r.bw=0). Letting r.bw = 0 turns off local optimization search.

```
data(demo60K)

U50<-optimalPanel(allsnp=demo60K,
                  pnlSize=1000,
                  method="uniform",
                  maf_cutoff=0,
                  r.bw=0)

plot.map(U50)
```

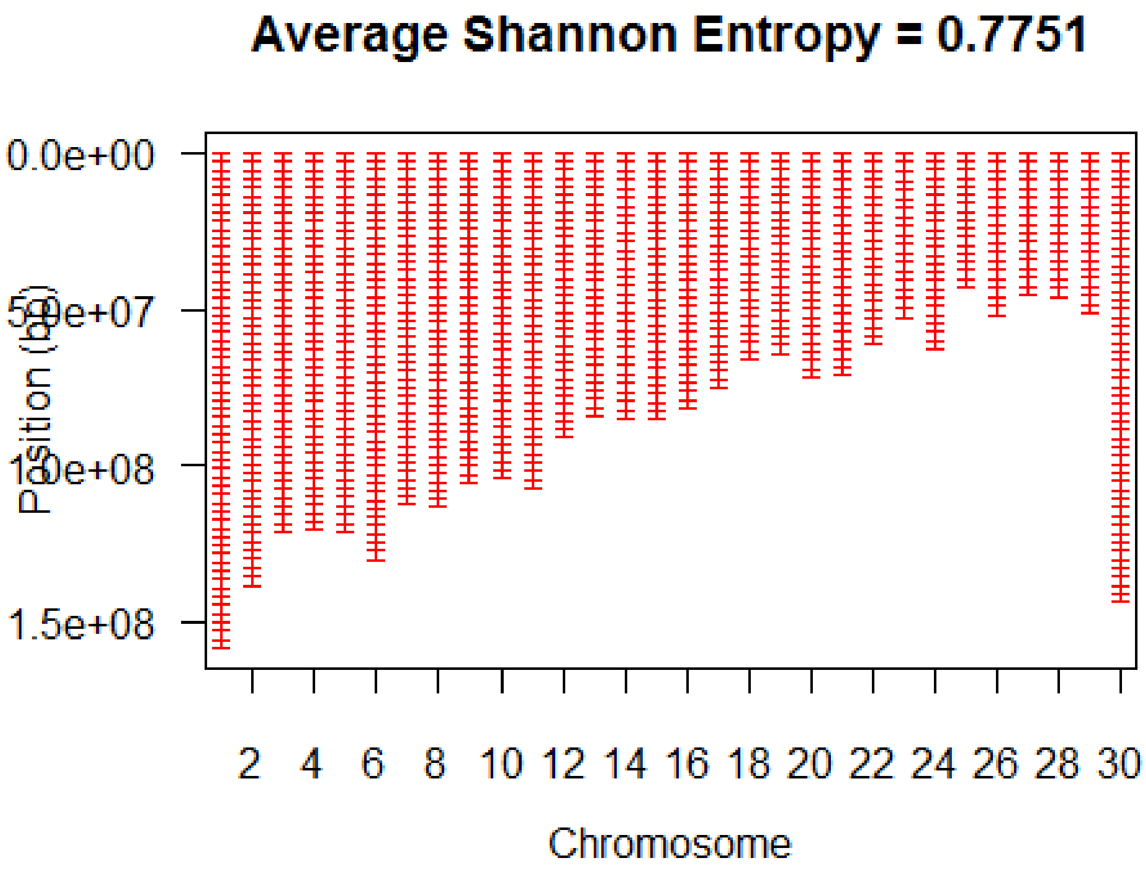

Example 2

This example shows how to obtain a panel of 1000 SNPs (pnlSize=1000) from the example 60K SNP panel (allsnp=demo60K) based on an uniform model (method="uniform") with optimization. Letting r.bw = 0.50 allows for local optimal search covering the whole genome. System information (Shannon entropy) was evaluated on single-SNP bassis (type=singH).

```
data(demo60K)
U50s<-optimalPanel(allsnp=demo60K,
                  pnlSize=1000,
                  method="uniform",
                  maf_cutoff=0,
                  r.bw=0.5,
                  type="singH")

plot.map(U50s)
```

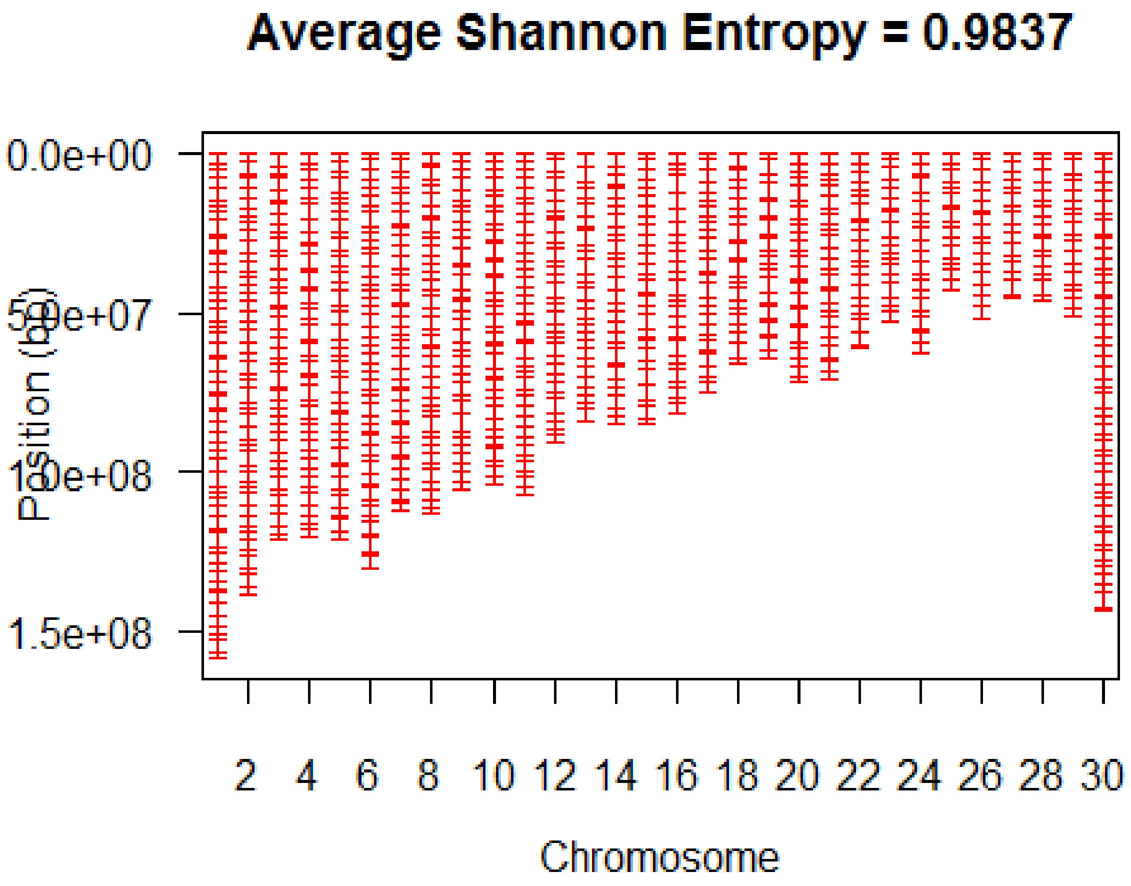

Example 3

This example shows how to select a panel of `1000` SNPs (pnlSize=1000) from data *demo60K* (allsnp=demo60K) based on a `uniform` model (method="uniform") with optimization (r.bw = 0.5). The system information was evaluated on multiple-SNP basis (type = multH) and adjusted for the uniformness of SNP locations on the maps (c=1).

```
data(demo60K)
U50m<-optimalPanel(allsnp=demo60K,
                   pnlSize=1000,
                   method="uniform",
                   maf_cutoff=0,
                   r.bw=0.5,
                   c=1,
                   type="multH")

plot.map(U50m)
```

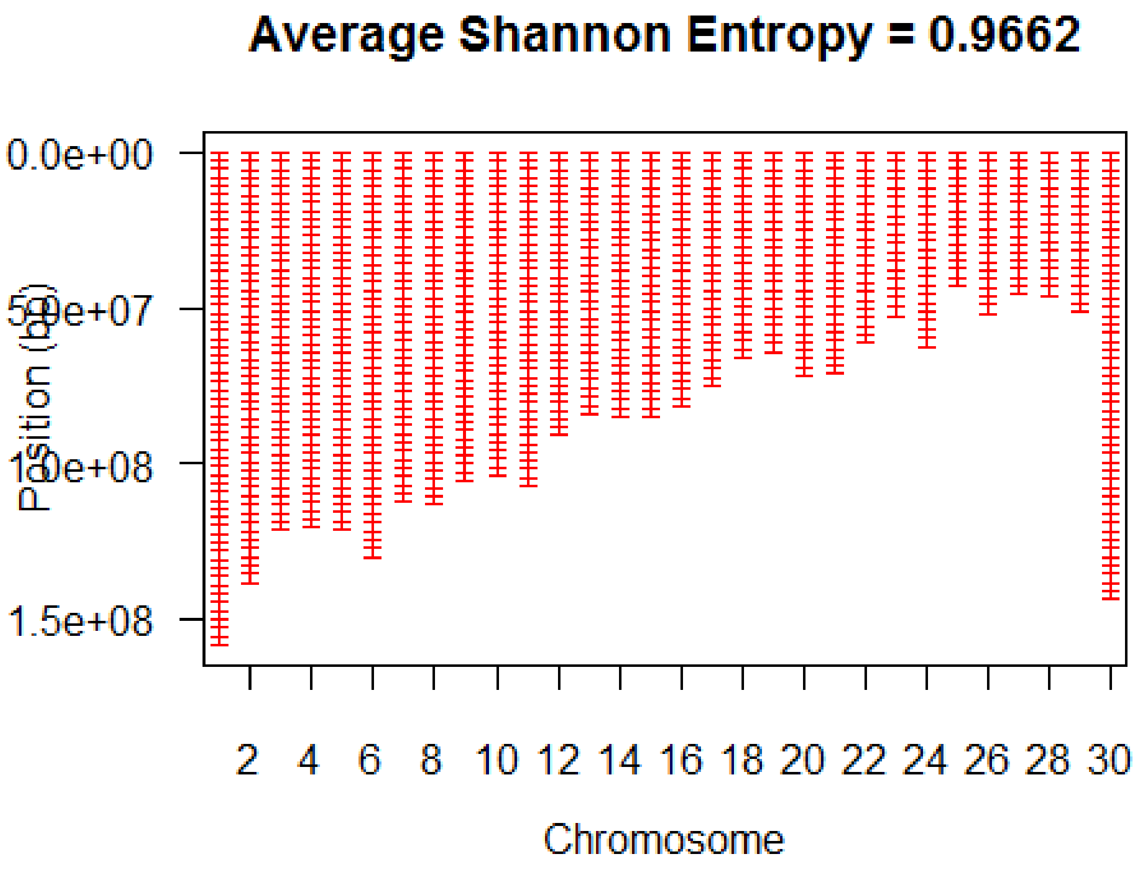

Example 4

This example shows how to select an optimal panel of 1000 SNPs (pnlSize=1000) from the example 60K SNP panel (allsnp=demo60K) based on an Beta model () with the local search tuning parameter set up to be r.bw = 0.50 and with slight SNP enrichment on chromosome ends (tailed = “less”). The system information is evaluated on multiple-SNP basis (type="multH") and adjusted for the uniformness of SNP locations on the maps (c=1).

```
data(demo60K)
B50<-optimalPanel(allsnp=demo60K,
                  pnlSize=1000,
                  method="Beta",
                  tailed="less",
                  maf_cutoff=0,
                  r.bw=0.5,
                  c=1,
                  type="multH")
```

```
plot.map(B50)
```

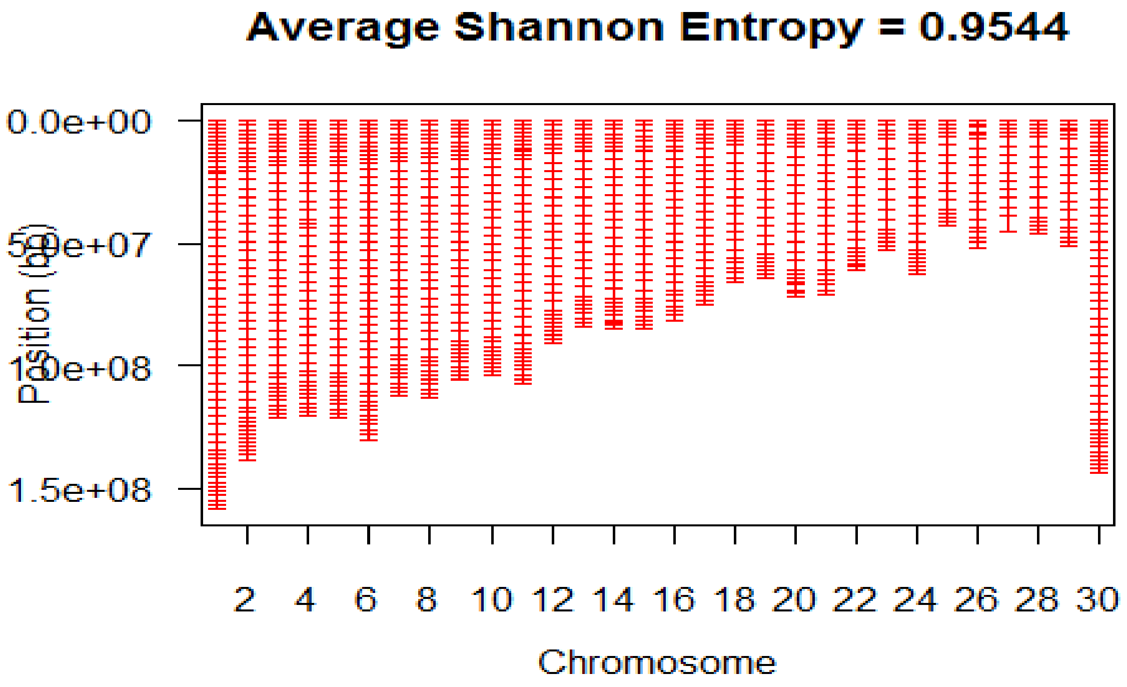

4.2 comparePanels

This function compares panels optimized using the uniform and beta models, respectivel, and with various settings of parameter values. Additionally, a random panel is included in the comparison as well. The main parameters are as follows:

- *allsnp* = Input data file or data frame that contains all SNPs from which the optimal panel is to selected. The data should contain the following columns: SNP, Chromosome, Position, Maf, Type, and Status.
- *oblsnp* = A data file or a data frame for the "obligatory" SNPs, if provided. The data frame must contain columns for SNP names (SNP), chromosome index (Chromosome), map position (Position), minor allele frequency (Maf), SNP type (Type) and status of usage (Status).
- *pnlSize* = Panel size, that is, the number of SNPs to be selected into the optimal panel.
- *method.set* = A set of methods, which by default include "uniform" and "beta".
- *rbin.set* = A set of bin intervals to be fed into the tuning parameter r.bw, which by default are values between 0 and 1, with an increment of 0.1.
- *c* = The paramater that empirically tunes the weights for adjusting Shannon Entropy according to SNP distributions on the maps. The larger c, the smaller weights, and the less adjustment will be made. By default, c = 1, which exercise the adjustment.
- *m* = The paramete that specifies how many SNP to be selected on each bin. This parameter does not necessarily need to be set up manually.

- *type* = Type of method to optimize the information, either on single-SNP basis (singH) or multiple-SNP basis (multH). By default, type = singH.

Example 5

This example illustrates how to run multiple jobs for generating SNP panels with varying parameter values (e.g., uniform model vs. beta model, with the local search tuning parameter varying from 0 to 1). The outcome are 22 selected SNP panels, plus a random panel as a non-optimization reference. Optimal panels can then be taken from the 22 selected SNP panels.

```
data(demo60K)
out<-comparePanels(allsnp=demo60K,
                   pnlSize=1000,
                   method.set=c("Uniform","Beta"),
                   rbin.set=seq(0,1,0.1),
                   type="singH")
out

     Method r.bin    ASE  Increm
 1  Uniform     0 0.8146   0.00%
 2  Uniform   0.1 0.9815  20.49%
 3  Uniform   0.2 0.9872  21.19%
 4  Uniform   0.3 0.9882  21.31%
 5  Uniform   0.4 0.9885  21.35%
 6  Uniform   0.5 0.9886  21.36%
 7  Uniform   0.6 0.9887  21.37%
 8  Uniform   0.7 0.9887  21.37%
 9  Uniform   0.8 0.9887  21.37%
 10 Uniform   0.9 0.9887  21.37%
 11 Uniform     1 0.9887  21.37%
 12    Beta     0 0.8198   0.64%
 13    Beta   0.1 0.9684  18.88%
 14    Beta   0.2 0.9748  19.67%
 15    Beta   0.3 0.9758  19.79%
 16    Beta   0.4 0.9760  19.81%
 17    Beta   0.5 0.9762  19.84%
 18    Beta   0.6 0.9762  19.84%
 19    Beta   0.7 0.9763  19.85%
 20    Beta   0.8 0.9763  19.85%
 21    Beta   0.9 0.9763  19.85%
 22    Beta     1 0.9763  19.85%
 23  Random    NA 0.7698  -5.50%
```
