# Supplementary material for: Optimal Design of Low-Density SNP Arrays for Genomic Prediction: Algorithm and Applications
Source: PLoS One. 2016 Sep 1;11(9):e0161719. doi: 10.1371/journal.pone.0161719 (PMC5008792; doi:10.1371/journal.pone.0161719)
Supplement: S1 Table — The column names are nLoci (number of SNPs on each chromosome), Length (physical map length, in base pairs, of each chromosome), max.bw (maximum gap, in base pairs, on each chromosome). Chromosome 30 stands for X Chromosome. (DOCX) [file pone.0161719.s006.docx]

| Chromosomes | **50K bovine chip** | | | **24K bovine chip** | | |
| --- | --- | --- | --- | --- | --- | --- |
|  | **nLoci** | **Length** | **max.bw** | **nLoci** | **Length** | **max.bw** |
| **1** | 3,430 | 158,094,120 | 516,643 | 1,398 | 158,199,469 | 490,973 |
| **2** | 2,829 | 136,662,110 | 533,790 | 1,252 | 138,187,781 | 1,208,269 |
| **3** | 2,549 | 121,144,180 | 729,996 | 1,145 | 121,323,587 | 440,624 |
| **4** | 2,570 | 120,624,834 | 336,473 | 1,028 | 120,624,834 | 473,131 |
| **5** | 2,271 | 121,078,748 | 518,023 | 1,086 | 121,144,374 | 493,126 |
| **6** | 2,575 | 119,078,000 | 1,601,814 | 1,052 | 129,996,307 | 7,062,456 |
| **7** | 2,352 | 112,384,068 | 1,123,527 | 1,000 | 112,579,733 | 618,416 |
| **8** | 2,429 | 113,010,018 | 481,649 | 986 | 113,037,056 | 438,569 |
| **9** | 2,095 | 105,517,929 | 666,907 | 980 | 105,603,151 | 475,857 |
| **10** | 2,206 | 104,173,193 | 1,962,287 | 917 | 104,173,193 | 494,043 |
| **11** | 2,295 | 107,177,910 | 505,412 | 983 | 107,206,532 | 512,741 |
| **12** | 1,773 | 90,944,479 | 1,125,627 | 778 | 90,944,479 | 523,254 |
| **13** | 1,850 | 83,862,908 | 615,150 | 784 | 83,916,674 | 443,688 |
| **14** | 1,831 | 83,181,185 | 489,270 | 777 | 83,748,424 | 455,306 |
| **15** | 1,762 | 84,444,611 | 662,972 | 779 | 84,444,611 | 471,105 |
| **16** | 1,726 | 81,249,445 | 979,329 | 790 | 81,604,419 | 420,958 |
| **17** | 1,600 | 74,977,060 | 813,742 | 677 | 75,035,198 | 419,333 |
| **18** | 1,376 | 65,726,027 | 647,494 | 632 | 65,821,775 | 434,548 |
| **19** | 1,420 | 63,541,206 | 299,445 | 619 | 63,578,968 | 460,266 |
| **20** | 1,568 | 71,594,660 | 517,120 | 753 | 71,864,640 | 415,676 |
| **21** | 1,483 | 71,097,978 | 763,726 | 680 | 71,365,786 | 472,537 |
| **22** | 1,324 | 61,216,181 | 376,168 | 545 | 61,216,181 | 465,568 |
| **23** | 1,093 | 52,225,085 | 836,641 | 516 | 52,405,469 | 456,420 |
| **24** | 1,312 | 62,493,634 | 431,384 | 567 | 62,493,634 | 372,910 |
| **25** | 1,004 | 42,803,940 | 282,660 | 389 | 42,825,176 | 403,035 |
| **26** | 1,116 | 51,582,714 | 536,748 | 445 | 51,582,714 | 442,849 |
| **27** | 981 | 45,332,323 | 914,351 | 411 | 45,633,498 | 487,325 |
| **28** | 980 | 46,182,968 | 345,188 | 418 | 46,182,968 | 457,921 |
| **29** | 1,086 | 51,486,008 | 729,891 | 452 | 51,486,013 | 459,378 |
| **30** | 1,170 | 148,616,760 | 3,778,029 | 905 | 148,776,314 | 1,010,434 |
