# Supplementary material for: Optimal Design of Low-Density SNP Arrays for Genomic Prediction: Algorithm and Applications
Source: PLoS One. 2016 Sep 1;11(9):e0161719. doi: 10.1371/journal.pone.0161719 (PMC5008792; doi:10.1371/journal.pone.0161719)
Supplement: S2 Table — The column names are N (number of SNPs per chromosome), and mean, SD (standard deviation), Min (minimum value), and Max (maximum value) of SNP spacing, where SNP spacing is defined as the map distance in base pairs between two adjacent SNPs on each chromosome. (DOCX) [file pone.0161719.s007.docx]

| Chromosome | N | Length | SNP spacing | | | |
| --- | --- | --- | --- | --- | --- | --- |
|  |  | (base pairs) | Mean | SD | Min | Max |
| 1 | 4519 | 158787993 | 35138 | 20233 | 0 | 535471 |
| 2 | 3921 | 136873311 | 34908 | 18569 | 0 | 232039 |
| 3 | 3571 | 123123281 | 34479 | 29877 | 0 | 1362367 |
| 4 | 3405 | 120598157 | 35418 | 18215 | 0 | 207572 |
| 5 | 3540 | 125027181 | 35318 | 67729 | 0 | 3882807 |
| 6 | 3412 | 122472975 | 35895 | 56064 | 0 | 3072483 |
| 7 | 3218 | 112576524 | 34983 | 27328 | 0 | 1099995 |
| 8 | 3253 | 113346241 | 34844 | 20445 | 0 | 377911 |
| 9 | 3087 | 105660219 | 34227 | 18203 | 0 | 143454 |
| 10 | 3019 | 104229666 | 34525 | 20124 | 0 | 303409 |
| 11 | 3103 | 107234118 | 34558 | 17884 | 0 | 172449 |
| 12 | 2672 | 91101978 | 34095 | 25095 | 0 | 453945 |
| 13 | 2478 | 84196105 | 33977 | 19901 | 0 | 269493 |
| 14 | 2502 | 84553259 | 33794 | 23740 | 0 | 471231 |
| 15 | 2582 | 85188890 | 32993 | 18406 | 0 | 226131 |
| 16 | 2473 | 81619528 | 33004 | 21384 | 0 | 443546 |
| 17 | 2240 | 75109411 | 33531 | 23123 | 0 | 509683 |
| 18 | 2058 | 65967076 | 32054 | 22029 | 0 | 334671 |
| 19 | 2018 | 63963700 | 31697 | 17951 | 0 | 168041 |
| 20 | 2233 | 71909920 | 32203 | 17565 | 0 | 170417 |
| 21 | 2227 | 71489735 | 32101 | 20580 | 0 | 271219 |
| 22 | 1896 | 61239693 | 32299 | 17875 | 0 | 355005 |
| 23 | 1746 | 52449738 | 30040 | 21406 | 0 | 501309 |
| 24 | 1939 | 62542150 | 32255 | 17052 | 0 | 204124 |
| 25 | 1416 | 42825176 | 30244 | 15300 | 0 | 149559 |
| 26 | 1618 | 51547521 | 31859 | 18214 | 0 | 215466 |
| 27 | 1440 | 45369283 | 31506 | 23640 | 0 | 534557 |
| 28 | 1489 | 46205794 | 31031 | 16309 | 0 | 122929 |
| 29 | 1597 | 51383996 | 32175 | 19241 | 0 | 373797 |
| 30 | 2022 | 148776314 | 73579 | 78234 | 0 | 867961 |
