# Supplementary material for: Optimal Design of Low-Density SNP Arrays for Genomic Prediction: Algorithm and Applications
Source: PLoS One. 2016 Sep 1;11(9):e0161719. doi: 10.1371/journal.pone.0161719 (PMC5008792; doi:10.1371/journal.pone.0161719)
Supplement: S3 Table — The column names are N (sample size), and Min (minimum value), Q1 (25% quantile), Q3 (75% quantile), Max (maximum value), and Mean and SD (standard) deviation) of PTA. (DOCX) [file pone.0161719.s008.docx]

| Trait | N | Min | Q1 | Median | Q3 | Max | Mean (SD) |
| --- | --- | --- | --- | --- | --- | --- | --- |
| DPR | 7,012 | -6.10 | -0.40 | 0.60 | 1.60 | 7.0 | 0.60 (1.53) |
| FY | 7.012 | -77.0 | 15.0 | 32.0 | 48.0 | 107.0 | 30.88 (24.65) |
| MY | 7,012 | -2,610.0 | 186.0 | 598.0 | 983.0 | 2,776.0 | 560.4 (618.4) |
