# Supplementary material for: Optimal Design of Low-Density SNP Arrays for Genomic Prediction: Algorithm and Applications
Source: PLoS One. 2016 Sep 1;11(9):e0161719. doi: 10.1371/journal.pone.0161719 (PMC5008792; doi:10.1371/journal.pone.0161719)
Supplement: S4 Table — The reference population for imputation consisted of 7,012 Holstein animals, each genotyped by GGPHD 80K SNPs and the validation set had 2,639 Holstein animals also with 80K genotypes. For the purpose of evaluation imputation error, only selected 6K genotypes were kept while the genotypes of the remaining SNPs were all set to be missing. The last four rows are average, standard deviation (SD), minimum value, and maximum value of imputation accuracy across 30 chromosomes, where chromosome 30 is the X chromosome. (DOCX) [file pone.0161719.s009.docx]

|  | 6KA->80K | 6KB->80K | 6KC->80K |
| --- | --- | --- | --- |
| 1 | 6.20% | 4.36% | 4.77% |
| 2 | 7.21% | 4.56% | 7.54% |
| 3 | 4.23% | 2.42% | 3.68% |
| 4 | 3.83% | 2.93% | 3.71% |
| 5 | 3.22% | 1.97% | 2.78% |
| 6 | 6.26% | 4.62% | 5.95% |
| 7 | 7.75% | 6.53% | 6.21% |
| 8 | 5.36% | 3.79% | 4.47% |
| 9 | 5.92% | 3.63% | 5.74% |
| 10 | 11.71% | 7.56% | 10.59% |
| 11 | 4.50% | 2.79% | 4.63% |
| 12 | 5.35% | 4.18% | 4.75% |
| 13 | 3.17% | 2.03% | 2.68% |
| 14 | 3.15% | 1.34% | 2.79% |
| 15 | 5.09% | 2.87% | 4.32% |
| 16 | 6.73% | 4.36% | 4.78% |
| 17 | 5.17% | 3.64% | 4.80% |
| 18 | 1.85% | 1.40% | 1.68% |
| 19 | 3.04% | 2.73% | 2.43% |
| 20 | 5.61% | 2.97% | 3.93% |
| 21 | 5.39% | 3.93% | 5.05% |
| 22 | 4.77% | 3.30% | 4.10% |
| 23 | 3.30% | 2.71% | 3.54% |
| 24 | 8.03% | 5.43% | 6.48% |
| 25 | 2.16% | 1.76% | 2.18% |
| 26 | 4.12% | 3.64% | 3.77% |
| 27 | 10.89% | 5.25% | 5.56% |
| 28 | 5.23% | 4.46% | 4.43% |
| 29 | 7.22% | 2.99% | 7.97% |
| 30 | 2.42% | 2.21% | 2.24% |
| Mean | 5.30% | 3.55% | 4.59% |
| STD | 2.32% | 1.44% | 1.91% |
| Min | 1.85% | 1.34% | 1.68% |
| Max | 11.71% | 7.56% | 10.59% |
