# Supplementary material for: Optimal Design of Low-Density SNP Arrays for Genomic Prediction: Algorithm and Applications
Source: PLoS One. 2016 Sep 1;11(9):e0161719. doi: 10.1371/journal.pone.0161719 (PMC5008792; doi:10.1371/journal.pone.0161719)
Supplement: S5 Table — SNP effects for genomic prediction were estimated on original 80K SNP genotypes in the reference population of 7,012 Holstein animals. Genomic prediction accuracy for three quantitative traits, namely daughter pregnancy rate (PDR), fat yield (FY) and milk yield (MY), were evaluated in the validation set of 2,639 Holstein animals. Genomic-estimated breeding values were computed either based on the three sets of selected 6K SNPs, or on imputed 80K genotypes obtained from the three sets of 6K SNP genotypes. (DOCX) [file pone.0161719.s010.docx]

|  | Daughter pregnancy rate  (DPR) | | Fat yield  (FY) | | Milk yield  (MY) | |
| --- | --- | --- | --- | --- | --- | --- |
|  | PA | RPA | PA | RPA | PA | RPA |
| 6KA | 81.57% | 88.05% | 82.14% | 87.23% | 77.32% | 83.80% |
| 6KB | 82.18% | 88.71% | 82.61% | 87.73% | 78.11% | 84.65% |
| 6KC | 83.56% | 90.20% | 84.43% | 89.67% | 80.79% | 87.56% |
| 6KA->80K | 91.43% | 98.69% | 93.43% | 99.22% | 90.56% | 98.15% |
| 6KB->80K | 91.76% | 99.05% | 93.66% | 99.47% | 91.04% | 98.67% |
| 6KC->80K | 92.02% | 99.33% | 93.59% | 99.39% | 90.90% | 98.52% |
| Original 80K | 92.64% | 100% | 94.16% | 100% | 92.27% | 100% |
