# Supplementary material for: Optimal Design of Low-Density SNP Arrays for Genomic Prediction: Algorithm and Applications
Source: PLoS One. 2016 Sep 1;11(9):e0161719. doi: 10.1371/journal.pone.0161719 (PMC5008792; doi:10.1371/journal.pone.0161719)
Supplement: Supplementary file 9 [file pone.0161719.s001.docx]

1

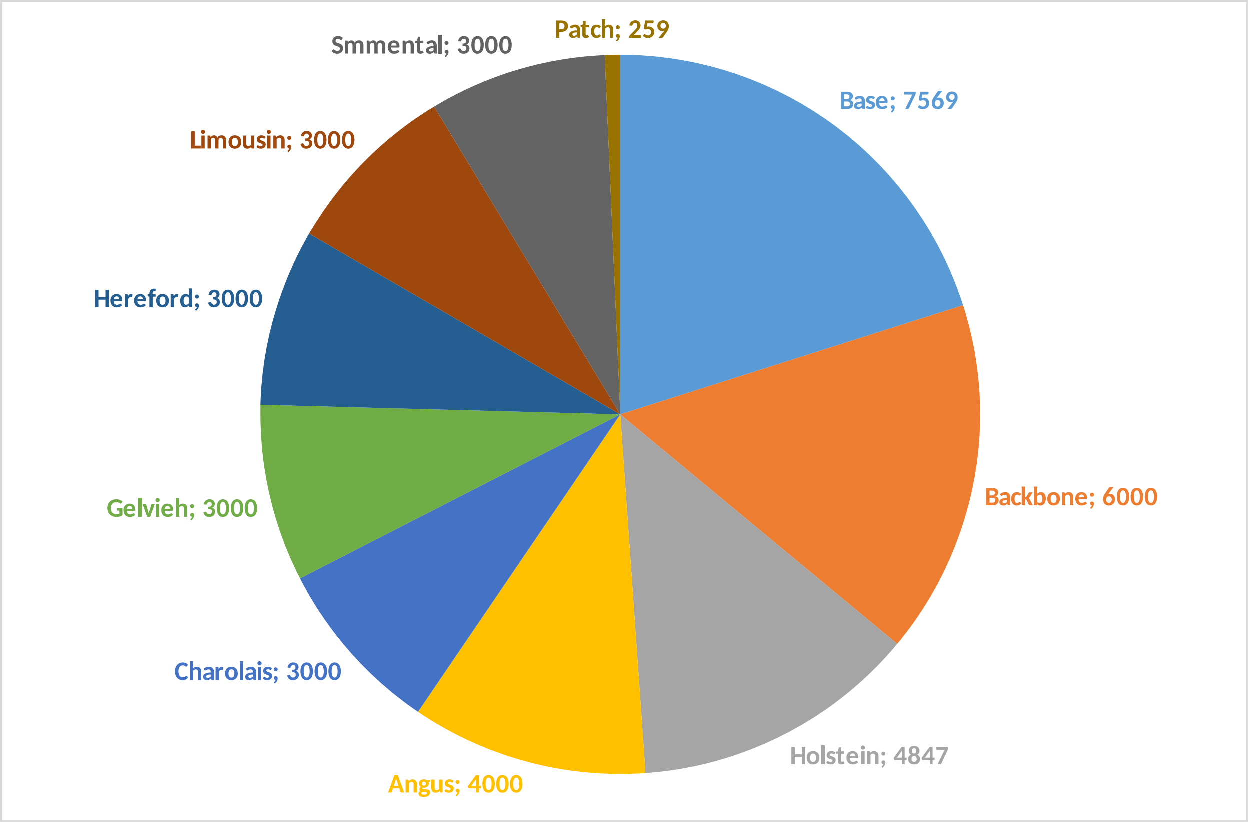
Patch; 259
Smmental; 3000
Base; 7569
Limousin; 3000
Hereford; 3000
Backbone; 6000
Gelvieh; 3000
Charolais; 3000
Holstein; 4847
Angus; 4000

2
