# Supplementary material for: Optimal Design of Low-Density SNP Arrays for Genomic Prediction: Algorithm and Applications
Source: PLoS One. 2016 Sep 1;11(9):e0161719. doi: 10.1371/journal.pone.0161719 (PMC5008792; doi:10.1371/journal.pone.0161719)
Supplement: Supplementary file 10 [file pone.0161719.s002.docx]

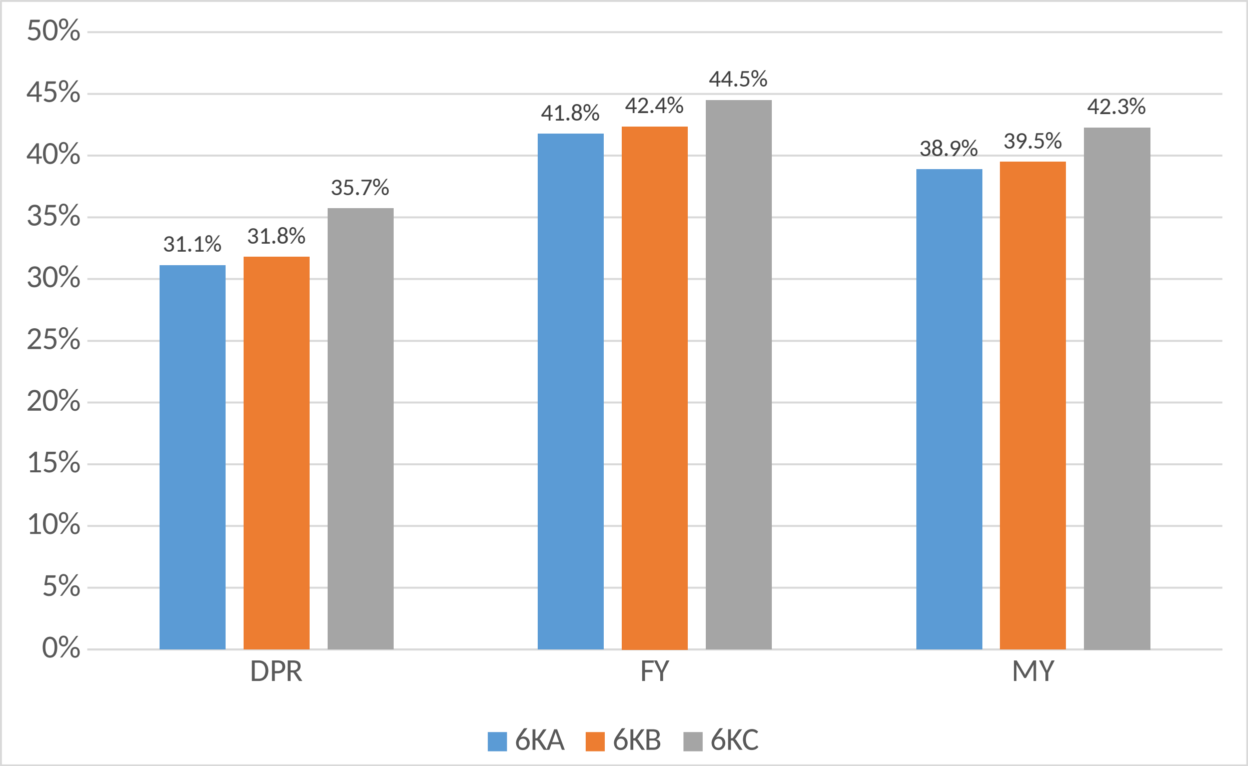
50%
45%
40%
35%
30%
25%
20%
15%
10%
5%
0%
31.1%
31.8%
35.7%
41.8%
42.4%
44.5%
38.9%
39.5%
42.3%
DPR
FY
MY
6KA
6KB
6KC
